# Scottish soldiers from the Battle of Dunbar 1650: A prosopographical approach to a skeletal assemblage

**DOI:** 10.1371/journal.pone.0243369

**Published:** 2020-12-21

**Authors:** Andrew R. Millard, Richard G. Annis, Anwen C. Caffell, Laura L. Dodd, Roman Fischer, Christopher M. Gerrard, C. Pamela Graves, Jessica Hendy, Lisa Mackenzie, Janet Montgomery, Geoff M. Nowell, Anita Radini, Julia Beaumont, Hannah E. C. Koon, Camilla F. Speller

**Affiliations:** 1 Department of Archaeology, Durham University, Durham, United Kingdom; 2 Archaeological Services, Durham University, Durham, United Kingdom; 3 KDK Archaeology Ltd, Leighton Buzzard, United Kingdom; 4 Nuffield Department of Medicine, Target Discovery Institute, University of Oxford, Oxford, United Kingdom; 5 Department of Archaeology, University of York, York, United Kingdom; 6 Department of Earth Sciences, Durham University, Durham, United Kingdom; 7 School of Archaeological and Forensic Sciences, University of Bradford, Bradford, United Kingdom; 8 Department of Anthropology, University of British Columbia, Vancouver, British Columbia, Canada; Museo delle Civiltà, ITALY

## Abstract

After the Battle Dunbar between English and Scottish forces in 1650, captured Scottish soldiers were imprisoned in Durham and many hundreds died there within a few weeks. The partial skeletal remains of 28 of these men were discovered in 2013. Building on previous osteological work, here we report wide-ranging scientific studies of the remains to address the following questions: Did they have comparable diet, health and disease throughout their lives? Did they have common histories of movement (or lack of movement) during their childhoods? Can we create a collective biography of these men? Strontium and oxygen isotope analysis of tooth enamel investigated childhood movement. Carbon and nitrogen isotope analysis of incrementally sampled dentine addressed childhood diet and nutrition. Metaproteomic analysis of dental calculus investigated oral microbiomes and food residues; this was complemented by microscopic analysis of debris in calculus from ingested materials. Selected individuals were examined for dental microwear. The extent of hydroxylation of proline in collagen was examined as a potential biomarker for scurvy. An osteobiography for each man was created using the full range of data generated about him, and these were synthesised using an approach based on the historical method for a collective biography or prosopography. The childhood residences of the men were primarily within the Midland Valley of Scotland, though some spent parts of their childhood outside the British Isles. This is concordant with the known recruitment areas of the Scottish army in 1650. Their diets included oats, brassicas and milk but little seafood, as expected for lowland rather than highland diets of the period. Childhood periods of starvation or illness were almost ubiquitous, but not simultaneous, suggesting regionally variable food shortages in the 1620s and 1630s. It is likely there was widespread low-level scurvy, ameliorating in later years of life, which suggests historically unrecorded shortages of fruit and vegetables in the early 1640s. Almost all men were exposed to burnt plant matter, probably as inhaled soot, and this may relate to the high proportion of them with of sinusitis. Interpersonal violence causing skeletal trauma was rare. Based on commonalities in their osteobiographies, we argue that these men were drawn from the same stratum of society. This study is perhaps the most extensive to date of individuals from 17^th^ century Scotland. Combined with a precise historical context it allows the lives of these men to be investigated and compared to the historical record with unprecedented precision. It illustrates the power of archaeological science methods to confirm, challenge and complement historical evidence.

## Introduction

**Fig 1:**
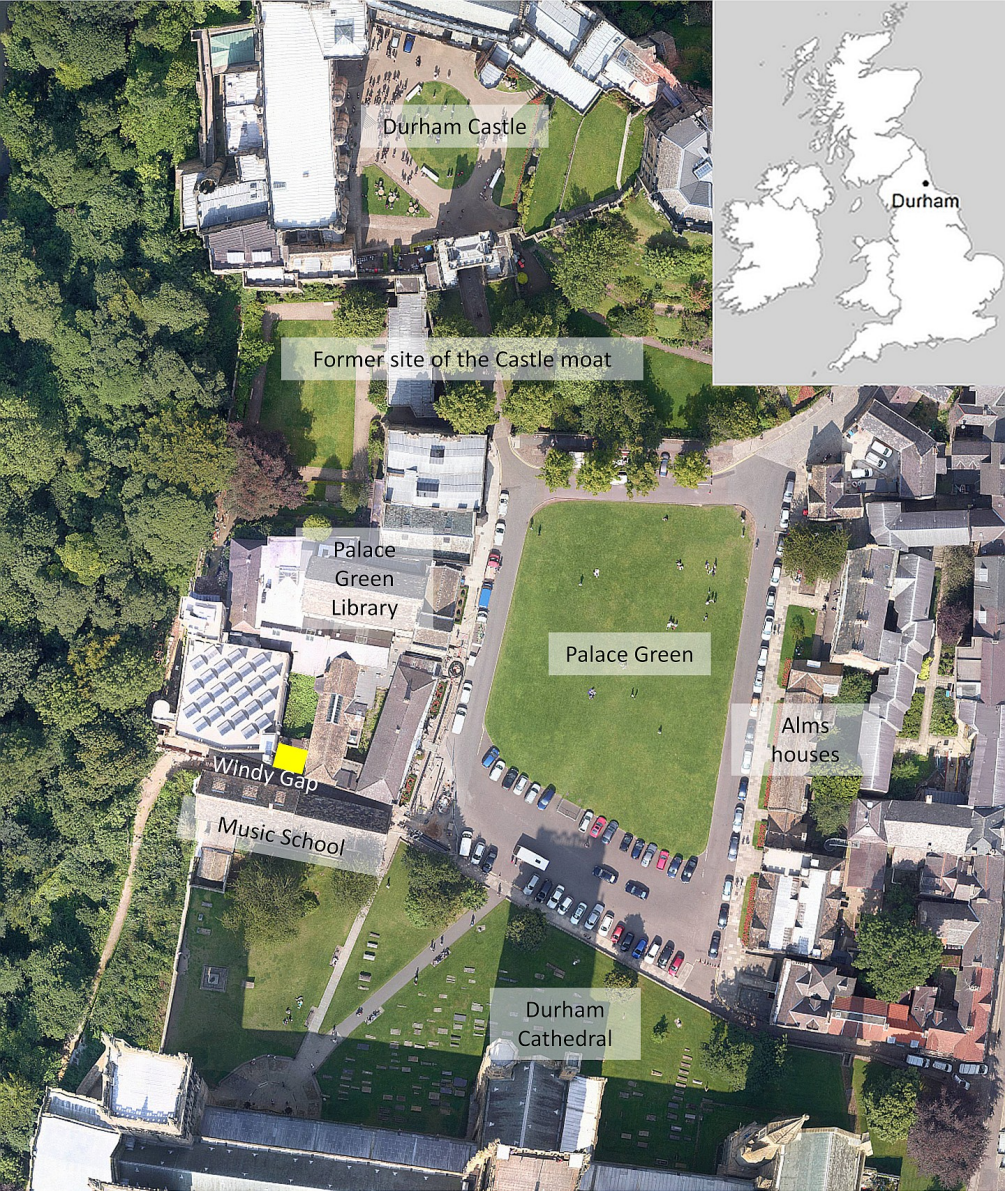
Site location showing Palace Green at the heart of Durham, with the Castle to the north and the Cathedral to the south. The excavation site is marked in yellow, north of Windy Gap. Photograph by courtesy of Purcell / Network Mapping.

In November 2013 human remains were discovered during construction work for a café in Palace Green Library, at the heart of the World Heritage Site in Durham, England (Fig 1). This discovery triggered a major research project, which identified the remains as those of Scottish soldiers captured in 1650 at the Battle of Dunbar, and investigated the background and aftermath of the battle and their imprisonment in Durham [1]. Here we synthesise historical, archaeological and scientific data to create a timeline that allows us to elaborate biographies of these long-deceased and anonymous individuals in unprecedented detail, and then to construct a collective biography or prosopography for the group as a whole.

Prosopography can be defined as “the study of biographical detail about individuals in aggregate” [2: 85]. Instead of offering individual case studies or generalising from widely dispersed examples, prosopography takes a well-defined population or social group, usually a group who have something in common with one another, and brings the data together in a systematic way to say something about the group, despite incomplete evidence for any one individual. Such research invariably collects a wide range of data but the focus is upon generalities in life histories, on revealing commonalities, connections and patterns in the data [3, 4]. The prosopographical approach has been applied to a wide range of historical groups, for example, the clergy of the Church of England 1780–1839 to examine how clergymen were educated and trained [5] and the members of the Senate in the final years of the Roman Republic, investigating how power shifted from the Senate to the Emperor Augustus [6].

Mays et al. [7: 694] have recently argued that the dominant population-based approach in human osteoarchaeology reduces each person to a datum in a collection of data and conceals “the richness of individual lived experience in the past”. Conversely, Hosek and Robb [8] have recently argued that the current approach to osteobiography is limited and there is a need for “a new, more humanistic bioarchaeology” that combines osteobiographies with population-level statistical studies. Robb has further developed this idea as “comparative osteobiographies” [9]. A prosopographical approach to an assemblage where all individuals belong to a specific group provides an unusual opportunity to address both these issues.

## Archaeological and historical background

**Fig 2:**
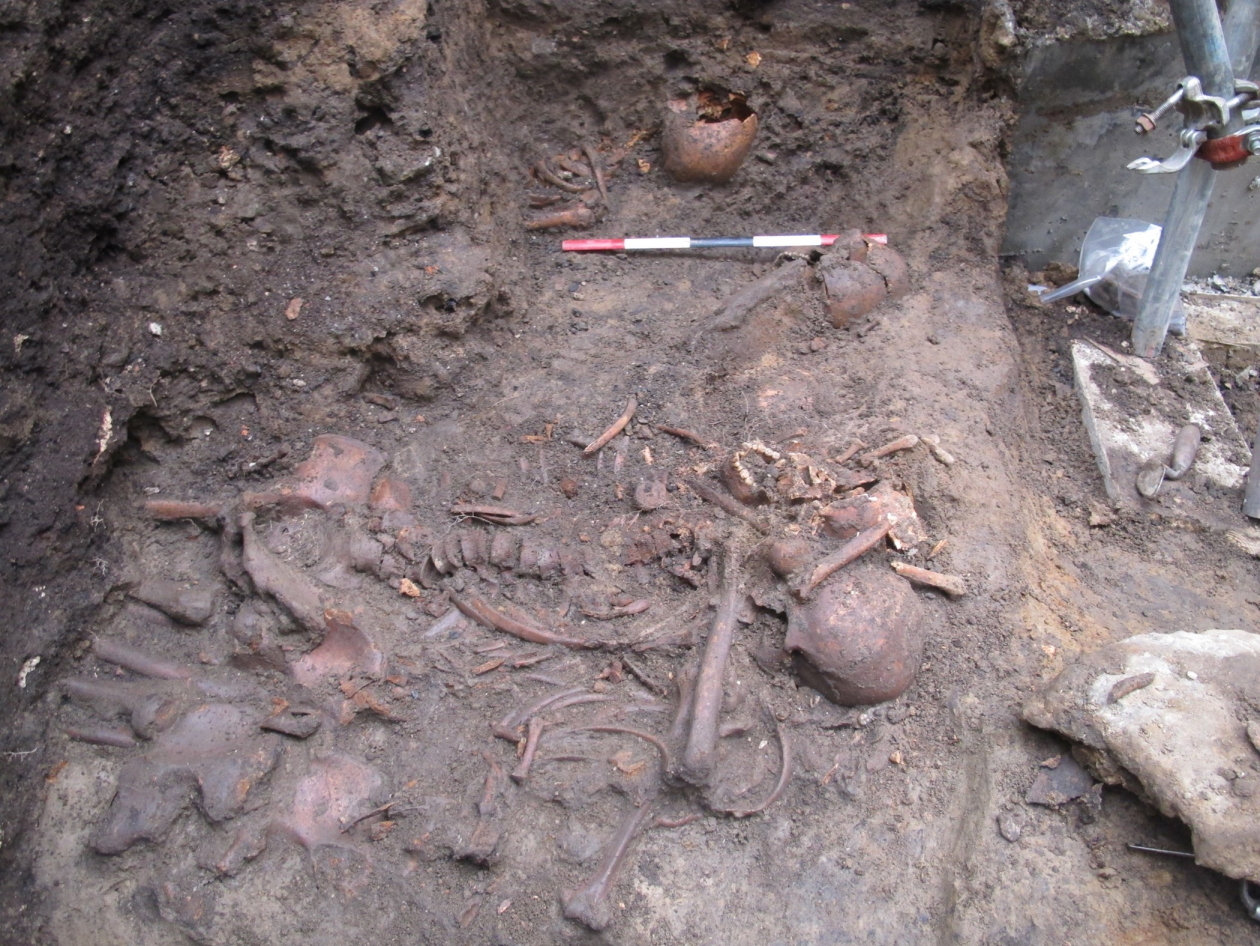
Skeleton 1 under excavation with left arm in unusual position, with partially exposed remains of other individuals illustrating how tightly packed the bodies were in feature F512. Scale 0.5m. North to the bottom.

The excavations at Palace Green Library revealed two burial pits. In an area of about 1.5×1.5 m (feature F512), 18 individuals were recovered, all but one laid out with heads to the south, but with limbs in a variety of positions (Fig 2). An additional 10 individuals were recovered from a second pit (feature F514) beneath the foundations of the neighbouring building and the wall separating the site from Windy Gap (S1A and S1B Methods). Three were buried with heads to the west, three with heads to the east, and one with their head to the south; the orientation of the remaining three was unclear. The varied dispositions of the skeletons indicate that the burials were not careful but hastily carried out. No finds were recovered with the bodies, and there was no trace of textiles, shoes or other personal possessions, suggesting that they had been buried without clothes or shrouds [1], and the positions of the arms of Sk 1 are not compatible with shroud burial (Fig 2). The limited excavation area, together with truncation by later features, meant that many of the bodies extended beyond the limits of excavation, and thus some individuals were represented only by legs and others by only by torso and head.

Subsequent osteological examination of the remains established that, for all where sex could be estimated, they were male, the majority being aged between 13 and 25 years of age, with two older adults and three adults of indeterminate age (Table 1). Although two possibly had healed fractures (rib and hand phalanx), one had a definite blade injury, one had evidence for soft-tissue injuries and there was one with some post-mortem damage while the bone was relatively fresh [1], there was remarkably little evidence for trauma on the skeletons. Initial strontium, lead and oxygen isotopic analysis (S1 Table) of the second molars of the 13 men with recovered dentitions showed a diverse set of origins, with only five consistent with origins in northern England, those five and an additional five consistent with origins in Scotland, and three who must have spent their childhoods outside the British Isles [1]. The burials were dated using three strands of evidence: (a) pipe-facets in the teeth of two men, indicating habitual tobacco smoking and thus placing them after the availability of cheap tobacco from Virginia plantations in 1612; (b) the stratigraphical position of the burial pits beneath one of the Bishop’s stables, first depicted on a map of 1754, and; (c) radiocarbon dating of the first and third molars of two men, giving samples spaced approximately ten years apart suitable for the application of wiggle-matching. The dating evidence was combined in a Bayesian model to produce an estimate of 1615–1620 and 1625–1660 at 95% probability [1] (summarised in S1 Methods).

**Table 1:** Summary of osteological data (from [1, 10]) and analyses conducted on each skeleton.

Sk No	Context	Age	Sex	Completeness	Dental Disease	Skeletal Pathology	Trauma	Dental Microwear	Micro-debris	Sr Pb, & O isotopes	Incremental dentine isotopes	Metaproteomics of calculus	Scurvy biomarkers
1	F512	14–15½	-	60–70%	Calculus; caries; DEH; enamel chips	Sinusitis; narrow palate; inflammation of ulna (woven bone) & femora (lamellar bone); transitional vertebrae; pilasterism & torsion of femora		X	X	X	X	X	X
2	F512	18–25	M?	10–20%	Calculus; periodontal disease; supernumerary incisor; possible pipe-smoking wear	Sinusitis; cribra orbitalia			X	X	X	X	X
3	F514	17–23	M	40–50%	-	Schmorl’s nodes; inflammation of both femora (lamellar bone)							
4	F514	16–18	-	40–50%	-	Inflammation of both femora and tibiae (woven and lamellar bone) and left second metatarsal (woven bone)							
5	F512	17–23	M	50–60%	Calculus; enamel chips; unusual wear	Schmorl’s nodes; sinusitis; transitional vertebra			X	X	X	X	X
6	F514	46+	M	60–70%	Calculus; supernumerary tooth; enamel chips	Schmorl’s nodes; OA of spine; OA of clavicles & right hip; Thoracic vertebrae 7–8 fused, large osteophytes between thoracic vertebrae 10–11; ossified cartilage; possible cysts in frontal and occipital bones; transitional vertebra	Possible soft-tissue trauma to nuchal crest;		X	X	X	X	X
7	F514	16–19	-	c. 5%	-	Inflammation of left femur (lamellar bone); possible residual rickets							
8/16A	F514	13–15	-	10–20%	-	Inflammation of left femur (lamellar bone), right tibia and both first metatarsals (transitional woven-lamellar bone)							X
9	F512	16–18	(M?)	5–10%	-	-							
10/11	F514	16–18	-	10–20%	-	Oval hollow in right metatarsal; bowed left fibula							
12	F512	17–23	M?	40–50%	Calculus; caries; DEH; 1 tooth NP/U; possible pipe-smoking wear; groove in lower incisor	Schmorl’s nodes; sinusitis; endocranial bone formation (transitional woven-lamellar bone)	Possible healed rib fracture		X	X	X	X	X
13	F514	18–25	M?	20–30%	-	Pilasterism, slight bowing and torsion of right femur							
14	F514	12–16	-	5–10%	-	Bones light and fragile, porous; trabecular bone in medullary cavities; inflammation of right tibia and fibula & first and fifth metatarsals (lamellar bone)							X
15	F512	16–20	M	5–10%	-	-							
16C	F514	18+	U	c.5%	-	Thin sharp metatarsal shafts							
17/16B	F514	18+	U	5–10%	-	Inflammation of both tibiae (lamellar bone)	Possible soft tissue trauma to left calcaneus						
18	F512	17–23	M	5–10%	-	-							
19	F512	18–25	M	60–70%	Calculus; caries; DEH; abscess; uneven wear; enamel chips; slight crowding and rotation of teeth	-		X	X	X	X	X	X
20	F512	18+	U	5–10%	-	-							
21	F512	18–25	M	40–50%	Calculus; pipe-smoking wear	Schmorl’s nodes; sinusitis; developmental anomaly of cervical vertebra 5; endocranial bone formation (transitional woven-lamellar bone); slight bowing of radii and right humerus	Linear post-mortem damage to cranium while bone still ‘fresh’		X	X	X	X	X
22	F512	18–25	M	30–40%	Calculus; caries; DEH; periodontal disease; abscesses; fractured molar; enamel chips; 3 teeth NP/U; rotated molar	Schmorl’s nodes; cribra orbitalia	Small healed blade injury in frontal bone		X	X	X	X	X
23	F512	17–19	(M?)	50–60%	Calculus; DEH; periodontal disease	Inflammation of mandible (woven bone), left humerus & right femur (lamellar bone); bowed humeri & right femur			X	X	X	X	X
24	F512	17–18	(M?)	30–40%	Calculus; caries; DEH	Sinusitis; subtle bowing of left humerus				X	X		X
25	F512	15–17	-	10–20%	Calculus; caries; DEH; probable abscess; possible pipe-smoking wear	Inflammation of mandible (transitional woven-lamellar bone)		X	X	X	X	X	X
26/27C	F512	14–16	-	10–20%	-	Small hollow area in proximal left tibia							
27A	F512	36–45	M	20–30%	AMTL; Calculus; DEH; rotated teeth; diastema; impaction of permanent canine & retention of deciduous canine; fractured molar	Sinusitis			X	X		X	X
27B	F512	16–18	(M?)	30–40%	-	-							
28	F512	16–20	M?	40–50%	Calculus; caries; DEH; 2 teeth NP/U; periodontal disease; unusual wear patterns & amount of wear in excess of that expected for developmental age; enamel chips; notch in upper incisor	Possible cribra orbitalia; sinusitis; depression on left parietal bone; shallow olecranon fossa of right humerus		X	X	X	X	X	X

Abbreviations: DEH: dental enamel hypoplasia, OA: osteoarthritis, NP: not present, U: unerupted, AMTL: ante-mortem tooth loss.

This archaeological evidence, in particular the distinctive demographic profile typical of military graves and the dating evidence for the burial pits, is entirely consistent with the identification of the human remains as prisoners taken at the Battle of Dunbar, Scotland, in 1650. After King Charles I was captured in 1648 and executed the following year on the orders of the English Parliament, England declared itself a republic without a king; Scotland, however, remained committed to the future Charles II, son of Charles I. With confrontation between the former allies now inevitable, Oliver Cromwell invaded Scotland in the summer of 1650 [11]. The Scots hastily raised an army, mustering men from many parts of the country and including foreign mercenaries, but many of the soldiers were inexperienced recruits, just as the archaeological evidence confirms. At the Battle of Dunbar on 3 September 1650 the Scots were routed after they decamped from their commanding position on Doon Hill and Cromwell launched a pre-emptive strike [12–14]. Many of the Scots retreated to Edinburgh, but Cromwell captured a large number of prisoners, possibly 9–10,000 men according his own estimates. The elderly and injured were released, which explains the presence of only one individual over 45 years old among the human remains, and Cromwell estimated that 4,000 soldiers were marched south into England. Many died or escaped their escort along the way. On the march they were housed for one night in St Nicholas’ Church in Newcastle, and the officers remained in Newcastle [15: 354]. After eight days the common soldiers reached Durham, where the Governor of Newcastle, Sir Arthur Hesilrige, reported that 3,000 men were imprisoned in the Cathedral [16], which was at that time standing empty after the Puritan government had suppressed all Bishops in 1646 and Cathedral Chapters in 1649 [17].

Like both armies before the battle [18], and many others since, the prisoners in Durham Cathedral suffered from dysentery, and many of them were moved to the Castle to be nursed. Nevertheless, by 31 October over 1,600 had died [16], implying a death rate of over 30 per day. Dysentery can therefore be assumed as the main cause of death. There is no record of their place of burial, though an account from 1655 or 1656 describes them as ‘thrown into holes by great numbers together in a moste Lamentable manner’ [19].

In short, the evidence from Palace Green is entirely congruent with what is known of the Dunbar prisoners, and not with any other historical context, for example, with plague a more representative sample of the population might be expected [1]. They represent men, primarily from Scotland, who were born between c.1590 and 1636. In the remainder of this paper, we aim to leverage this well-defined historical context and combine it with a wide range of scientific methods and techniques to create osteobiographies of these men, and collectively a prosopography that investigates:

- childhood residence and movement of the soldiers, and thus the recruitment areas of the army;
- childhood diet in early 17^th^ century Scotland;
- health and disease in early 17^th^ century Scotland.

### Historical context

The historical period relevant to the lives of the men found at Palace Green is the period from 1590–1650. In order to situate data gained from scientific analyses of the skeletons on their place of origin, infections, pathologies, diets and lifestyles, several aspects of life in early 17^th^ century Scotland must be considered. The range of their possible places of origin, which will be reflected in the isotopic composition of their dental enamel, may be evaluated from the recruitment areas of the Scottish army. The occupations and lifestyles of Scots from towns and countryside, and from Highlands and Lowlands, provide context for dietary, pathological and occupational inferences from dentine, calculus and bone. Accounts of famines and plagues provide context for physiological stresses identified in dentine isotopes and in bone pathologies.

### Recruitment areas of the Scottish army

In early 1650, the Scottish army was short on numbers so the threat of invasion from England prompted the Committee of Estates to order a levy of 19,000 men. This level of call-up was not achieved because 12 years of war had taken their toll on men of the appropriate age, and local committees decided that they could not supply the men demanded while maintaining essential farming and industry [13]. In some areas religious and political affiliations also reduced the turnout.

**Fig 3:**
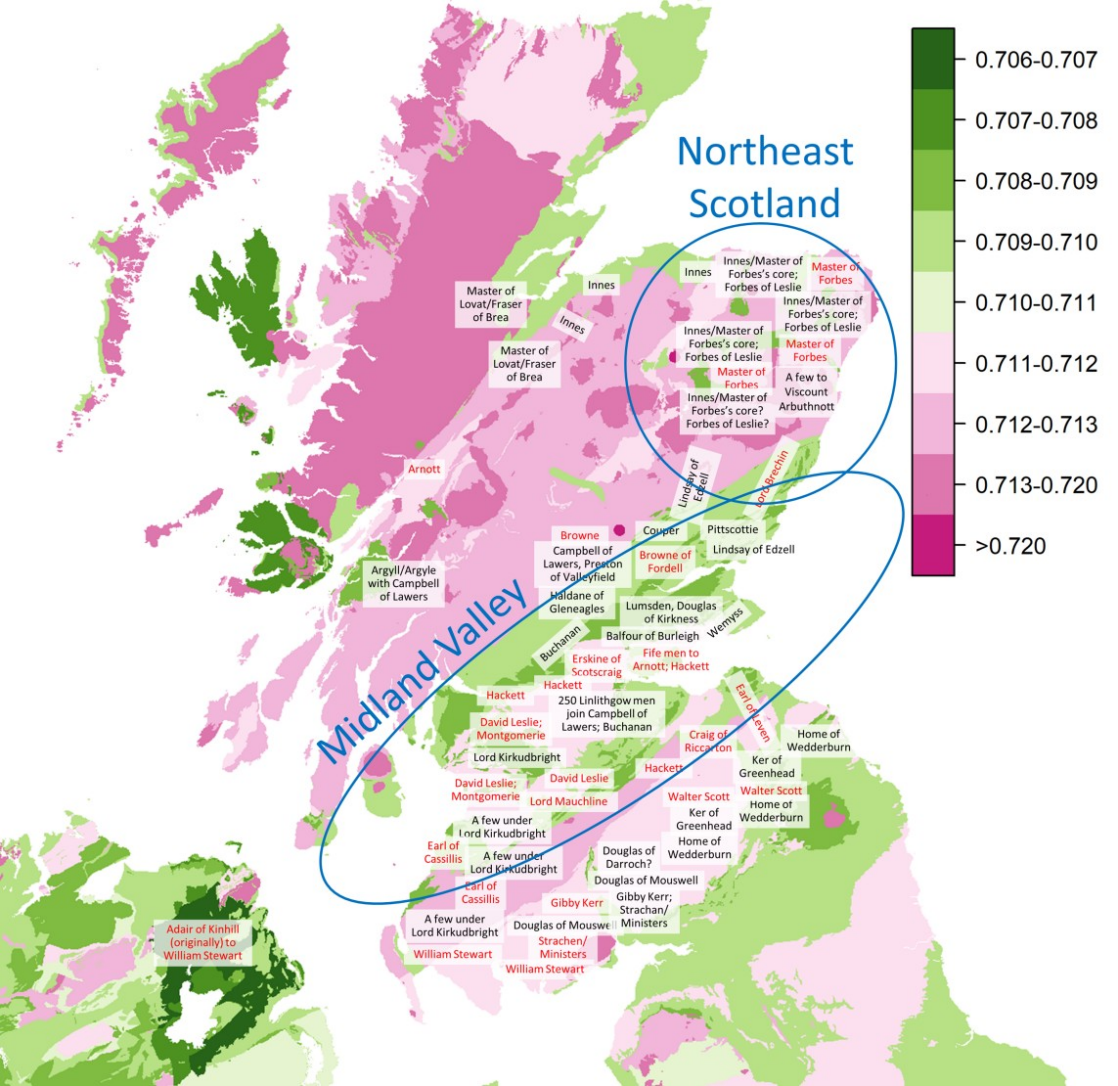
Recruitment areas for the Scottish regiments compared with strontium isoscapes. Places of origin of regiments in the Scottish army (based on [1: Fig 5.8]) overlaid on strontium isotope ratios maps of Britain (redrawn from [25]) and Ireland (redrawn from [26]). Black text indicates foot regiments and red text cavalry regiments.

The Scottish army consisted of brigades containing several regiments, primarily, but not entirely, organised geographically based on recruitment areas, and commanded by local lairds. The locations of the estates of the officers thus give an indication of the areas from which the army was raised. No comprehensive contemporary enumeration of the officers survives, but a largely complete list has been reconstructed [1: 112–116] based on previous analyses [12, 20] derived from an English intelligence survey made before the battle [21], and lists of captured officers [22] and banners [23] drawn up after the battle, supplemented by mentions in Cromwell’s letters [24], the memoirs of English Parliamentarian captain John Hodgson [22] and other contemporary sources. This allows the recruitment areas to be compared with strontium isoscapes (Fig 3). In addition, accounts of the army in the months following the battle indicate that “Dutch” and “High German” mercenaries were serving alongside the Scottish levies [20], and they are likely to have been present at Dunbar.

### Everyday life in 17^th^ century Scotland

#### Diet

From the late 16^th^ century through to the early 18^th^ century there was a steady change in diets in Scotland. Oats grew in importance, at first in the Lowlands and then in the Highlands [27]. In the first half of the 17^th^ century the Lowland diet was based on a restricted range of foods, with oats, peas, beans and kale as staples supplemented by small amounts of beef and mutton, mostly in broth [28]. The Highlands developed dependence on oats later, so in this period they ate more cheese, meat and milk than Lowlanders [28]. Although marine and freshwater fish were available, their consumption seems to have been limited [28]. These regional and temporal trends are, of course, generalisations. There were variations dependent on wealth, social rank, and location but, once these are accounted for, there was little in the way of urban-rural differences [29]. Rations for soldiers in 1639 were 2 lb (910g) of oat bread, 28 oz (790g) of wheat bread and 1 pint (570 ml) of ale per day. This allowance probably fed a soldier and his ‘follower’ [28].

#### Living conditions

Scottish living conditions in the early 17^th^ century varied widely by social status and geographic location. The majority of the population worked on the land, living in ‘fermtouns’ (farm townships). Their houses were usually wood and turf constructions of two or three rooms [30: 97]. Heated by fires but almost certainly without chimneys, these would have had smoky interiors. Urban dwellers were more likely to live in stone buildings with chimneys, but the major towns and cities, such as Edinburgh, were renowned for their smoke-filled atmosphere. Urban areas were densely occupied but lacked sanitation, leading to disposal of human waste in the streets [31], with obvious consequences for disease and health.

#### Occupations

The men recruited into the Scottish army in 1650 were likely drawn from all walks of life. As noted above the majority would have worked the land, but the Scottish economy also supported fishing communities and coal-mining [27]. In addition, we have more specific evidence for possible previous occupations of the imprisoned soldiers from accounts of the occupations in which they were later deployed. Some were sent to set up ‘Scotch-cloth’ weaving in Newcastle, implying that they knew this trade already. Prior knowledge of the work to which they were assigned is also likely for others, including those sent to work as coal miners and in salt production. It is also possible that those deported to Massachusetts who were employed as blacksmiths, in animal husbandry, as charcoal makers and as sawyers had relevant prior experience [1: ch.7].

### Epidemics and famines in early 17^th^ century Scotland

The lifetimes of the Scottish prisoners who died in Durham fell in the ‘heyday of famine and scarcity in all of Scottish history … roughly, between the middle of the sixteenth and the middle of the seventeenth century’ [29]. Most of these food shortages are indicated by legislation about prices. Few, if any of the men, were old enough to have experienced the nationwide deprivations between 1560 and 1600, but the poor harvests and famines of 1620–1625, peaking in 1623 [32], will have impacted some of them. Later shortages, notably between 1634 and 1636 in northern Scotland and 1650 in the Highlands [32] possibly affected others.

This was also a period, particularly in the 1640s, that saw repeated outbreaks of plague and a range of other diseases, including smallpox, measles, whooping cough, tuberculosis, typhoid, and dysentery [33–35]. These diseases are mostly recognised in the historical record by increased, and sometimes very high, mortality, but there would have been many others who suffered a period of illness and survived.

## Materials and methods

Twenty-eight skeletons were excavated, 18 from pit F512 and 10 from pit F514, though the formal minimum number of individuals was only 17 based on the number of right fifth metacarpals. Preservation was poor with 13 individuals less than 20% complete and none more than 80% complete. Osteological investigations yielded age and sex estimates and reported pathological lesions and traumas (Table 1) [1, 10].

The analyses reported here were mostly conducted on the dentition. Of the 28 individuals, 13 had preserved teeth. All but one of these skeletons came from pit F512 with only Sk 6 from pit F514. Details of all laboratory procedures and protocols are given in S1 Methods. The materials are no longer available for study as they were reburied in 2018.

Sk 25 and 28 were selected for microscopic examination of tooth wear because unusual wear patterns had been identified macroscopically on their anterior dentition.

For strontium and oxygen isotope investigation of mobility, permanent first, second and third molars were selected based on preservation from all 13 individuals with surviving teeth. Teeth with less wear, and which were loose or easily extracted due to prior damage to the bone, were preferentially selected. The enamel of these teeth is formed between the ages of 0 and 3 years, 3 and 8 years, and 9 and 13 years respectively [36]. The teeth were fully recorded and photographed before sampling. Sk 27A had extensive wear and lacked third molars due to ante-mortem and post-mortem tooth loss, but all the others had limited wear, no pathology and all three molars available for sampling. Using a dental burr, the surface enamel was abraded to remove any surface contamination. Approximately one third of the tooth was sectioned using a flexible diamond cutting disc and dentine was removed with a dental burr. The resulting clean core enamel was crushed and split into two aliquots, one for strontium isotope analysis and one for oxygen isotope analysis.

For incremental isotopic analysis of dentine, a permanent canine and third molar were sampled for the 12 individuals where both these teeth were available. Together their analysis can generate profiles of δ^13^C and δ^15^N, and thus a dietary history, from birth to about 23 years of age.

Twelve individuals with deposits of dental calculus were selected for microscopy and metaproteomic analyses. Samples of dental calculus were removed from the teeth using a sterile dental pick and stored in 2 ml microcentrifuge tubes.

We applied a novel biochemical approach to detect evidence for vitamin C deficiency by identifying reduced proline hydroxylation in collagen using protein mass-spectrometry [see S1 Methods]. The amino-acid composition of type I collagen, the importance of hydroxyproline in stabilizing the collagen molecule, and the role of vitamin C (ascorbic acid) in the conversion of proline to hydroxyproline are well-established [37–39]. It is, however, only recent advances in mass spectrometry that allow the identification of the location of these hydroxyprolines within the collagen molecule [40, 41]. This scurvy biomarker approach is still at an early stage of development but previous work on archaeological skeletons with evidence of scurvy, and modern guinea pigs fed on a low vitamin C diet, suggests it may be able to detect vitamin C deficiency [42, 43]. Twenty-eight samples were collected from 15 individuals, focussing on younger individuals where higher bone turnover should present the best target for detecting scurvy. Compact, trabecular and woven bone were sampled, and collagen extracted from them. Slowly remodelled compact bone should give a longer-term indication of nutritional status than trabecular bone. Woven bone from active lesions should reflect weeks or months rather than years.

All necessary permits were obtained for the described study, which complied with all relevant regulations according to English Law. Excavation was conducted under Ministry of Justice Exhumation Licence number 13–0241. Ethical approval was granted by the Ethics Committee of the Department of Archaeology at Durham University.

### Osteobiographical and prosopographical methods

To synthesise the wide range of scientific, archaeological and historical information collected by the methods outlined above, we adopt the approach of osteobiography and extend it using concepts from prosopography, an approach to the study of multiple partial biographies used by historians.

The term ‘osteobiography’ has come to have multiple, related, meanings [44, 45]. Here we adopt the approach that it is the combination of multiple lines of osteological and other analyses of a skeleton to tell the life story of an individual. The various pieces of evidence can be placed within an individual’s lifespan with greater or lesser precision, and thus ordered to give a sequence of events in the same way as a biography. With the precise dating of this skeletal assemblage to the final months of AD 1650, we can also add background from historical narratives and compare them to the reconstructed timeline of the individual’s life. However, osteobiographies are inevitably partial, and their extent is limited by the extent of preservation of the remains and the set of analyses conducted.

Prosopography, as used by historians, is likewise the subject of multiple definitions [3]. It generally involves the systematic collection of possibly incomplete biographical information about a specific group or population and analysis of that information to create a collective biography, which draws on multiple individual biographies to overcome some of the limitations of fragmentary data. It is in that sense that we use it here.

## Results

### Dental microwear

**Fig 4:**
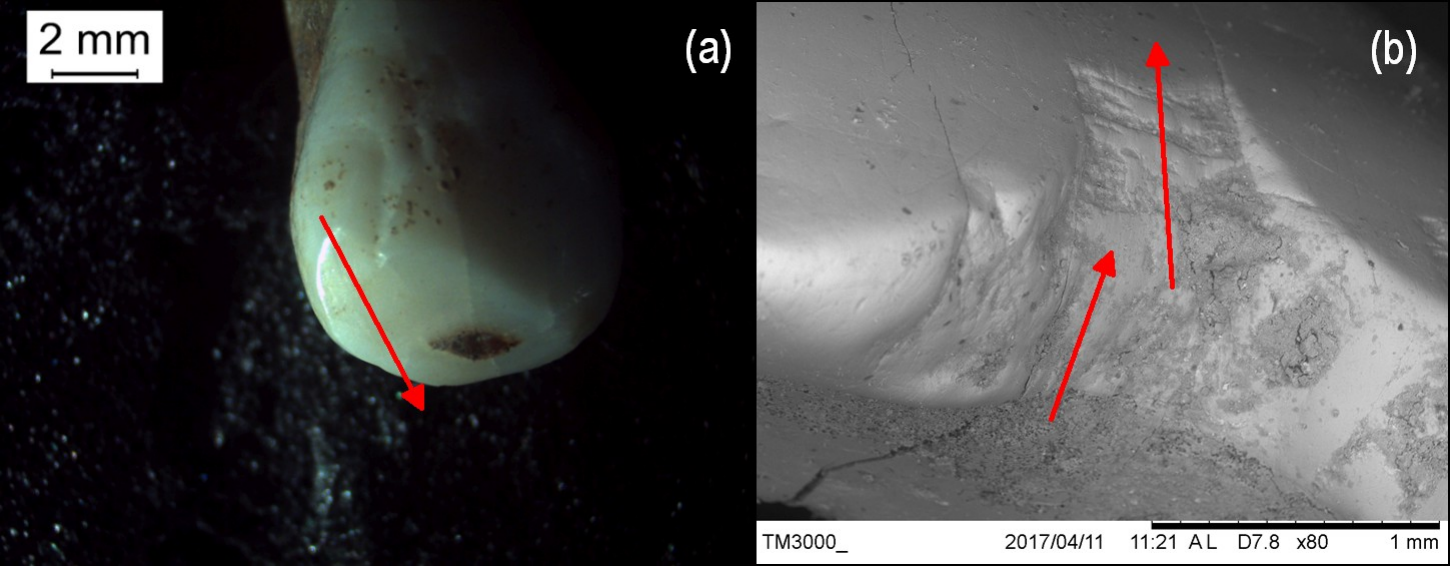
Microwear. Results of microscopic examination of tooth wear (a) Sk 25 upper right canine: the arrow indicates the direction of the striations (photo: LM); (b) SEM image of notch on the upper right first incisor of Sk 28 with arrows indicating the direction of the striations.

Sk 25 exhibited wear on the upper canines and first premolars. Examination with a dissecting light microscope showed the striae on the left canine ran in an antero-posterior direction whereas the striae on the right canine (Fig 4A) were slightly angled to the right; this may indicate tightly gripping and pulling coarse material with the right hand.

Sk 28 had a notch in the upper right first incisor and corresponding striae and gouges indicating the direction of pull of the material which was being manipulated and held between the incisors (Fig 4B). It is possible that this was caused by a narrow piece of abrasive material such as string, wool, leather thong, or a small piece of wood or metal being habitually held between the right central incisors. Heavy wear on all of the anterior dentition with pulp exposure and the young age of the individual (16–20 years old) indicate that these wear patterns are not related to mastication.

### Analysis of micro-debris in dental calculus by light microscopy

**Fig 5:**
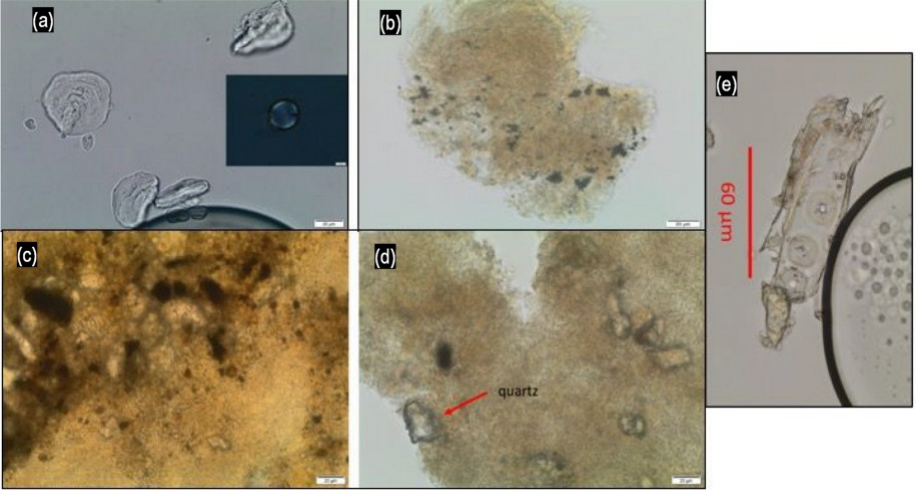
Micro-debris. Examples of micro-debris recovered from calculus. (a) damaged starch granules, (b) burnt plant matter (potentially soot) in calculus, (c and d) mineral grit in calculus, and (e) conifer wood fragment with diagnostic pits. Scale bars in (a)-(d) are 20μm.

Although in low number and frequently too minute to securely identify species, a range of microremains were found in the analysed sample (Fig 5). Four individuals (Sk 1, 23, 24, and 25) yielded plant remains consistent with starch granules from *Triticeae*, which includes wheat, barley and rye, as well as damaged starch granules of indeterminate taxon. Plant tissues resembling boiled vegetable epidermis were also detected, but taxonomic identification was not possible due to their poor preservation and lack of diagnostic features, as well as their very small size. Identifiable starch granules were mainly of dietary origin, and although the plant tissues were not identifiable, they too are likely from foods.

All 12 individuals had small particles of micro-charcoal and possibly soot in their calculus. Other micro-debris included a variety of plant and animal fibres not diagnostic to species, soil, mineral grit, and wood fragments, a few of which could be attributed to conifer wood. Some fungal spores were also observed.

There are many pathways to the inclusion of such remains into the dental calculus. Fungal spores could result from the ingestion of moulds on food, from soil or even exposure to humid environments. Wood was used in a vast number of everyday objects, and in housing, throughout history, so its presence in all the individuals’ calculus matrices is not surprising. Mineral grit, and soil too, would be common in the environment and potentially accidentally ingested during the consumption of poorly washed food such as raw vegetables. Fibres were ubiquitous but in low number and very likely belong to flax and/or hemp, as well as to wool.

The fine particles of micro-charcoal and soot could be the results of exposure to smoke (including tobacco smoking), but also roasted food, and/or a combination of both. The limited quantity of micro-charcoal identified makes the source of this micro-debris difficult to identify, but it could suggest exposure to indoor dust and particulate pollution, as does the evidence for maxillary sinusitis in eight of the individuals.

These identifications and the pathways of origin of the micro-debris must be regarded as tentative, however, due to the lack of secure identification resulting from poor preservation.

### Strontium and oxygen isotope analysis

**Fig 6:**
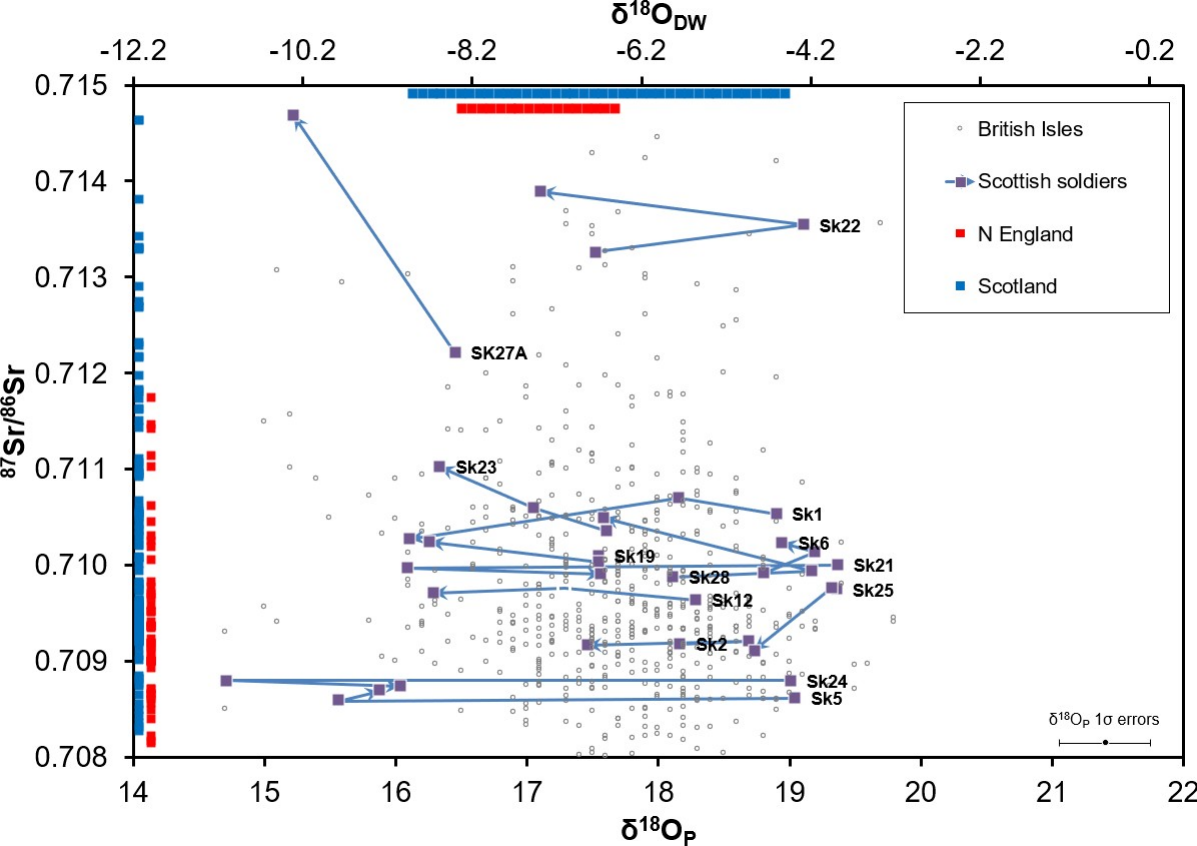
Strontium and oxygen isotope results for the Palace Green individuals. Scottish soldiers’ results (purple) overlaid on 584 published values for individuals buried in the British Isles [46] (open grey circles). Bars at the top show indicative ranges of precipitation and groundwater δ^18^O for Scotland (blue) and northern England (red), based on [49]. On the left are plotted environmental strontium isotope ratios for Scotland (blue) and northern England (red) [25]. Error bars (TEM, 1σ) for the Palace Green Library δ^18^O_P_ data are shown bottom right. Arrows run forward in time from earliest tooth to latest tooth of an individual.

The results of analysis of strontium and oxygen isotopes in dental enamel are given in S1 Table and plotted in Fig 6. Analysis of one sample failed during oxygen isotope preparation. Both strontium and oxygen show a wide range of values, spanning almost the entire range observed in a compilation of 584 previously reported individuals from the British Isles [46]. In addition, some individuals show large shifts between the values in consecutive teeth, implying movement during childhood. As noted previously with only part of this dataset [1, 47] the diverse set of ^87^Sr/^86^Sr ratios is not compatible with a common point of origin, or even a common origin in northern England, and the lowest δ^18^O_P_ are outside the expected range for the British Isles. Many of the men show only small changes in strontium isotope ratio during childhood, and in most cases the highest oxygen isotope ratio is found in the first molar, which may be an indication of the effects of breastfeeding on this early-forming tooth [48].

### Incremental dentine δ^13^C and δ^15^N analysis

**Fig 7:**
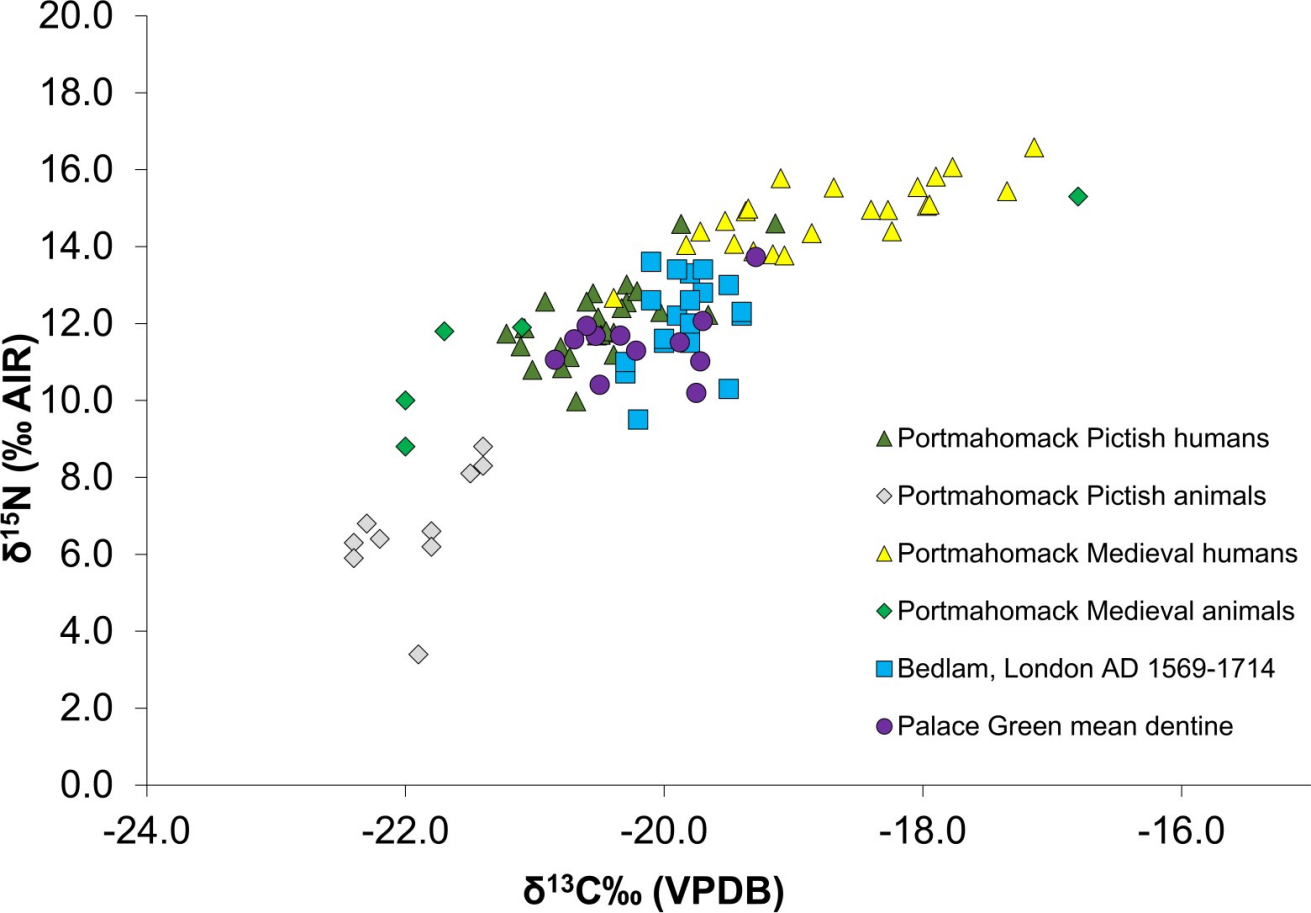
Mean dentine δ^15^N and δ^13^C for the Palace Green individuals compared to bulk bone collagen δ^15^N and δ^13^C from humans and animals from selected British sites.

The isotope ratios measured in the dentine collagen from the teeth of these individuals gave a range of 9.5 to 15‰ for δ^15^N and -21.1 to -18.0‰ for δ^13^C. The full dataset is given in S2 Table. The combined mean isotope ratios for both canine and third molar for each individual (Table 2) have been compared to contemporary populations from the 16-17th century Bedlam Hospital [50] and also to both Medieval and earlier Pictish populations from Portmahomack, Scotland [51] (Fig 7). Overall the men have predominantly C_3_ terrestrial diets with varying proportions of animal protein typical of medieval and post-medieval British diets.

**Table 2:** Mean dentine δ^15^N and δ^13^C for palace green individuals.

Sk number	δ^15^N ‰	δ^13^C ‰
1	11.0	-19.7
2	13.7	-19.3
5	12.1	-19.7
6	11.3	-20.2
12	11.5	-19.9
19	11.1	-20.8
21	11.7	-20.3
22	11.7	-20.5
23	10.4	-20.5
24	10.2	-19.8
25	11.9	-20.6
28	11.6	-20.7

**Fig 8:**
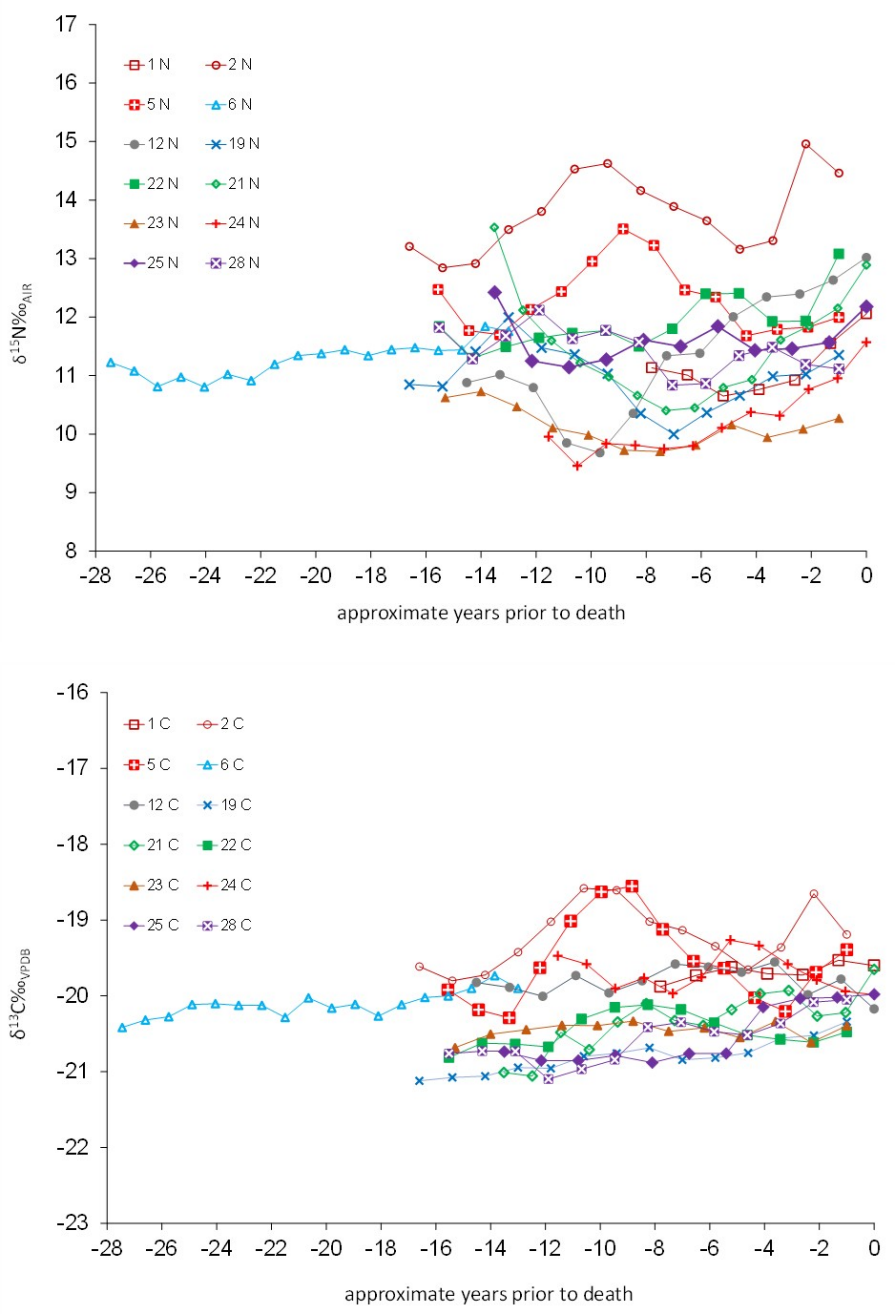
M3 isotope profiles by approximate age of formation (a) δ^15^N, (b) δ^13^C.

Isotope profiles for each individual plot the isotope data against the age at which each section formed (Figs 8 and S1). All the individuals appear to have been breastfed with weaning underway by nine months of age. The flat profile (after weaning) of the oldest individual investigated (Sk 6) suggests a consistent diet with no major physiological insults. This contrasts to the other 11 individuals who all show perturbations that indicate periods of marine protein consumption, shifts to higher or lower meat consumption, or periods of severe dietary or health stress. Of particular note, is that the three individuals (Sk 1, 24, 25) who died whilst their teeth were still forming, and others who died shortly after the tooth completed (Sk 12, 22), show a rise in δ^15^N greater than analytical error (±0.2‰) combined with flat or falling δ^13^C towards the end of life. This suggests they all experienced a period of nutritional or health stress, whilst others with covarying δ^15^N and δ^13^C (Sk 2, 6, 19 and 21) experienced a dietary change towards the end of the formation of their third molars [52].

Two pairs of profiles are very similar. Sk 2 and 5 show evidence for a shift to increased marine protein consumption between c. 12–20 years of age, returning to more terrestrial values before completion of the M3 root. The second pair, Sk 12 and 24 show declining δ^15^N from about 4 years until about the age of 10–12 years, suggesting that at those ages their diet was low in animal protein, changing to a more mixed diet at by 14–16 years of age. These shared dietary histories possibly relate to shared changes in residence or a response to environmental or political change.

### Metaproteomic analysis of dental calculus

**Fig 9:**
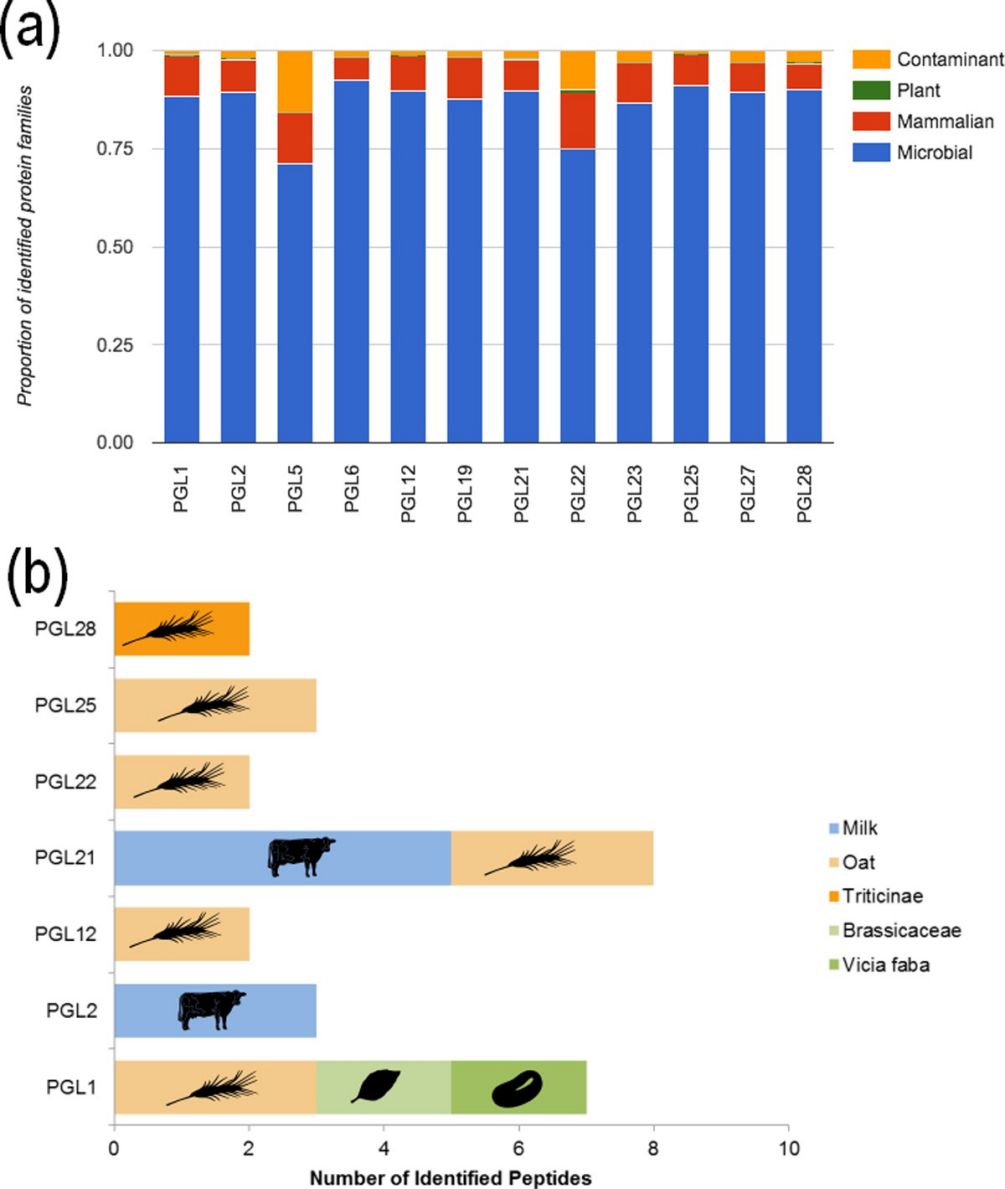
The taxonomic composition of the proteins detected in dental calculus. (a) Proportions by broad taxonomic categories, (b) identified peptides of likely dietary origin.

The number of identified proteins per individual ranged from 47 to 1059 (S3 Table), but did not correlate with the mass of calculus subsampled for protein extraction. As noted in previous analyses of dental calculus [53], microbial proteins were the dominant taxonomic group, followed by mammalian proteins (principally human proteins) with far fewer proteins assigned to putative dietary plant sources (Fig 9).

Potential dietary proteins were identified in seven individuals (S4 Table). Sk 2 and 21 displayed evidence of beta-lactoglobulin, a protein found within the whey fraction of milk, with peptides that could be assigned to the subfamily Bovinae, which includes domestic cattle. Oat (*Avena sativa*) seed storage proteins were found in five individuals (Sk 1, 12, 21, 22 and 25). An alpha-amylase inhibitor assigned to the Triticinae tribe (including *Triticum* and *Aegilops*) was observed in Sk 28. Two other putative plant proteins were identified at lower confidence in Sk 1, which were assigned to fava beans (*Vicia faba*) and Brassicaceae (patatin like protein 7). These latter two identifications are less robust as only two peptides matching to the same region of the protein were detected.

Over 60 human proteins were identified within the dataset. As with proteomic analysis of medieval calculus [54], immune system proteins, such as immunoglobulins, were detected, as was alpha-amylase, a salivary digestive enzyme. The STRING resource [55], was used to investigate the functional interactions between identified proteins, and to elucidate the participation of these proteins in biological processes. As a whole, the human proteins demonstrate significant enrichment in several biological processes. At least 80% of proteins (n = 48) were functionally connected to at least one other protein in the network, with significant enrichment in 105 biological processes.

**Fig 10:**
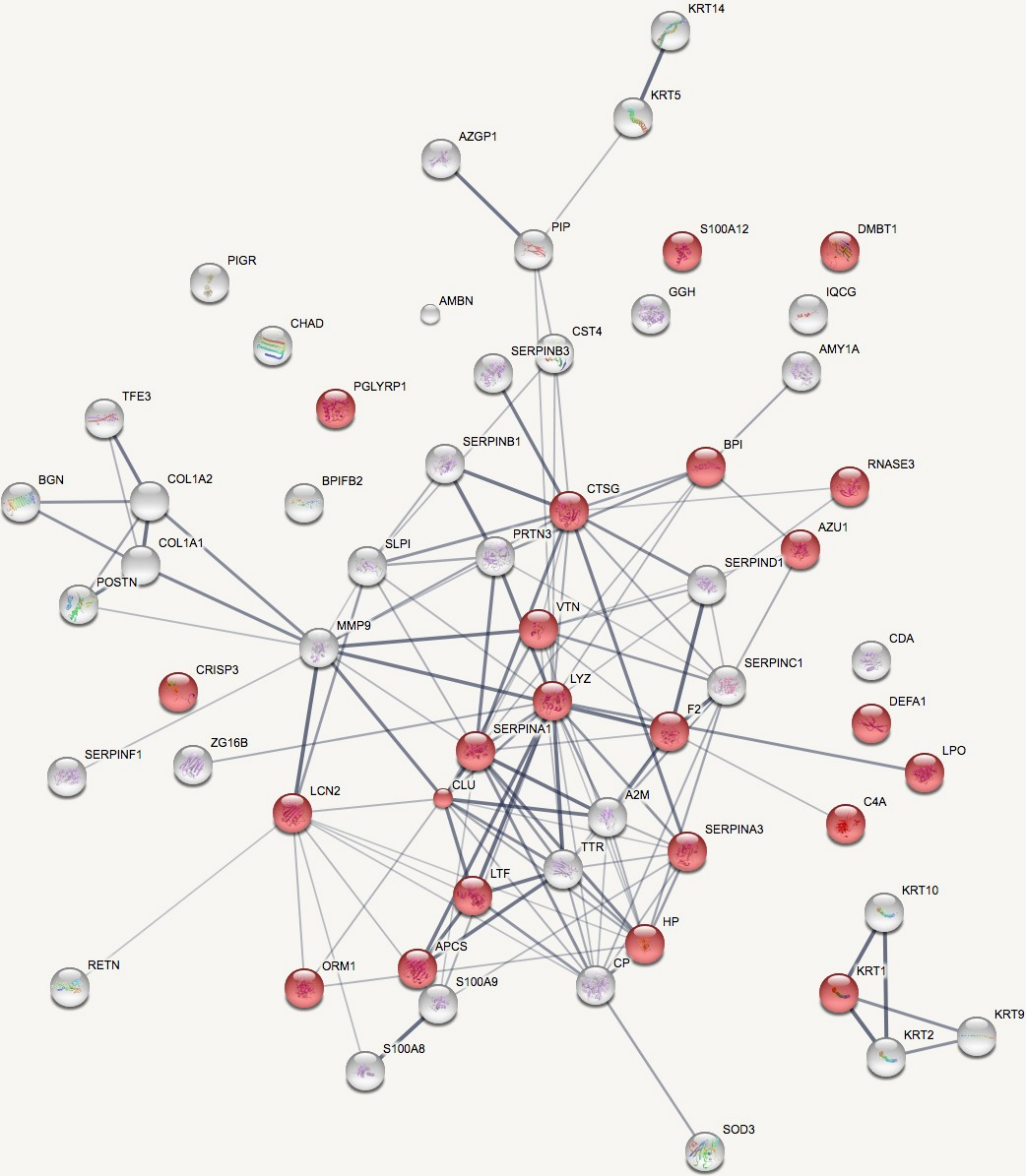
STRING network representation of human proteins identified in calculus. Red nodes are proteins significantly enriched in the biological process of ‘Defence response to bacteria’ (nodes are labelled by protein name).

The majority of human proteins are associated with the innate immune system, and are significantly enriched in biological processes related to defence against bacteria, inflammation, innate immunity and host defence (Fig 10). This is to be expected from a host reaction to a bacterial biofilm and plaque build-up. Structural proteins, (collagens and keratins) were also observed, but are assumed to result from contamination with shed skin cells during handling of the remains, though they may reflect endogenous host proteins, incorporated into the calculus in vivo through their presence in gingival crevicular fluid [56–58].

**Fig 11:**
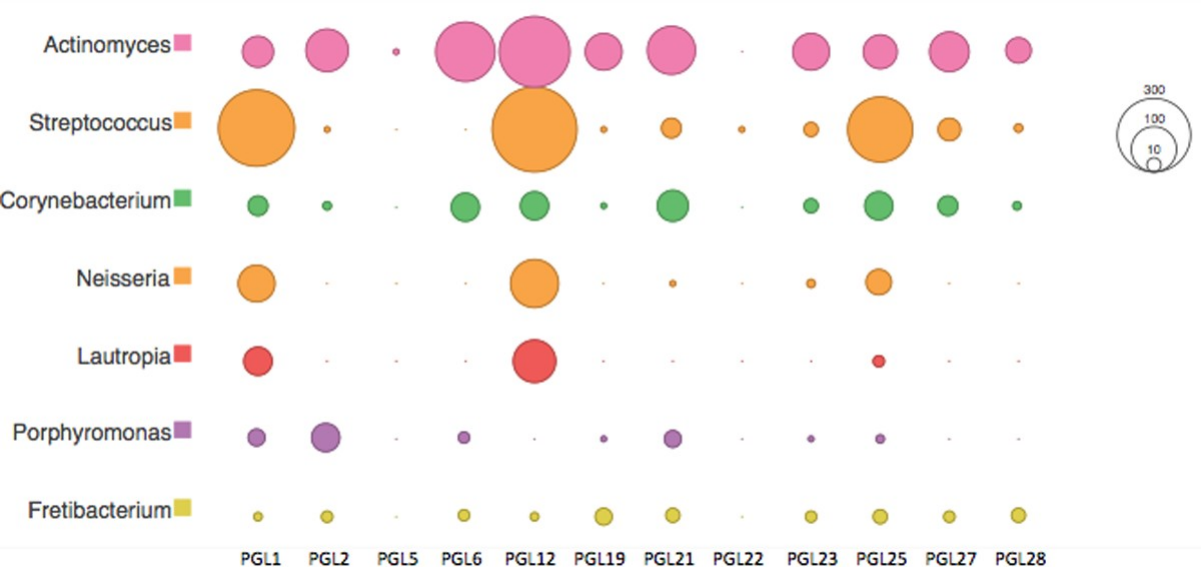
Bacterial genera with the highest frequency of identified peptides within the calculus (square-root scale).

The microbial proteins identified were dominated by bacteria, with few proteins that could be confidently assigned to viruses or archaea. These included commensal and plaque-forming bacteria typically found within the oral cavity, as well as pathogenic species, and environmental species that may result from laboratory or soil contamination. The most prevalent oral genera included *Actinomyces*, *Streptococcus*, *Corynebacterium*, and *Lautropia* (Fig 11).

**Fig 12:**
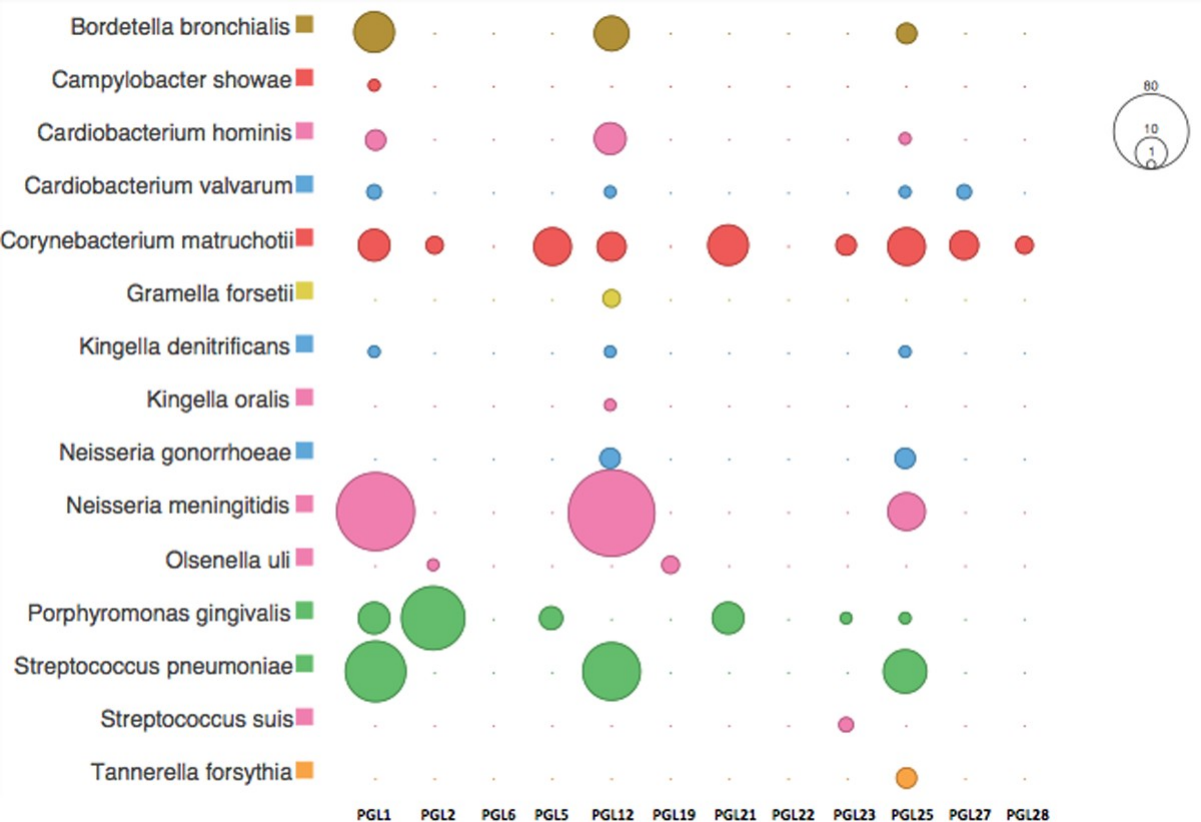
Peptide abundance for selected periodontal and opportunistic pathogens within the calculus (square-root scale).

The healthy human oral microbiome includes a number of pathogenic species, which may or may not be actively involved with oral or systemic disease progression. The most commonly identified pathogenic organism in this dataset, *Porphyromonas gingivalis*, is associated with the progression of periodontal disease (Fig 12). *P*. *gingivalis* is a member of the so-called ‘red complex’ including *Tannerella forsythia* and *Treponema denticola* [59], the former of which was also confidently identified in one individual (Sk 25).

Opportunistic oral pathogens such as *Olsenella uli*, *Corynebacterium matruchotii*, *Kingella denitrificans*, and *Campylobacter showae* were also detected. These species are commonly observed within healthy oral microflora, but can cause endodontic or other localised infections in immunocompromised individuals, and may be more prevalent in the plaque biofilms of individuals with periodontitis compared with healthy individuals [60]. Likewise, the oral taxa of *Cardiobacterium hominis* and *Cardiobacterium valvarum*, opportunistic pathogens implicated in endocarditis [61], were also detected in low abundance. *Neisseria meningitidis* and *Neisseria gonorrhoeae*, the causative agents of bacterial meningitis and gonorrhoea, respectively, were confidently identified in three and two individuals, respectively. Both of these *Neisseria* species are obligate human taxa and common members of the human oral microbiome. Opportunistic pathogens implicated in upper and lower bacterial respiratory infections, such as *Streptococcus pneumoniae* and *Bordetella bronchialis* were also identified. No conclusive proteomic evidence of epidemic pathogens, such as *Yersinia pestis* (bubonic plague), *Shigella dysenteriae* (dysentery), *Mycobacterium tuberculosis* complex (tuberculosis), or *Treponema pallidum* (syphilis) was identified within the dental calculus datasets. Nevertheless, the extent to which dental calculus entraps and preserves transient epidemic pathogens is unknown. Preservation may be unlikely in cases where the time between infection and death is very short, given that dental calculus may not significantly accumulate during this period.

### Scurvy biomarker analysis

All of the bones analysed yielded sufficient collagen for proteomic analysis. Only one sample of the 28 failed to provide sufficient quality collagen spectra. PGHK19 (Sk 25 woven bone from the mandible) was therefore omitted from further analyses. The quality and quantity of the MS/MS data are indicated in Table 3 by the number of unique queries and number of collagen type I peptides identified for each sample, after filtering with an ion score cut-off of 20 and a false discovery rate of 1%. The mass spectrometric analysis provided excellent sequence coverage of the collagen molecule (approx. 80–90%) and consequently all 12 potential scurvy biomarker peptides were observable in each of the remaining 27 samples.

**Table 3:** Samples and results for scurvy biomarker analyses.

Sk no.	Bone Type	Skeletal element	Bradford lab identifier	No. of queries	Identified COL1a1 and COL1a2 peptides	Under-hydroxylated peptides
1	Compact	Mandible	PGHK1	17031	1120	12
1	Trabecular	Rib	PGHK14	16166	998	6
1	Woven	Ulna	PGHK15	15319	1006	4
2	Compact	Mandible	PGHK2	14894	887	10
5	Compact	Mandible	PGHK3	16458	953	7
5	Trabecular	Rib	PGHK26	14993	766	6
6	Compact	Mandible	PGHK13	15556	794	12
6	Trabecular	Rib	PGHK16	15822	831	7
8	Compact	Midshaft femur	PGHK21	13588	721	7
8	Trabecular	Calcaneus	PGHK25	11991	560	3
8	Woven	First metatarsal	PGHK22	13746	560	3
12	Compact	Mandible	PGHK4	16469	998	10
12	Trabecular	Rib	PGHK27	14124	690	4
14	Compact	Midshaft tibia	PGHK23	13957	561	4
14	Woven	Tibia	PGHK24	15374	837	8
19	Compact	Mandible	PGHK5	15880	911	8
21	Compact	Mandible	PGHK6	15834	871	7
22	Compact	Mandible	PGHK7	16635	1044	9
22	Trabecular	Rib	PGHK28	17275	884	7
23	Compact	Mandible	PGHK8	17383	979	7
23	Trabecular	Rib	PGHK18	16925	944	0
23	Woven	Mandible	PGHK17	9392	372	3
24	Compact	Mandible	PGHK9	16372	961	7
25	Compact	Mandible	PGHK10	17347	980	9
25	Woven	Mandible	PGHK19	4906	50	no data
27A	Compact	Mandible	PGHK11	13919	753	7
27A	Trabecular	Rib	PGHK20	15069	898	8
28	Compact	Mandible	PGHK12	18521	1064	8

**Fig 13:**
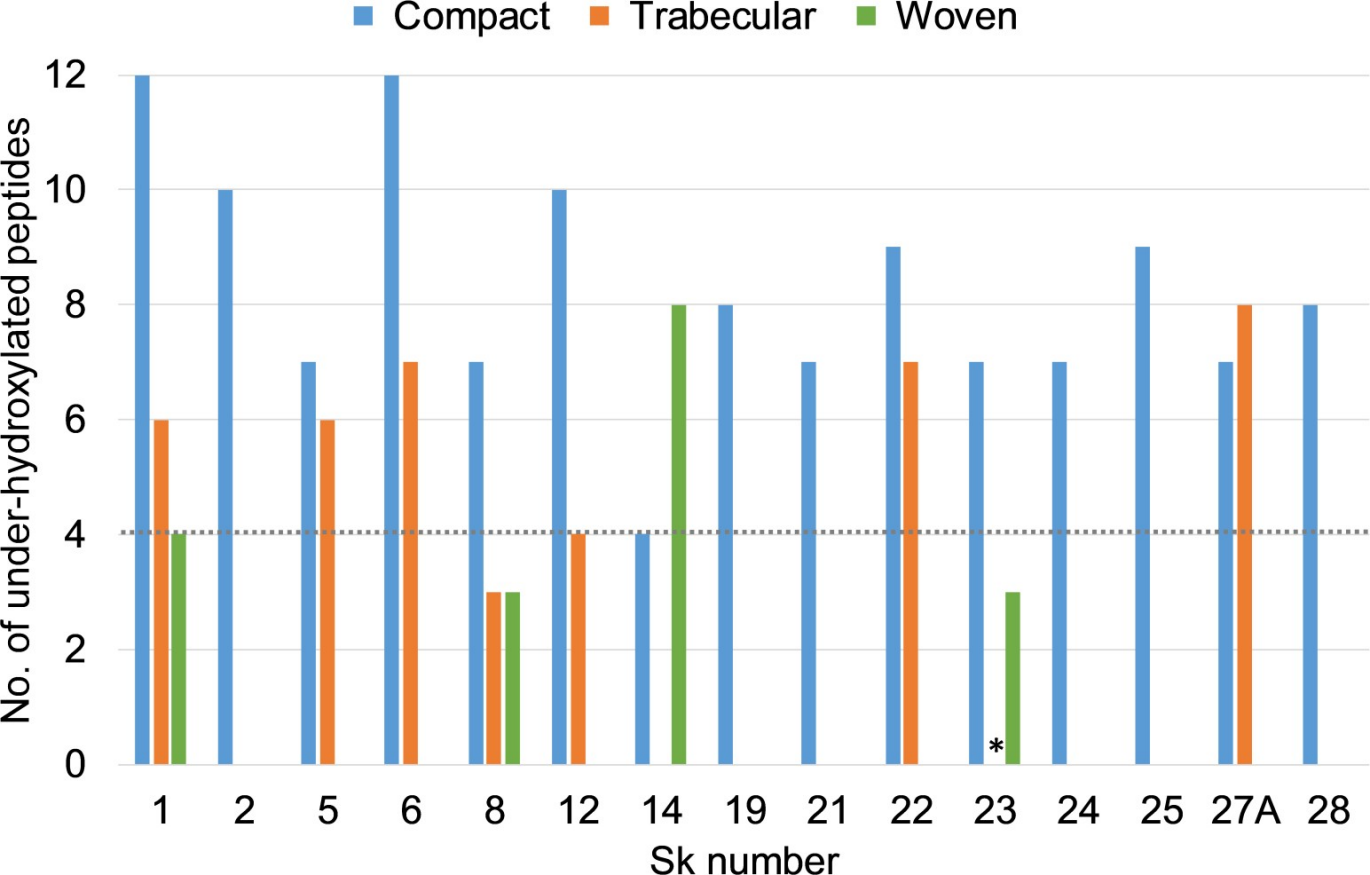
Numbers of collagen peptides containing the under-hydroxylated variant. Where no bar is shown, no sample was taken, except where marked *. Dotted line: upper limit of 4 under-hydroxylated peptides observed in non-scorbutic individuals.

Fig 13 shows the frequency of under-hydroxylated biomarker peptides in compact-bone collagen from the mandible (except for Sk 8, mid-shaft femur and Sk 14, mid-shaft tibia). In healthy, non-scorbutic individuals with sufficient dietary vitamin C the under-hydroxylated variant peptides occur at a frequency of 0–4 [S1 Methods]. All samples except Sk 14 exceed this, suggesting a period of vitamin C deficiency at some point in their lives during which the compact bone was being laid down.

Trabecular (predominantly rib, except Sk 8, calcaneus) and woven bone samples with shorter remodelling timescales are compared to compact bone in Fig 13. Hydroxylation levels consistent with normal ‘healthy’ collagen are observed in a rib from Sk 12, a calcaneus from Sk 8, and woven bone from Sk 1, 8 and 23. In the majority of cases, higher levels of the under-hydroxylated variant are observed in the slower-turnover compact bone rather than trabecular bone. This suggests that these individuals may have experienced a long-term vitamin C deficiency rather than from an acute period of deficiency towards the end of their life.

The interesting differences observed between and within individuals should be interpreted cautiously. As noted above this biomolecular approach is still in a preliminary stage of development. Although it is likely that variations seen are the result of different levels of vitamin C, without large-scale studies we cannot rule out variations between different skeletal elements or different types of bone, inter-individual metabolic differences, or interactions with infections.

The limited set of results from woven bone are intriguing. With active scurvy, the new bone laid down just prior to death would be expected to exhibit the most pathological collagen, whereas the opposite appears to be the case here. In three out of four individuals woven bone showed the fewest markers, at a level consistent with non-scorbutic bone collagen. This might be explained by an aetiology unrelated to diet, for instance an inflammatory response to a localized infection. Alternatively, if an individual is severely malnourished and vitamin C deficient, they may not be able to produce new collagen, and the laying down of new woven bone could indicate a period of recovery with sufficient dietary vitamin C. Unfortunately, to date there is a lack of research on changes in bone physiology and chemical composition during growth and remodelling in periods of significant nutritional stress.

## Discussion

According to Verboven et al. [62], historical prosopography proceeds by defining the group or population to be studied, and establishing a ‘common questionnaire’ of biographical facts to be sought about the individuals in the group, though data collection may well be incomplete for some individuals. A good prosopography then utilises background information about the context in which the individuals operated, and a synthesis of results to attempt to explain them in the light of specific research questions. Our investigation of the skeletons from Palace Green conforms to this rubric. The group is defined by their common burial, and their shared experience as soldiers in the Scottish army. The multiple lines of evidence produced by a wide range of scientific methods differs in extent for each individual due to the vagaries of survival of skeletal parts and varying availability of molecular evidence (Table 1). To accommodate this, we build an osteobiography for each individual using the partial evidence for that man, and then synthesise this using the prosopographical approach described above.

### Osteobiographies

In this section we present an integration of the data for each skeleton to create an osteobiography for every man in order to understand his life course. The evidence presented above in the results section is referred to using the following abbreviations:

- SrO: strontium and/or oxygen isotope analysis
- Os: osteological evidence
- De: incremental dentine δ^13^C and δ^15^N
- We: dental microwear
- Mi: microscopic analysis of dental calculus
- Pr: proteomic analysis of dental calculus
- Sc: scurvy biomarker analysis

#### Skeleton 1

Born in 1634–1636 [Os], Sk 1 probably grew up in the Midland Valley of Scotland, though other locations scattered around the Highlands are possible [SrO]. He was breastfed during infancy [De]. His oxygen isotopes suggest he may have moved eastwards during childhood, but only limited areas are compatible with his strontium isotopes [SrO]. During that time, he had rickets and multiple episodes of stress from diet or disease shown in enamel hypoplasias [Os] including shifts from his normal diet [De]; his narrow palate could indicate malnutrition [Os]. During the formation of his mandible he may have suffered badly from scurvy [Sc]. Plant remains in his dental calculus suggest the consumption of oats, beans, and brassicas [Pr]. He may have had lower back pain, caused by an extra rib on his right side at the bottom of his ribcage, and a transitional vertebra at the lumbar to sacral border; the latter may also have caused sciatica [Os]. Sk 1 may have suffered further pain from a medium-sized cavity in a lower right molar and had generally poor dental health [Os]. His sinusitis [Os] may be linked to inhalation of micro-charcoal and soot from smoke [Mi], but also to the presence of bacteria linked to respiratory infections, *Streptococcus pneumoniae* and *Bordetella bronchialis*, in his calculus [Pr]. Long ago he had inflammation in both femora, which could have been caused by an infection or scurvy, but that had healed [Os]. Several chips in his upper incisors suggest he may have habitually used his teeth as tool [Os]. At the time of his death he had inflammation of his left ulna [Os], which could have been caused by infection but seems not to have been due to scurvy [Sc]. Rising δ^15^N combined with flat δ^13^C suggests nutritional or health stress in the last 2½ years of his life [De] [52]. He died aged 14–15½ [Os].

#### Skeleton 2

Skeleton 2 was born between 1625 and 1632 [Os], probably on the northern or southern edges of the Midland valley, or in Berwickshire [SrO]. There is no evidence that he moved location during childhood [SrO]. During infancy he was breastfed but also experienced significant health stress [De]. His childhood diet differed from other men in the group, but fits with other contemporary Scottish groups [De] (Fig 7). At the ages of 11–13 he experienced a period of marine protein consumption (probably fish), shifts to higher or lower meat consumption, or periods of severe dietary or health stress [De]. These may also have contributed to his cribra orbitalia [Os], and may be associated with severe scurvy during the formation of his mandible [Sc]. He also had sinusitis [Os], which may have been caused by persistent exposure to smoke, as his teeth showed possible signs of pipe-smoking [Os] and micro-charcoal and soot were found in calculus on his teeth [Mi]. His dentition included an additional incisor, but this probably had little effect on him [Os]. Periodontal disease [Os] and the presence of *Porphyromonas gingivalis*, an organism associated with periodontal disease [Pr], together with calculus [Os] indicate that he had poor oral hygiene [Os]. Proteins in his calculus indicate that he consumed dairy products [Pr]. He died aged 18–25 [Os].

#### Skeleton 3

Skeleton 3 was born between 1627 and 1633 [Os]. At some time he had strained his back causing injuries to his intervertebral discs [Os]. He had healed inflammation on both femora, most likely due to an infection [Os]. He died aged 17–23 [Os].

#### Skeleton 4

Skeleton 4, of unknown sex, was born in 1632–34 [Os]. At death Sk 4 was experiencing inflammation in both legs and left foot, probably caused by an infection [Os]. Skeleton 4died aged 16–18 [Os].

#### Skeleton 5

Skeleton 5 was born between 1627 and 1633 [Os], and spent his childhood outside the British Isles, at least after the age of 3 [SrO]. He was breastfed during infancy [De]. His oxygen isotopes indicate significant movement between the ages of 0–3 and 3–13 years, though remaining on geological formations of similar strontium isotope composition. This could indicate early years in Scotland followed by time abroad in Scandinavia or eastern Europe, but breastfeeding causing elevation of the δ^18^O of his M1 could account for some of this variation [SrO]. During his teenage years (aged 14–16) he experienced periods of marine protein consumption (probably fish), shifts to higher or lower meat consumption, or periods of severe dietary or health stress [De]. His teeth show chipping and unusual wear, indicating that he probably used his teeth as a ‘third hand’ in his occupation [Os]. He had strained his back, causing injuries to the intervertebral discs (Schmorl’s nodes), and had an extra segment in his sacrum, but these conditions were probably asymptomatic [Os]. He died aged 17–23 [Os].

#### Skeleton 6

Skeleton 6 is one of the older men studied. Born before 1604 [Os], he may have spent his childhood in the western part of the Midland Valley, or further north in Caithness or the Orkney archipelago [SrO]. During childhood he only moved short distances, if at all [SrO]. He was breastfed during infancy, but after this, up to 23 years of age, his flat δ^13^C and δ^15^N profile suggests a consistent diet of mixed terrestrial animal and plant proteins with no major physiological stress [De], which stands in contrast to all the other men. Although he seems to have been sufficiently well-nourished and in a reasonable state of health with no sign of childhood stresses from disease or diet [De], he only grew to a close-to-average height of 166 cm [Os]. After childhood, Sk 6 had a tough physical life. Well before he fought at Dunbar, he strained his back causing Schmorl’s nodes [Os], but he was probably unaware of his injury, which would not necessarily have caused ongoing pain [63]. He also had a healed injury to the soft-tissue at the back of his neck, and may have had further healed injuries to his head, though they could have been the remnants of cysts [Os]. He may have used his teeth as a tool causing chipping of his upper incisor [Os]. As with any man of his age, he had developing joint degeneration with mild osteoarthritis in his upper body and hip. Changes to his spine (S1D Methods) may be due to trauma but could also indicate the early stages of the disease DISH (diffuse idiopathic skeletal hyperostosis), which can be associated with obesity, Type 2 diabetes, and a rich diet [Os]. He had probably experienced a long-term vitamin C deficiency which had lessened in last few years of his life [Sc]. His age and injuries are consistent with him being a seasoned campaigner. He died aged over 45 [Os].

#### Skeleton 7

Born in 1631–1634 and of unknown sex, Sk 7 may have had rickets during childhood [Os]. An area of healed inflammation on the left femur was may have been due to an infection or some other cause [Os]. He died aged 16–19 [Os].

#### Skeleton 8/16A

Skeleton 8/16A, of unknown sex, was born in 1635–1637 [Os]. An inflammation of unknown cause was active in his left thigh, right tibia and left foot at the time of his death [Os]. He died aged 13–15 [Os].

#### Skeleton 9

Represented by only a few bones, he was born in 1632–1634 and died aged 16–18 [Os].

#### Skeleton 10/11

Skeleton 10/11 was born in 1632–1634, and may have had rickets at an age when he was crawling [Os]. He died aged 16–18 [Os].

#### Skeleton 12

Skeleton 12 was born between 1623 and 1634 [Os], and probably spent his childhood in one place, which could have been in the Midland Valley, south-east Scotland, on the shores of the Moray Firth, or further north in Caithness or the Orkney archipelago [SrO]. He was breastfed during infancy but despite experiencing poor nutrition or disease in childhood [De], he grew to almost average height at 167 cm [Os]. His diet contained a mix of protein sources but was low in animal protein from about ages 4–12 [De], and included oats which left traces in his calculus [Pr]. Like Sk 6, he probably had a long-term vitamin C deficiency, a condition that had lessened in last few years of his life [Sc], and might be associated with the other evidence for poor nutrition. Sk 12 had poor dental hygiene and could have suffered pain from small cavities in three of his teeth [Os]. He had an inflammation leading to new bone formation inside the cranium, although there are multiple aetiologies for this change [64], it may have been associated with the presence of *Streptococcus pneumoniae* and *Neisseria meningitides* in his calculus as both organisms are associated with meningitis. His sinusitis was perhaps a result of habitual pipe-smoking [Os] and related to the presence in his calculus of organisms linked to respiratory infections, *Streptococcus pneumoniae* and *Bordetella bronchialis* [Pr]. His was a tough physical life, leading to spinal strain (S1E Methods), a possible fractured and healed rib, and a groove in a lower front tooth that might be from habitual activity [Os]. He experienced nutritional or health stress for many months towards the end of his life [De]. He died aged 17–23 [Os].

#### Skeleton 13

Skeleton 13 was born between 1625 and 1632 [Os]. He had pilasterism on his right femur which may be related to having had rickets as a child [Os]. He died aged 18–25 [Os].

#### Skeleton 14

This individual of unknown sex was born in 1634–1638 [Os]. Only the lower right leg and foot survived. All bones were light and fragile, with porous trabecular bone in the medullary cavities of the long bones, and inflammation of both the leg and foot [Os]. These lesions are likely to have been caused by a systemic condition, which may well have been a metabolic disorder, and active rickets or scurvy [Sc] at the time of death is a possibility. He died aged 12–16 [Os].

#### Skeleton 15

Represented by only a few bones, this man was born about 1630–1634 and died aged 18–25 [Os].

#### Skeleton 16C

Only the foot bones of this adult individual were recovered. Sk 16C was born before 1632 and died aged over 18 [Os].

#### Skeleton 17/16B

This individual of unknown sex was born before 1632 [Os]. Sk 17 had a healed infection of both tibiae, and had possibly had a soft-tissue injury to the left foot [Os]. Sk 17 died aged over 18 [Os].

#### Skeleton 18

Skeleton 18 was represented by only a few bones. He was born 1627–1633 and died aged 17–23 [Os].

#### Skeleton 19

A man of just above average height (174 cm), Skeleton 19 was born in the period 1625–1632 [Os]. His strontium and oxygen isotopes are very uniform and indicate no movement during childhood [SrO], which may have been spent in the north of the centre of the Midland Valley or the east coast of Scotland near Dunbar or Aberdeen. He was breastfed during infancy but during childhood he experienced periods of inadequate nutrition or disease leading to repeated enamel hypoplasia [Os] (S1F Methods) and shifts in the isotope composition of his dentine [De]. His teeth were slightly crowded and some were rotated from their normal positions [Os]. He experienced severe dental infections in the lower left jaw, evidenced as caries and an abscess (S1G Methods). These would likely have made chewing painful and could have led him to chew exclusively on the right side [Os]. This may have contributed to the uneven wear on his teeth [Os], but chipping in his upper incisor may also indicate the use of his teeth as a tool [Os]. The calculus on his teeth had trapped micro-charcoal and soot, potentially indicating that he was exposed to smoke, although other sources for such inclusions are possible [Mi]. He died aged 18–25 [Os].

#### Skeleton 20

Skeleton 20 was born before 1632 and died aged over 18 [Os]. As only their leg and foot bones were recovered, their sex could not be estimated [Os].

#### Skeleton 21

This man was born around 1625–1632 [Os] most likely in the west of the Midland Valley. He moved further east during middle childhood, most likely to the region between Aberdeen and Falkirk, but areas of south-east Scotland, and the shores of the Moray Firth are also possibilities [SrO]. He was breastfed during infancy [De] and experienced some dietary or disease stress which is visible isotopically [De] but it was not of the type or severity to cause disruption to the formation of his tooth enamel [Os]. Sk 21’s diet included milk and oats [Pr], and consumption of the latter suggests a Lowland origin rather than a Highland one, consistent with the evidence for childhood location [SrO]. He habitually smoked a pipe [Os] (S1H Methods) and possibly spent time in smoky environments [Mi], and either of these may have contributed to the development of his sinusitis [Os]. The presence of *Porphyromonas gingivalis* may indicate periodontal disease [Pr] but this had not affected the bone [Os]. He probably had a tough physical life as he showed evidence for spinal strain and also inflammation on the internal surface of the cranium [Os] of uncertain cause [64]. Damage to his cranium was probably occurred after he died, when the grave was disturbed by later activity [Os]. He died aged 18–25 [Os].

#### Skeleton 22

The man represented by Sk 22 was born in 1625–1632 [Os]. The first three years of his life were possibly spent in the western Highlands, or in south-west Scotland [SrO]. He was breastfed with weaning complete before about 2 years of age. The formation of his tooth enamel was disrupted, leaving signs of enamel hypoplasia on his premolars [Os]; this corresponds to a period of insufficient nutrition at ages 5–7 years [De], but may also have been due to childhood disease. He may have been anaemic which caused his bone marrow to expand and left porosity in the bone of his orbits [Os]. Such anaemia has various potential causes including dietary deficiencies and chronic infection [65]. He probably lived at a different location in the western half of Scotland during his middle childhood, in the 1630s [SrO]. In later childhood he moved once again [SrO]. While his teeth developed relatively normally, several were chipped and the rotation of his upper right second molar may have helped to trap food between the teeth and predisposed the first and second molars to decay and caries [Os]. Lacking oral hygiene, the plaque on his teeth calcified to form thick calculus, and he developed several cavities and abscesses which are likely to have been painful [Os] (S1I Methods). His lower left second molar had fractured [Os]. His diet, at least in the last few years of his life, included oats [Pr].

During his adolescent or early adult life in the 1640s, he herniated a vertebral disc in the middle of his back [Os]. This was a relatively minor problem and he was probably unaware of it [63]. He probably experienced a slight lack of vitamin C over the last few years of his life, but not earlier [Sc]. The protein in his diet was a mixture of plant and meat protein with no large component of marine fish [De]. Around the ages of 15–17 and 21 years he experienced further periods of insufficient nutrition [De]. Several months or more before his death, he was cut by a blade above his left eye; the incision was deep enough to mark the bone of his forehead [Os] [S1J Methods]. He might have received this wound during a fight, but an occupational injury is also possible. The cut in the bone healed well [Os], but the scar that it left behind in the overlying skin would have been quite noticeable. The microscopic pieces of charred plant matter encapsulated within his calculus [Mi] might be a residue from smoking a pipe, though he did not habitually clench a pipe in his teeth as some of his companions did [Os]. Alternatively, he might have been exposed to poor air quality (pollution) in a house with an open wood or peat fire. He died aged 18–25 [Os].

#### Skeleton 23

Despite his youth, Sk 23 had experienced a long history of physical injuries and disease since his birth in 1631–1633 [Os]. He had moved within a limited set of regions in his early and middle childhood, which was probably spent in the north of the Midland Valley or Aberdeenshire, though scattered locations in the Highlands are also possible. He moved in later childhood though not necessarily far, as other places in the Midland Valley and Aberdeenshire, as well as central southern Scotland, are possible locations [SrO]. Although he was breastfed, he experienced multiple stress episodes in infancy and childhood, exhibited in severe enamel hypoplasia [Os] and in the isotopes of his tooth dentine [De]. This stress also included vitamin D deficiency leading to rickets and causing bowing of his arms from when he was crawling and later his legs when he was able to walk [Os]. He also probably experienced long-term vitamin C deficiency, though this had lessened in the years before his death [Sc]. This deficiency is one possible cause of his gum disease, and the inflammation seen on his lower jaw that was active at the time of his death [Os]. Alternatively, the inflammation may have been caused by an infection. He had another infection or minor injuries between one and a few years before death that had affected his upper leg and upper arm bones; this had fully healed by the time he died [Os]. He also had slight periodontal disease [Os], possibly associated with the presence of *Porphyromonas gingivalis* in his calculus [Pr]. Like many of his comrades, he had probably inhaled smoke, as suggested by the presence of micro-charcoal and soot in his calculus [Mi]. He died aged 17–19 [Os].

#### Skeleton 24

Sk 24 was born in 1631–1633 [Os]. He spent his childhood, at least after the age of 3, outside the British Isles [SrO], His oxygen isotopes show very similar changes to Sk 5 but his strontium isotopes in early and middle childhood differ slightly, perhaps indicating a migration pathway via a different region but starting and ending up in the same place [SrO]. He was breastfed during infancy [De]. Dental enamel hypoplasia [Os] and perturbations in the isotopic profiles from his dentine [De] indicate that he experienced periods of chronic malnutrition or disease for much of his childhood, and his diet was low in animal protein from about ages 4–12. He had sinusitis [Os] which may have been caused by exposure to smoke as indicated by micro-charcoal and soot in his dental calculus [Mi]. His calculus also contained starch granules from his food [Mi]. Rising δ^15^N combined with falling δ^13^C in the last 4 years of his life suggest a period of nutritional or health stress [De] [52]. He died aged 17–18 [Os].

#### Skeleton 25

Skeleton 25 was born in 1633–1635 [Os]. During his childhood he moved among geologically slightly different areas, in the Midland Valley, Berwickshire, on the shores of the Moray Firth, further north in Caithness or the Orkney archipelago [SrO]. He was breastfed during infancy [De] and experienced multiple dietary or disease stresses in early childhood, causing enamel hypoplasia of his front teeth [Os] and indications of stress in the isotopes of his dentine [De]. His diet included oats [Pr], and this suggests a Lowland rather than a Highland origin. Although he was exposed to smoke [Mi], he shows no bony evidence for sinusitis [Os], but organisms causing respiratory disease (*Streptococcus pneumoniae* and *Bordetella bronchialis*) were present in his calculus. Sk 25 had extremely poor dental hygiene, with advanced tooth decay for one so young, and calculus deposits on 23 of his 32 teeth [Os]. The decay had almost completely destroyed the crown of one of his lower left molars and a large cavity in the adjacent tooth exposed the pulp chamber [Os]. He also had inflammation of his jaw, which might have been caused by infection or scurvy [Os, Sc]. He possibly used his teeth as a ‘third hand’ as wear on his canines (S1K Methods) and first premolars indicates he may have pulled coarse material through his teeth [We]. Increasing δ^15^N with unchanging δ^13^C suggests a period of nutritional or health stress in the last year of his life [De] [52]. He died aged 15–17 [Os].

#### Skeleton 26/27C

Skeleton 26/27C was born 1634–1636 [Os]. Their sex is unknown [Os], and the remains were less than 20% complete. On both tibiae there were destructive lesions which may have been caused by a benign neoplastic disease [Os]. Sk 26/27C died aged 14–16 [Os].

#### Skeleton 27A

An older soldier, Sk 27A was born in 1605–1614 [Os]. He spent his middle childhood outside of Scotland, perhaps in northern or eastern Europe, including places such as the Bohemian massif, Poland, Sweden or Norway east of a line from Trondheim to Oslo [SrO]. His early childhood, however, had been spent in a different location: northeast Scotland cannot be excluded, but parts of Sweden and Norway are also possible [SrO]. He endured multiple episodes of stress through his childhood, as indicated by enamel hypoplasia on his premolars and first molar [Os], and by the isotopes in his tooth dentine [De]. Not unexpectedly for his age his teeth were fairly worn, he had lost one tooth during life, and two-thirds of those remaining had calculus [Os]. His lower right canine was impacted within the jaw, and he had retained his small ‘milk’ canine into adulthood [Os]. A fracture to his lower first molar could have made eating difficult [Os]. Despite the poor state of his teeth, there was no sign of caries in those that remained [Os], perhaps suggesting he ate a diet with less sugars or starches than some of the other men. Like many of his comrades, he also had sinusitis [Os] (S1L Methods) which may have derived from smoke inhalation. Is it conceivable that Sk 27A was a foreign mercenary, possibly German or Swedish, or the returning son of a Scottish émigré from Sweden or Poland? He died aged 36–45 [Os].

#### Skeleton 27B

This man was born in 1632–1634 and died aged 16–18 [Os]. These remains were less than 40% complete.

#### Skeleton 28

Skeleton 28 was born in 1630–1634 [Os] most likely in the western Midland Valley, or further north in Caithness or the Orkney archipelago [SrO]. He may not have moved before the age of eight, but spent his later childhood in another location, possibly elsewhere in the Midland Valley or Aberdeenshire [SrO]. He was breastfed during infancy [De]. He experienced stress episodes due to disease or dietary deficiency in early childhood and again in his adolescence, as indicated by enamel hypoplasia of his tooth enamel and cribra orbitalia in his left orbit [Os]. The isotopes in his tooth dentine also indicate periods of stress [De]. His diet included wheat, presumably as bread, as indicated by the proteins in the moderately thick calculus deposits on his teeth [Os, Pr]. At some point he had a cyst or minor injury on his head, but it was very well healed [Os]. Like many of the Scottish soldiers he had sinusitis [Os]. He had a small cavity in a molar and slight periodontal disease, but his teeth were heavily worn for his age, leaving him with one potentially sensitive, painful tooth due to exposure of the pulp cavity [Os]. These odd wear patterns, with less wear on the lower teeth, and a notch in an upper front tooth (S1M Methods) were probably caused by habitually holding abrasive material between them [We]. This wear might be related to his occupation, for example, it might be predicted in a fisherman who mended nets and drew ropes or sinews through his teeth. He died aged 16–20 [Os].

### A prosopography of the soldiers

Above we collated the osteobiographies of the men, and now we analyse them prosopographically as a group to address our research questions.

#### Recruitment areas and childhood residence

Although the isotope ratios of the men are diverse, indicating no single place of childhood residence, the majority of the values are compatible with residence in the Midland Valley and northeast Scotland. This is concordant with the historical record, which indicates that many of the foot and cavalry regiments at Dunbar were recruited in these areas (Fig 3). We cannot tell precisely where these men came from, but it is possible that some subset(s) of them were known to one another before joining the army, taking these social and familial networks with them. These networks are evidenced for the survivors deported to New England, where brothers and childhood neighbours are attested [1: 217]. Such networks may also have contributed to selection and volunteering processes during their recruitment, and led to greater homogeneity of the group in terms of patronage and social identity.

Three men (Sk 5, 24 and 27A) are identified as having spent at least part of their childhood outside the British Isles. Sk 5 and 24 show remarkably similar strontium and oxygen isotope ratios (Fig 6) and are also close in age. Although they may have moved between similar regions c.1631-1644, their dietary histories differ greatly (Fig 8). Most references to foreign mercenaries in the Scottish army are to Dutchman and High Germans in cavalry units [20]. This implies they came from areas speaking Dutch, Low German or High German, but these areas are not easily distinguished isotopically from lowland Scotland. These men are more likely from somewhere further east or north, and perhaps are returning sons of émigrés from the 17^th^ century diaspora of many thousands of Scots across northern Europe [66]; indeed the Scottish commander David Leslie had spent time in the military service of the King of Sweden [20: 156]. The personal lives of these three individuals are exceptional when compared to other members of the group.

Diet may also depend on location, but there are few contemporaneous changes in the dietary isotope profiles of these men (Fig 8) to suggest common places of residence. One exception is that Sk 2 and 5 show a very similar increase in marine food consumption for several years about 1638 to 1642, shifts which largely post-date the formation of enamel and the isotope data from it. One might speculate that Sk 5 had moved to Scotland by this time and experienced a similar food shortage to Sk 2, perhaps in a coastal location where marine foods were consumed more frequently for a period.

#### Diet

All the men were breastfed, which is unsurprising for the 17^th^ century when there was little alternative. Their diet through childhood and early adulthood contained a mix of animal and plant protein with little input from fish except during some short periods for some of the men. A few exhibit lower proportions of animal protein in childhood. The foods evidenced in their dental calculus differ between the men but the broad spectrum of dairy, wheat or similar grasses, legumes, brassicas, and, most frequently, oats, corresponds to the components of a 17^th^ century lowland Scottish diet [28]. The identified dietary components may vary between men due to differences in diet, but we consider it more likely that this is random variation in the trapping and survival of food debris in calculus.

#### Health

In early childhood these men faced a series of diseases and health issues. Periods of starvation or illness (evidenced in isotopic changes and dental enamel hypoplasia) occurred from birth onwards for all those born after 1605, irrespective of when in that period they were born. Although discussion of famine and deprivation tends to be framed on a national or regional scale [29, 32], the incremental dentine data (Fig 8) suggest a much more variable experience, varying significantly in date locally, or even individually. One third of the men had vitamin D deficiency in infancy or childhood, presumably due to lack of sufficient exposure to sunlight–a problem as much due to the high latitude of Scotland as to any cultural practices [67, 68]. In later childhood some had periods of shifting diet. Most men had probably had some level of scurvy (vitamin C deficiency) several years preceding death, but generally this had reduced in intensity or ceased in the more recent years of their lives. A widespread shortage of fresh fruit and vegetables is implied, perhaps in the late 1630s or early 1640s, followed by better, even adequate, availability in the years preceding 1650. However, the slow rate of bone turnover prevents any possibility of detecting vitamin C deficiencies specific to the last few months of their lives, except where woven bone could be sampled, and only one of those four individuals showed signs of scurvy. It is notable that the three young individuals who died while their teeth were still forming (Sk 1, 24, 25) all showed rising δ^15^N combined with flat or falling δ^13^C at the end of their lives (Fig 8). This pattern is also seen towards the end of the lives of those who died shortly after the completion of tooth formation (Sk 12, 22), suggesting they all experienced a period of nutritional or health-related stress [52]. This stressful period starts at various points between 1647 and 1649, a period coinciding with the end of the series of epidemics of the 1640s [33–35], the “great scarcity of victual” which prompted legislation in January 1649 [69], and the period of recruitment of many units in the Army of the Covenant I 1648–1650 [20]. Any or all of these events could have contributed to these isotopic changes.

Other long-term health issues included inflammations of unknown cause, particularly in the legs, arms, and jaws, which were sufficiently extensive and long-lasting to affect the bone as well as the overlying soft tissue. Some lesions were active at the time of death and others had healed or were healing. Notably, the active and healing lesions were restricted to adolescents, and it could be argued that they would have been experiencing the physiological demands of growth and therefore would have been particularly vulnerable to restricted food intake. This may have left them more vulnerable to developing infections in general [70, 71], a potential cause of the inflammatory lesions observed. Diarrhoeal disease, such as the outbreaks of dysentery many of the soldiers experienced before and after the battle, would have increased the risk of further infections through the depletion of crucial nutrients and compromising the immune system [70]. Alternatively, their skeletal response to infections may have been more sensitive than that of the adults, or these lesions may have been linked to the development of scurvy. However, there was no immediately apparent correlation with the scurvy biomarkers, though this may be because the skeletal responses to scurvy of new bone formation and hematoma organization represent a repair mechanism which can only be initiated following the restoration of sufficient dietary vitamin C [72].

Reaction to pathogens over the long-term is also evidenced in calculus where the body’s defences against bacteria, reaction to inflammation, immune response and host defence mechanisms are seen. Organisms causing respiratory diseases were also present, but we cannot tell if this represents disease active at death, remnants of a past infection, or merely exposure to a pathogen without developing disease.

Peptides from causative agents for periodontal disease and periodontitis were present in some men (Fig 12) as were signs of periodontal disease [1: 70]. Dental diseases were common, including caries and abscesses, though the youth of the group means they were probably less common than in the population at large [1: 71].

Interpersonal violence seems to have been rare in the lives of these men. Maximally there are one knife wound, one broken finger, one broken rib, and two fractured molars. The latter injury typically occurs following a fall onto, or blow to, the underside of the jaw [73], but in one man a cavity in the tooth had weakened the crown making it more likely to fracture. There is no evidence for blunt force trauma to the skull, parry fractures or other injuries characteristic of interpersonal violence. It seems that, apart from military activity, Scottish society of the second quarter of the 17^th^ century was relatively peaceful.

Chronic sinusitis and occurrence of burnt plant matter in calculus was ubiquitous, and both are likely to be consequences of exposure to pollution in the form of soot. This is not unexpected given the smoky living conditions (see above *Living conditions*) and popularity of tobacco smoking. If some surviving prisoners were sent to work in salt production because they already had experience, then it is also possible that some of those who died also had that occupation where there would have been a very high exposure to smoke [74]. However, given the ubiquity of exposure in these men and our inability to quantify levels of exposure, this must remain speculative.

The other pollutant for which we have evidence, lead, is present at low levels which might represent entirely low-level natural exposure. However, the changing isotope ratios with concentration suggest some exposure to anthropogenic lead from Scottish ores, probably through the use of lead utensils [1: 94–96].

Unusual wear on teeth for five individuals (Sk 5, 12, 19, 15 and 28), may be associated with using the teeth as a third hand in their occupation. This might have been linked to weaving, of which we know some prisoners had experience, though the wear cannot be specifically assigned to a particular occupation.

## Conclusions

In this study we have been able to use a panoply of archaeological science methods to study in detail, with relatively high chronological precision, the lives of these men, and make comparisons to the historical record. Table 4 gives some summary comparisons of information from the historical record with our findings. This illustrates that our findings are concordant with the expectations from the historical record in many respects, but other aspects we have been unable to investigate and in yet others, we have information not available from historical sources. We have been able to comment on the availability of non-staple foodstuffs such as fruit and vegetables. Matters which would not have resulted in acute illness or withdrawal from the labour force were unlikely to be recorded by contemporary writers, but some of them are visible in our study at high frequencies, such as dietary deficiencies in Vitamins C and D, while others such as interpersonal violence seem to be lower than might have been anticipated. This illustrates that even in the relatively richly documented Early Modern period, there is value in the application of archaeological science to confirm, challenge and complement the historical record.

**Table 4:** Summary comparison of historical evidence reviewed in the historical context section and the results of the archaeological science studies of this paper.

Topic	Historical evidence	This paper
Recruitment locations	Southern and northeast Scotland, some European mercenaries.	Compatible with the Midland Valley and northeast Scotland, with some from overseas.
Diet composition	Staples of wheat, oats, peas, beans, kale, and dairy in the Lowlands. Low levels of marine foods.	All these staples evidenced but not quantified. Marine foods are low on average but higher for some individuals for short periods.
Dietary vitamin C	No evidence.	Evidence of shortages, especially in the late 1630s and into the 1640s
Vitamin D deficiency	No direct evidence, but predicted to be present from latitude.	One third of men show skeletal evidence for deficiency
Food shortages and diseases	National and regional shortages in specific years. Epidemic disease and deaths in specific years.	Few simultaneous widespread dietary or health stresses but evidence for local or individual dietary stresses at varied dates. One shared stress may be 1649 shortage or effects of army recruitment.
Everyday inter-personal violence	Limited evidence of extent.	Small sample size but remarkable low levels of skeletal injuries.

The men whose remains we have studied here were recruited to the Scottish army, determined by Cromwell after the battle of Dunbar to be fit enough to fight again, and survived the 170 km march from there to Durham. They were thus by no means the weakest and most susceptible to disease in the Scottish population. The mortality amongst the prisoners at Durham was very high at over 53%. Both these factors imply that the osteological paradox—that frequently in human osteoarchaeology we are studying those with highest frailty and selective mortality, unrepresentative of the living population [75, 76]—does not have a strong effect in this case. The life conditions we see evidence for in this sample of the population probably represent those of the wider Scottish population of the period, during which many people experienced periods of malnutrition and vitamin deficiency, even after the shortage years of the 1620s had passed. Price controls resulting from shortages focussed on grain, leaving fruit and vegetables as an element of diet where the documentary sources give little evidence. Specifically, we infer a lack of fresh fruit and vegetables, perhaps in the early 1640s, which ameliorated in the latter part of the decade.

If mortality during the imprisonment at Durham was not selective, then these men must have been comparable to those who survived and were deported. Many of those prisoners who settled in Massachusetts and Maine lived long lives, even into their eighth and ninth decades [1: 236]. Thus, although the deprivations they suffered were at times severe, or appear to be, the long-term effects were not severe.

The army of which these men were members was raised by a centralised system that allocated quotas for each shire and parish, with local church leaders responsible for listing eligible men and seeing that the quotas were filled [20: 3–4]. The group investigated here reflects that system with a wide range of places of origin within Scotland, but also with mercenaries or returning émigrés. They are otherwise relatively similar in terms of diet, and although they show variation in childhood disease and nutritional stress, only the older man, Sk 6, stands out as notably different. As a group, they seem to have been fairly homogenous, perhaps implying that they were drawn from the same stratum of society.

It would be interesting to learn about the impacts of joining the army on these men and more about the conditions during their imprisonment, but sadly neither of these are identifiable in the evidence we have. Diseases such as dysentery affecting them during this period would not have lasted long enough to be chronic and thus affect bones or teeth. Some of our observations, such as the presence of woven bone, may reflect their experiences in the weeks prior to death, but cannot be interpreted in any specific fashion. In general, however, for almost all aspects of diet and disease we do not have the temporal resolution to separate events within the last year of life.

What we have been able to do is to use the precise dating and multiple scientific analyses to provide unprecedented historical and biographical detail for archaeologically recovered individuals. Despite being found in England, they may be the most closely studied Scottish skeletons from the 17^th^ century. These men were not exceptional or special people, just ordinary Scottish soldiers. We cannot try to understand their personality, but when the results presented here are combined with the facial reconstruction of one individual [1: 76–78] we hope that we have given them a voice and put flesh back on their bones by telling their life-stories.

## Supporting information

S1 Methods Detailed methods and previous results. (DOCX)

S1 Table Strontium and oxygen isotope results. (DOCX)

S2 Table Incremental dentine results. (XLSX)

S3 Table Tandem mass-spectrometry run order and information on proteomic files uploaded to the MassIVE repository. (XLSX)

S4 Table Putative dietary peptides identified within the dental calculus samples. (DOCX)

S1 Fig Incremental dentine δ^15^N and δ^13^C collagen profiles for permanent canine and M3 for each individual by approximate age at of formation. (DOCX)
