# Supplementary material for: Scottish soldiers from the Battle of Dunbar 1650: A prosopographical approach to a skeletal assemblage
Source: PLoS One. 2020 Dec 21;15(12):e0243369. doi: 10.1371/journal.pone.0243369 (PMC7751964; doi:10.1371/journal.pone.0243369)
Supplement: S1 Methods — (DOCX) [file pone.0243369.s001.docx]

The Scottish soldiers from the Battle of Dunbar 1650: a prosopographical approach to a skeletal assemblage

# **S1 Detailed** **methods and previous results**

## Detailed methods

### Summary of osteological methods

The skeletal remains were analysed in full. Their state of preservation was recorded through examining their completeness (expressed as a percentage) and condition. Surface preservation was assessed using a seven-category grading system, ranging from 0 (excellent) to 5+ (extremely poor) [1]. Excellent preservation implied no erosion of the bone surfaces with clear surface morphology, whereas extremely poor preservation indicated heavy and penetrating erosion of the bone surface resulting in complete loss of surface morphology and modification of the bone shape. The extent of fragmentation evident was assessed subjectively, and categories of fragmentation ranged from minimal (indicating little or no fragmentation of the bones) to extreme (indicating extensive breaking of most bones into multiple small pieces).

Where preservation allowed age-at-death, sex, and stature were estimated. Age was determined using standard methods [2]. For adults, age was estimated from degenerative changes seen in the pelvis [3, 4] and ribs [5-7], supplemented with analysis of dental wear [8]. For non-adults, age was estimated based on the stage of dental development [9, 10], dental eruption [11], skeletal development and measurements of long bones [12].

Assessment of sex was carried out on adult individuals though examination of the shape of the pelvis and skull (cranium and mandible), supplemented with measurements of certain bones[13]. Measurements were taken where possible and used to calculate stature [14]. Finally the skeletons were examined for any evidence of disease or trauma [15, 16].

### Dental microwear

The term dental wear is used to describe the loss of hard tooth tissue (enamel) caused by friction on the tooth’s occlusal surface, by opposing teeth or another material during masticatory or non-masticatory actions [17]. Notching and chipping of the incisors and canines can be associated with specific activities, only some of which can be linked to eating.

The relevant teeth of the selected individuals were examined using a dissecting light microscope and a scanning electron microscope at Durham University to identify scratches and chips and their orientation and topography. Observations were interpreted using published schemes for directional wear [18, 19] and identification of chipping [20, 21].

### Microscopic analysis of dental calculus

The preparation and examination of the samples were conducted in a dedicated clean laboratory at the University of York under controlled conditions to avoid contamination. Following a now established protocol [22], particularly useful with small samples of calculus, the external surface of the calculus was cleaned manually using an acupuncture needle and a weak hydrochloric acid solution under a dissecting microscope at a magnification up to 50× to remove contaminants that might have adhered to it. The decontaminated dental calculus matrix was dissolved in a sterile tube using 0.6 M HCl, freeing the small particles that had been entrapped during its formation. The suspension was mounted onto microscope slides and observed under high magnification (up to 630×). All particles were counted and wherever possible identified using a large reference collection and published resources [e.g., 23, 24, 25].

### Isotope analysis of enamel

**Strontium isotopes**

Enamel samples were prepared following previously published procedures [26, 27]. They were dissolved in 16M HNO_3_, dried down and redissolved in 3M HNO_3_. The samples were loaded on columns containing Eichrom Sr resin, strontium was eluted with water and acidified to 3% HNO_3_ for analysis. All reagents were Teflon distilled and ultrapure Milli-Q system water was used.

Strontium isotope ratios were measured using a ThermoFisher Scientific Neptune Multi-collector ICP Mass Spectrometer (MC-ICP-MS) in the Arthur Holmes Isotope Geology Laboratory, Durham Geochemistry Centre at Durham University. Reproducibility of the standard NBS987 during analysis of M2 samples was 0.710258 ± 0.000011 (2σ, n=12), and for M1 and M3 samples was 0.710263 ± 0.000013 (2σ, n=10). All values have been normalised to the accepted value of 0.710240 for NBS987.

The results were interpreted as potential geographical origins by comparison with the map and data of Evans and coworkers for Great Britain [28] and Snoeck and co-workers for Ireland [29]. The mapped areas of the ‘isotope packages’ that lay within of ±0.0002 of the strontium isotope ratio, and the locations of any individual data points within the same margin, were considered as possible places of origin in the osteobiographies

**Oxygen isotope analysis**

Enamel samples were prepared following previously published procedures [27] based on the rapid precipitation of silver phosphate [30]. Samples were dissolved using 2M HF and 2M HNO_3_ to separate calcium as fluorite. The solution was neutralised and ammonia and silver nitrate added to precipitate fine-grained silver phosphate. After washing and freeze-drying, 0.15-0.20 mg of silver phosphate was weighed into silver capsules and submitted to the Laboratoire de Géologie de Lyon (CNRS-UMR 5276, Université Claude Bernard Lyon, France) for isotopic measurement in duplicate following published procedures [31].

Samples were prepared in two batches, first the M2 samples then several months later the M1 and M3 samples. Replicate measurements on NBS120C prepared with the M2 samples yielded a δ^18^O value of 22.19±0.63 ‰ VSMOW (1σ, n=3). Measurements on NBS120C prepared in the Lyon laboratory by the method of Lécuyer et al. [32] gave a value of 21.77±0.37 ‰ VSMOW (1σ, n=11) for M2 analyses and 21.58±0.19 ‰ VSMOW (1σ, n=10) for M1 and M3. All values for NBS120C are within error of the accepted value of 21.7 ‰ (summarized in [33]). The technical error of measurement from all the duplicate samples in the first batch was 0.35 ‰, and in the second batch 0.20 ‰. Drinking water values (δ^18^O_DW_) were derived from phosphate (δ^18^O_P_) values using the calibration of equation 6 of Daux et al [34] for humans.

**Interpretation of origins**

Strontium isotopes were used to identify potential regions of origin using the dataset and isotope packages of Evans et al.[28]. For each tooth analysed, a range of ±0.0002 was taken, and a map produced of environmental samples within that range and isotope packages that overlap that range. Calibrated δ^18^O_DW_ values were compared to maps of expected values for Scotland and Europe [35-37], and the regions identified using strontium isotopes were considered more or less probable in the light of this.

A reviewer suggested we could have used the British Geological Survey’s online Biosphere Isotope Domains mapping tool at <http://mapapps.bgs.ac.uk/biosphereisotopedomains/index.html>. This is largely based on the same dataset for strontium, but has the disadvantages that (a) we cannot map simultaneously all isotope domains within ±0.0002 of a measurement and (b) omits individual environmental data points that might be close to a measurement. For oxygen isotopes the Biosphere Isotope Domains mapping cannot handle measurement and calibration uncertainties. For these reasons we believe our approach is more conservative, providing wider geographic results, than using this tool.

### Incremental dentine δ^13^C and δ^15^N analysis

Each tooth was cleaned by air abrasion, a single root was removed from the molar teeth and the canine teeth were bisected, with the bulk of the enamel removed from the sampled portion using a hand-held saw. There were no obvious macroscopically visible areas of caries or secondary dentine present on any of the teeth sampled.

Each sample was demineralised in 0.5 M HCl at ~4 °C following the modified Longin method [38] and then sectioned according to the second method in [39] using a scalpel. For each tooth, 1 mm horizontal samples were taken down its length from crown to root apex. All demineralized dentine sections were denatured by heating to 70 °C in a pH 3 solution for 24 hours, frozen and then freeze-dried.

The samples were measured in duplicate at the Stable Isotope Laboratory, University of Bradford. They were combusted in a Thermo Flash EA 1112 and the separated N_2_ and CO_2_ was introduced to a Delta plus XL via a Conflo III interface. Laboratory (bovine liver substrate and fish gelatine) and international standards (N1, CH3 and IAEA600) were interspersed with the samples, and the analytical error was determined to be ±0.2‰ (1sd) or less.

The age of formation of each increment was initially estimated from known growth rates as previously published [40], based on the London Atlas of Human Tooth Development and Eruption with a stated uncertainty of ±6 months [41] but recognising the wide variation in the formation times of third molars, the assignment of an age range to individual increments in these teeth adjusted to account for the overall age-at-death estimate for each individual.

### Metaproteomic analysis of dental calculus

**Sample Preparation**

Sample preparation and protein extractions were conducted at BioArCh, University of York. Samples were ground with sterile micropestles. Between 3 and 25 mg of dental calculus was crushed to a powder, and pre-digested for 5 minutes with 1 ml of 0.5 M EDTA to remove possible surface contamination. The supernatant was replaced with a further 1.0 ml of 0.5 M EDTA, and rotated at room temperature for seven days to fully demineralize. After centrifuging at 13,000 RPM for 2 minutes, 950 μl of supernatant was transferred into a new microcentrifuge tube and retained for future analysis. Proteins were isolated from the pellet and the remaining 50 μl of supernatant following a gel-aided sample preparation (GASP) method [42] modified as previously described [43].

Tandem mass spectrometry analysis was performed on a Q-Exactive (Thermo Fisher) at the Target Discovery Institute, University of Oxford, as previously described [44]. Raw spectral data were converted to Mascot generic format (mgf) using Proteowizard MSConvert (version 3.0.4743) using the 100 most intense peaks in each MS/MS spectrum. MS/MS ion database searching was performed on Mascot (Matrix Science^TM^, version 2.4.01), against the UniProt database. Searches were performed against a decoy database to generate false discovery rates. Propionamide (C) was set as a fixed modification and acetylation (protein N-terminus), deamidation (NQ), methionine oxidation, propionamide (K) and propionamide (N-terminus) were set as variable modifications. Peptide tolerance was 10 ppm, and MS/MS ion tolerance was 0.5 Da. Fully tryptic peptides were searched with up to 1 missed cleavage. Protein results were filtered to a significance threshold of <0.05 and an ion score of >25, and contain a minimum of two peptides. The raw data and Mascot search results are available in the public database on the MassIVE repository (ID: MSV000084258; http://massive.ucsd.edu) (file description and run order presented in S4 Table).

Protein results were filtered to a false discovery rate of less than 5%, a significance threshold p<0.05 and only proteins containing a minimum of two peptides were considered. We assigned MASCOT protein identifications into the following classifications: contaminants, human, non-human animals and plants [43]. We took a conservative approach and assigned any protein identified in our blank controls or injection blanks to the ‘contaminant’ category. Initially, all non-human animal and plant proteins were considered as potential dietary proteins, and were further interrogated using BLAST (NCBI). Any non-human animal or plant peptides that also matched identically to human or microbial proteins were not considered as possible dietary proteins. Likewise, any non-human animal or plant peptides deriving from proteins identified within the ‘contaminant’ dataset were eliminated as potential dietary proteins. Identified peptides were further interrogated using BLASTp to examine each peptide’s lowest common ancestor.

To confidently identify bacterial taxa within the proteomic datasets, the identified peptides were binned into two groups by length (Group 1: <15 residues; Group 2: >15 residues) and submitted to protein BLAST for sequence matching using the NCBI database with the following criteria: for Group 1 peptides an expected value of 20000 was used and PAM30 was selected as the Score Matrix; for Group 2 peptides an expected value of 1000 was used and BLOSUM62 was selected as the Score Matrix. The resulting BLASTp files were then parsed using MEGAN metagenomics software [45]; only proteins with 100% identity and at least two unique peptides were accepted as confident identifications.

### Scurvy biomarker analysis

Collagen is a structural protein that is ubiquitous in mammalian tissues and type I collagen (COL1) accounts for 90% of human bone proteins [46]. Vitamin C (ascorbic acid) plays a key role in the formation of collagen by acting as a cofactor in the conversion of various proline (and lysine) residues to their hydroxylated forms [47]. During their evolution, humans (along with many other primates, bats and guinea pigs) lost the ability to synthesise vitamin C from glucose; consequently, the vitamin C needed to convert proline to hydroxyproline must come solely from the diet (principally fruits and vegetables) [48]. The relationship between dietary vitamin C and hydroxylation levels in collagen containing tissues has been demonstrated in guinea pig feeding experiments [47, 49, 50]. The reduction in hydroxyproline varies between studies and after prolonged cessation is associated with a reduction in collagen formation. With complete removal of vitamin C from the diet, after only two weeks the bones of scorbutic guinea pigs showed a one third reduction in hydroxyproline compared to controls [50].

Proline hydroxylation is a major post-translational modification, which is essential to the folding and stabilization of the triple helical collagen molecule [51, 52]. Although the mechanism by which this stabilization occurs is still debated [53-55] a positive relationship has been observed between hydroxyproline content and mechanical stability [56, 57]. Under-hydroxylation of proline residues can thus have severe consequences for the human body, including weakening of connective tissues, defective, reduced or arrested osteoid formation and fragile blood vessels prone to hemorrhage [58]. The outcome of prolonged vitamin C deficiency on collagen containing tissues can be observed in the clinical manifestations of scurvy [59] and lesions associated with scorbutic skeletal material [16, 60].

As the dominant protein in bone collagen represents a good target for proteomic analyses of archaeological skeletal remains. In addition, collagen has been shown to survive into the fossil record in a largely unaltered state [61]. In recent years advances in protein mass spectrometry have meant that collagen mass fingerprinting (ZooMS) is now becoming routinely used for species ID [62, 63]. LC-MSMS of archaeological remains is also developing into an attractive tool for the characterization and/or species identification of complex tissues [64]. This tandem mass spectrometry approach is more complex and generates significantly more data; in addition to identifying the peptides in a sample (Peptide Mass Fingerprint) a second mass-spectral analysis of the peptide peaks allows sequencing of the amino acids within the peptides [65]. This latter approach was taken for the scurvy biomarker research as it was necessary to identify the *location* of the hydroxylation sites within the collagen peptides.

**Methodology**

**Sample preparation**

Small subsamples of bone were removed manually with a scalpel, Dremel drill or pliers. Before and after photographs were taken to document the sampling process. Collagen was extracted at the Stable Isotope Research Laboratory, University of Bradford, following a protocol similar to previous work [66, 67]. Briefly, the subsamples were manually crushed into small bone shards using a pestle and mortar. The shards were demineralized in 0.5M HCl at 4°C for up to 14 days and then washed approximately 5-10 times with deionised water to neutrality. The acid insoluble fraction was freeze-dried, weighed and 0.5mg was re-suspended in 6M urea at a concentration of 1mg/ml. 20μl was taken and enzymatically digested with Trypsin. The resulting peptides were then acidified, desalted, filtered and concentrated with C18 zip tips prior to LC-MS/MS analysis using the Q Exactive Hybrid Quadrupole-Orbitrap Mass Spectrometer (Thermo Scientific), at the Target Discovery Institute, Oxford University.

**Data Analyses**

The data analyses involved a two-step process. In the first instance raw data files in the form of peak lists were searched using a proteomic search engine to obtain peptide IDs and to assess the quality of the data. In the second step the data from collagen peptides were sorted and filtered to extract those peptides, which had previously been identified as under-hydroxylated in scorbutic individuals, i.e. the potential ‘scurvy biomarker peptides’. The MS/MS spectra from these collagen peptides were then manually checked to ensure that there were sufficient fragment ions for confident peptide and hydroxylation identification.

MS/MS raw peak lists were searched against the Uniprot human protein database (UPR_HomoSapiens) to obtain peptide IDs and to assess the quality of the data. The Mascot search engine (Matrix Science version 2.5.1) was used with the following parameters: trypsin digestion; maximum 1 missed cleavage; no fixed modifications; variable modifications: proline hydroxylation (P), methionine oxidation (M), Gln->pyro-Glu (N-term Q), lysine hydroxylation (K) and deamidation (NQ); a 95% confidence interval threshold (p < 0.05); peptide mass tolerance of 7 ppm and fragment-ion mass tolerance of 0.03 Da.

Collagen peptides pertaining to the protein sequences COL1A1 and COL1A2, and previously been identified as under-hydroxylated in scorbutic individuals, were extracted from the final refined list from 93909 sequences. Spectra were accepted with an FDR 1% and ion score above 20. COL1 sequence coverage for both chains was generally 80-90%. To ensure that there were sufficient fragment ions for confident peptide and hydroxylation identification, the data were manually filtered against the protein identifier, peptide sequence, peptide mass and ion score. All MS/MS spectra pertaining to the 12 scurvy biomarker peptides were then manually checked to assess the quality of the spectra and to identify the presence or absence and location of a +15.9994 Da mass shift corresponding to a hydroxylation. Finally, those of the 12 biomarker peptides that contained the under-hydroxylated variant were recorded for each sample.

Considerations

A limitation to this approach remains our current lack of understanding about the true number and variability of hydroxyprolines in non-pathological collagen (Krane 2008). Our preliminary work on non-scorbutic modern human, guinea pig and bovine bone has suggested that at some proline sites there are variable hydroxylations, i.e. the same peptide can be present with and without a hydroxylated proline [68]. This observation has also been noted in human type 1 collagen from breast tissue [69]. Based on our previous analysis on non-scorbutic material, in this study we have used a maximum baseline of 4 under-hydroxylated peptides above which a collagen sample is described as ‘under-hydroxylated’ and thus potentially scorbutic. However, extensive, detailed mapping of hydroxylation sites within different skeletal tissues and across different species is still needed before we have a complete picture of proline hydroxylation in bone collagen.

## Summary of previously published results

### Excavation

Summary account based on the interim excavation report [70].

Between 5^th^ November and 13^th^ December 2013I Archaeological Services Durham University monitored ground‐disturbing works associated with building work in an internal courtyard at Palace Green Library, Durham, UK. The ground level in the courtyard is above that of Windy Gap, a path runs across the south end of the site and is separated from it by an old brick wall. A flight of steps ran down to a door in this boundary wall inside the site. At the south end of the site the ground level was to be reduced to create a new curved flight of steps linking the south door to the to the ground level of the courtyard.

Among other excavated features, two pits [F512 and F514] cut into yellow sand [F507] were exposed, both of which contained human remains. Pit F512 at the north‐east corner of the area of ground-level reduction, had a steeply sloping side and a flat base. Eighteen skeletons [SK1‐2, 5, 9, 12, 15, 18‐29] were identified within this pit. The remains were covered with a yellowish grey silty sand [511]. Above this was ad eposit of greyish yellow sandy silt with sandstone chippings [518: 0.5m deep]. Pit F514 had been truncated in the past, during construction of the old south steps. Burials only survived directly under the boundary wall between the courtyard and Windy Gap. Eleven skeletons [SK3, 4, 6‐8, 10, 11, 13, 14, 16, 17] were identified with in this pit. The skeletons were covered with a yellowish grey silty sand [513]. It is presumed that each of these pits represents a single episode of burial.


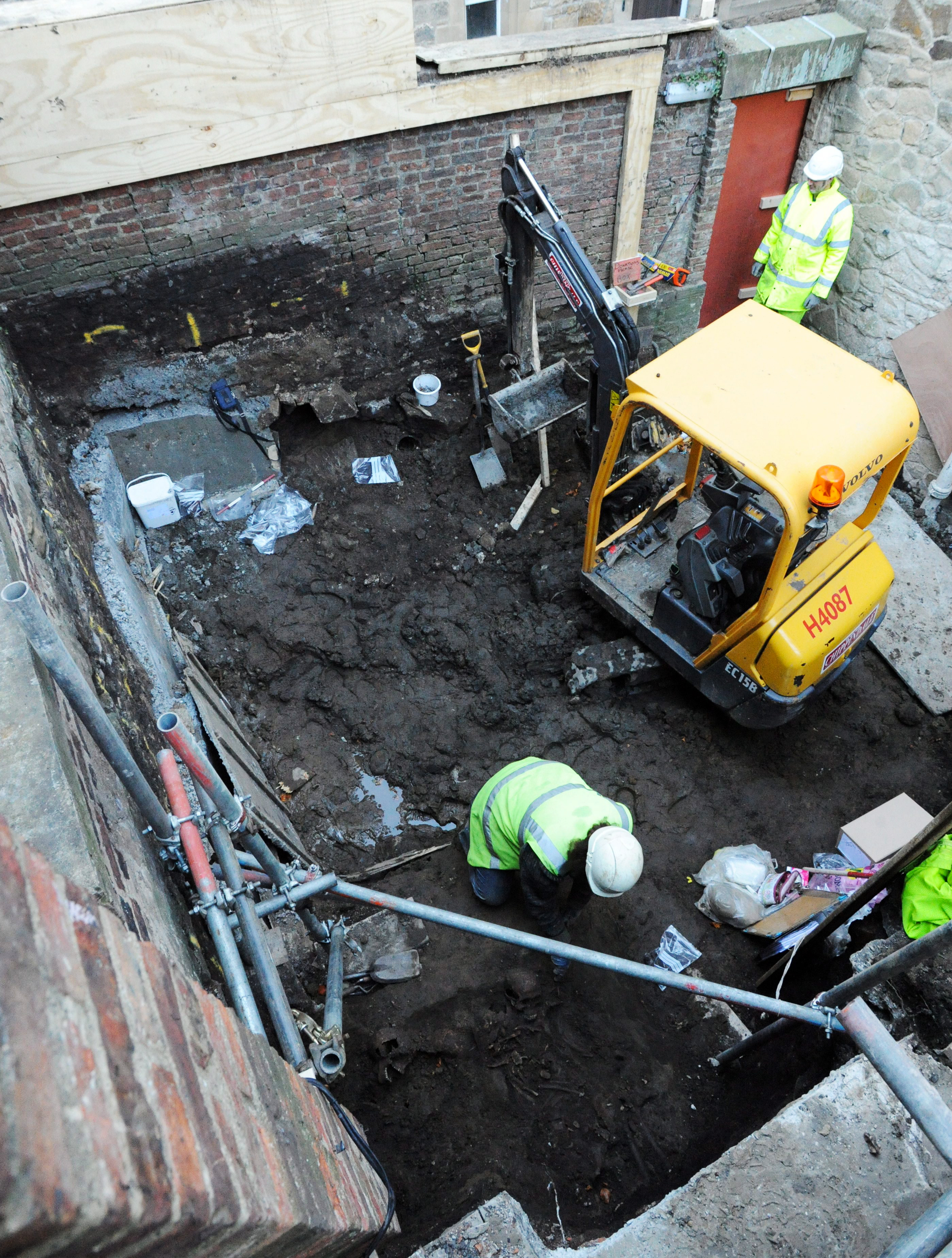


Figure A. Overview of excavation in the area of ground reduction from the northeast corner. Feature F512 is in the bottom centre, feature F514 to the middle left.


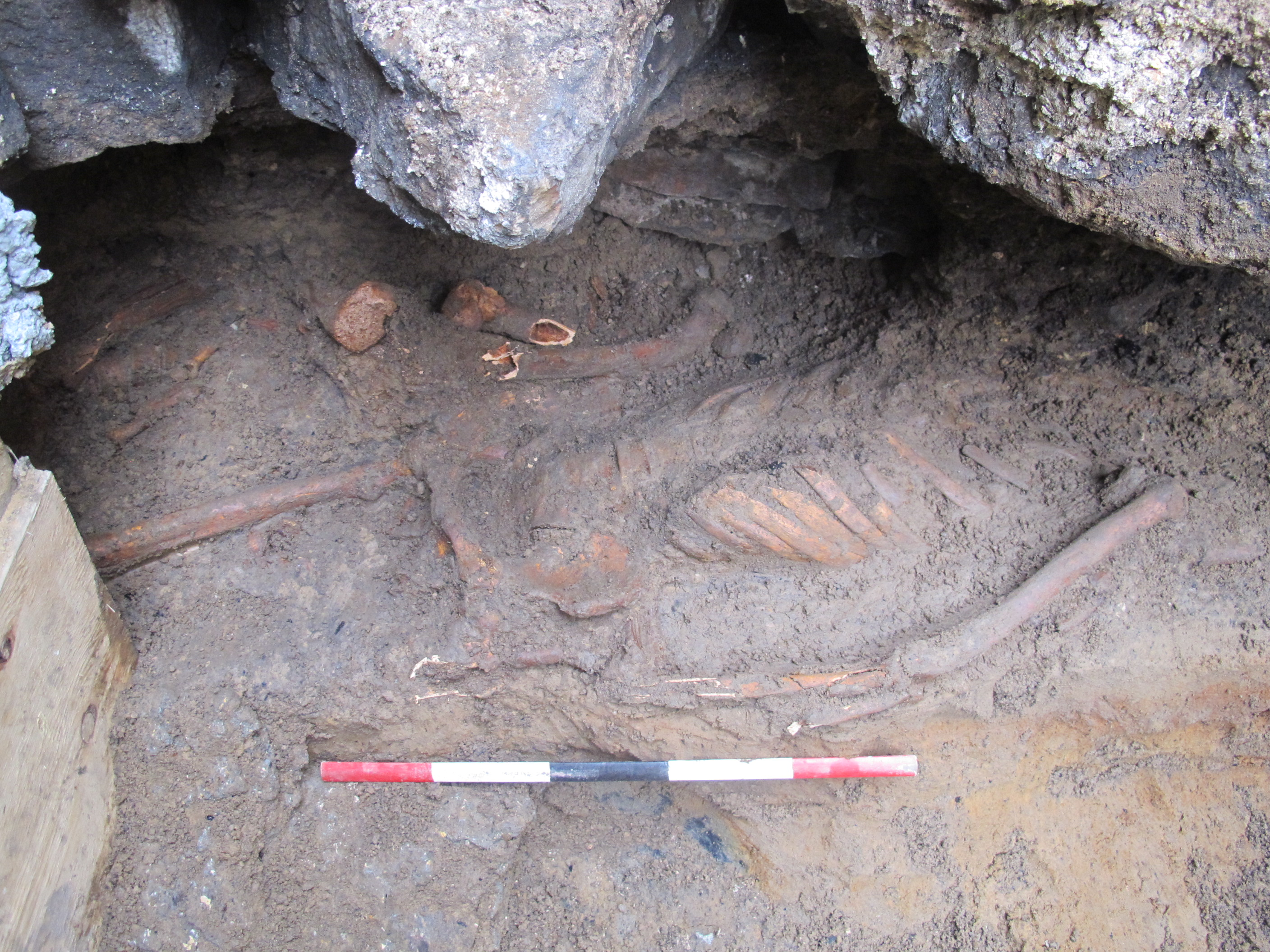


Figure B: Feature F514 under excavation showing Skeleton 6 under the foundations of the boundary wall with Windy Gap at the south end of the site. Scale 0.5 m. North to the bottom.

### Radiocarbon dating

Radiocarbon dating of the burials was previously reported [71]. Initial dates were obtained on long bones of Sk9 and Sk16A, and then a more detailed study was undertaken using slices of dentine from first and third molars of Sk12 and Sk21. Using the known difference in age of formation of the dentine of about 10 years, a series of alternative models were constructed ‘wigglematching’ the dates. The different models allowed for a terminus post-quem of 1612 or 1620 for tobacco consumption evidenced by the pipe-facets in the teeth, different estimates of the local marine reservoir correction, and exact or uncertain difference in age between the dated teeth. Considering all these possible influences on the radiocarbon dates and the other constraints on dating, the modelling suggested the burials took place between 1625 and 1660 (Figure C).


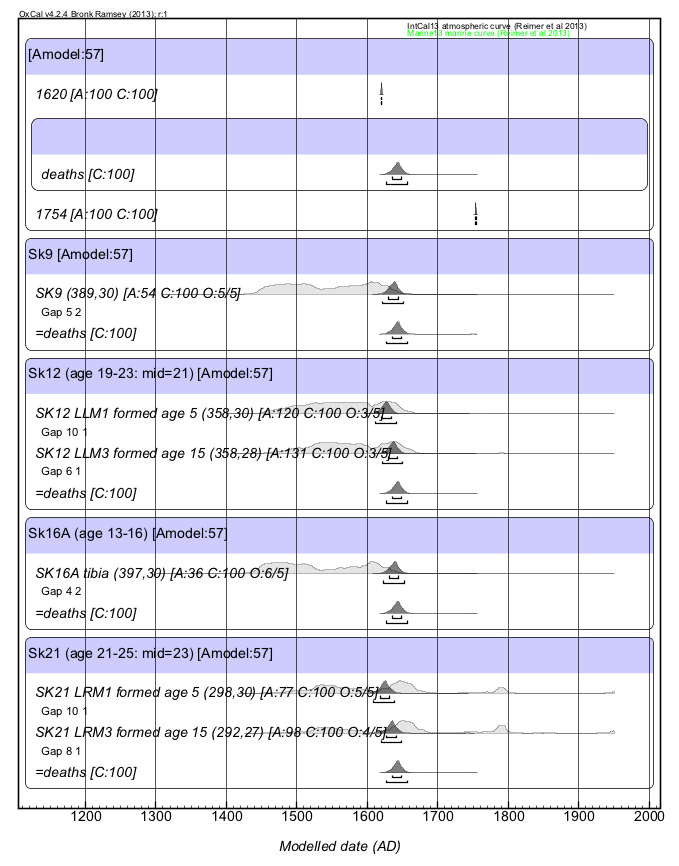
 Figure C The final preferred model, denoted IID in the report.

### Osteological results

We present here selected illustrative evidence from the osteological analysis to show some of the changes to bones and teeth.

(a)
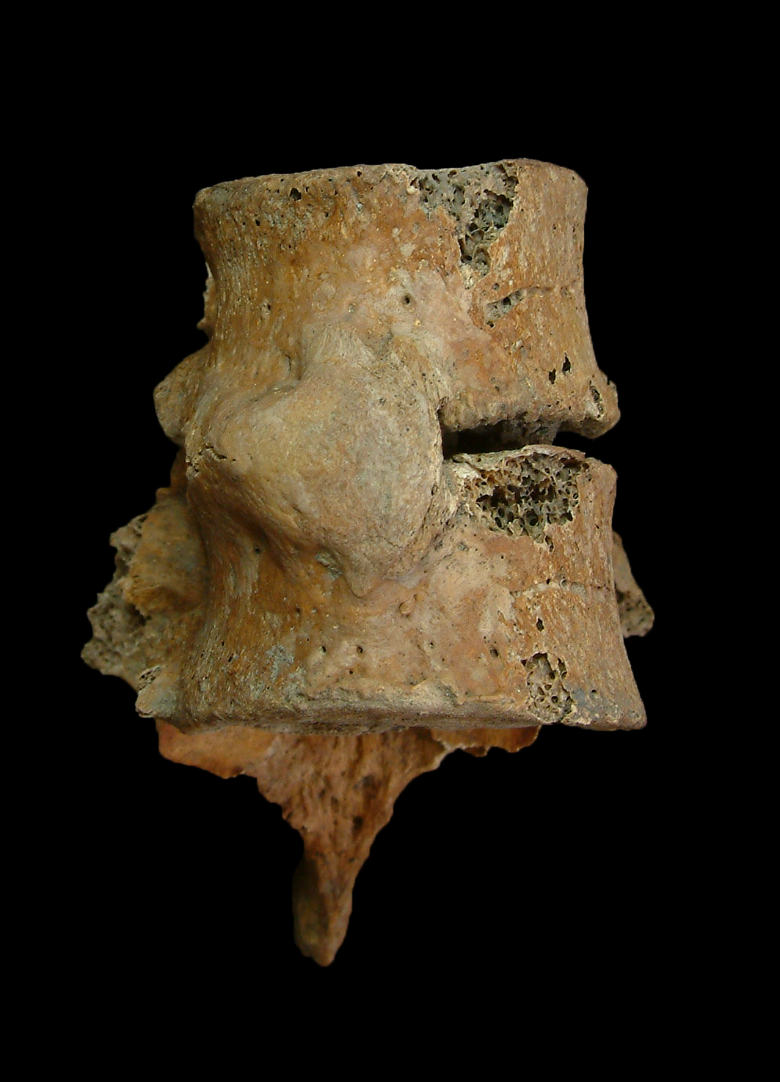


(b)
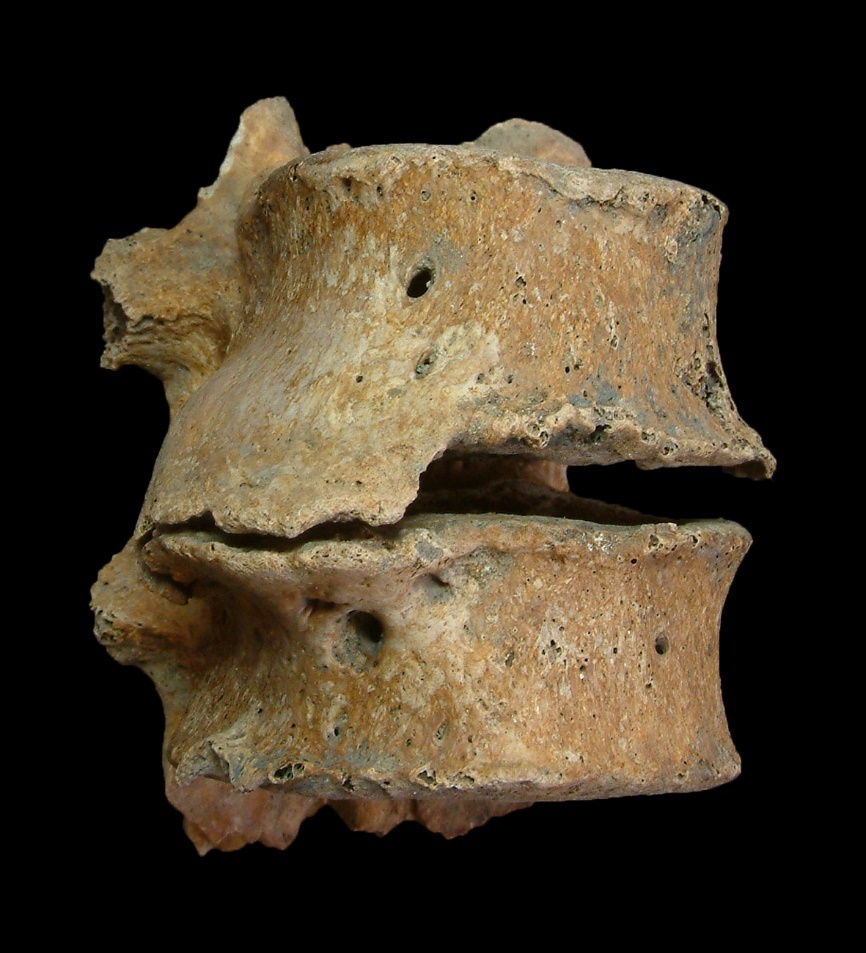


Figure D (a) Sk 6 fusion of thoracic vertebrae 7 and 8. (b) Sk 6 large osteophytes between thoracic vertebrae 10 and 11. These changes are suggestive of the early stages of the diffuse idiopathic skeletal hyperostosis (DISH) but are not diagnostic


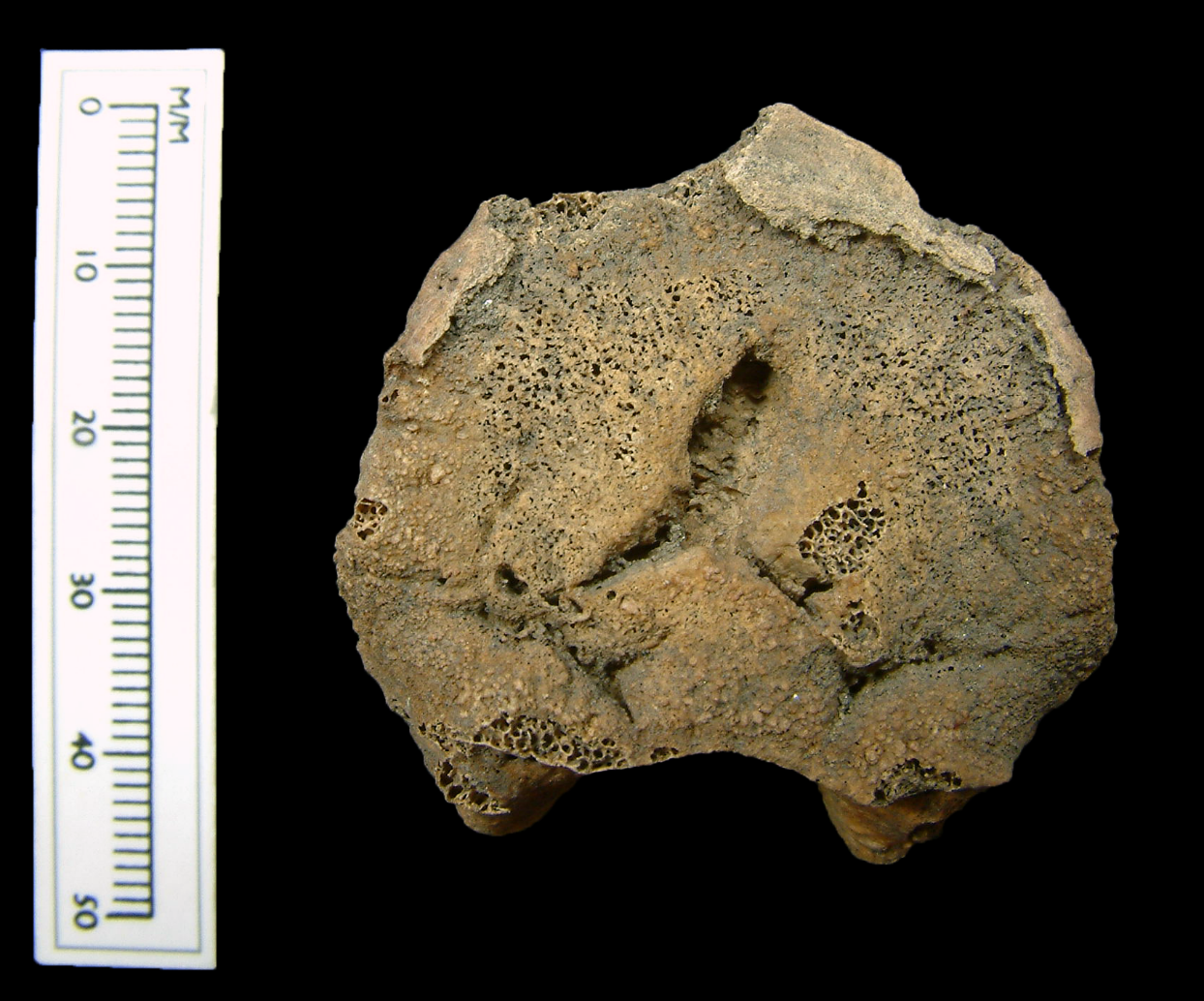


Figure E. Sk 12 thoracic vertebra 9 showing Schmorl’s nodes.


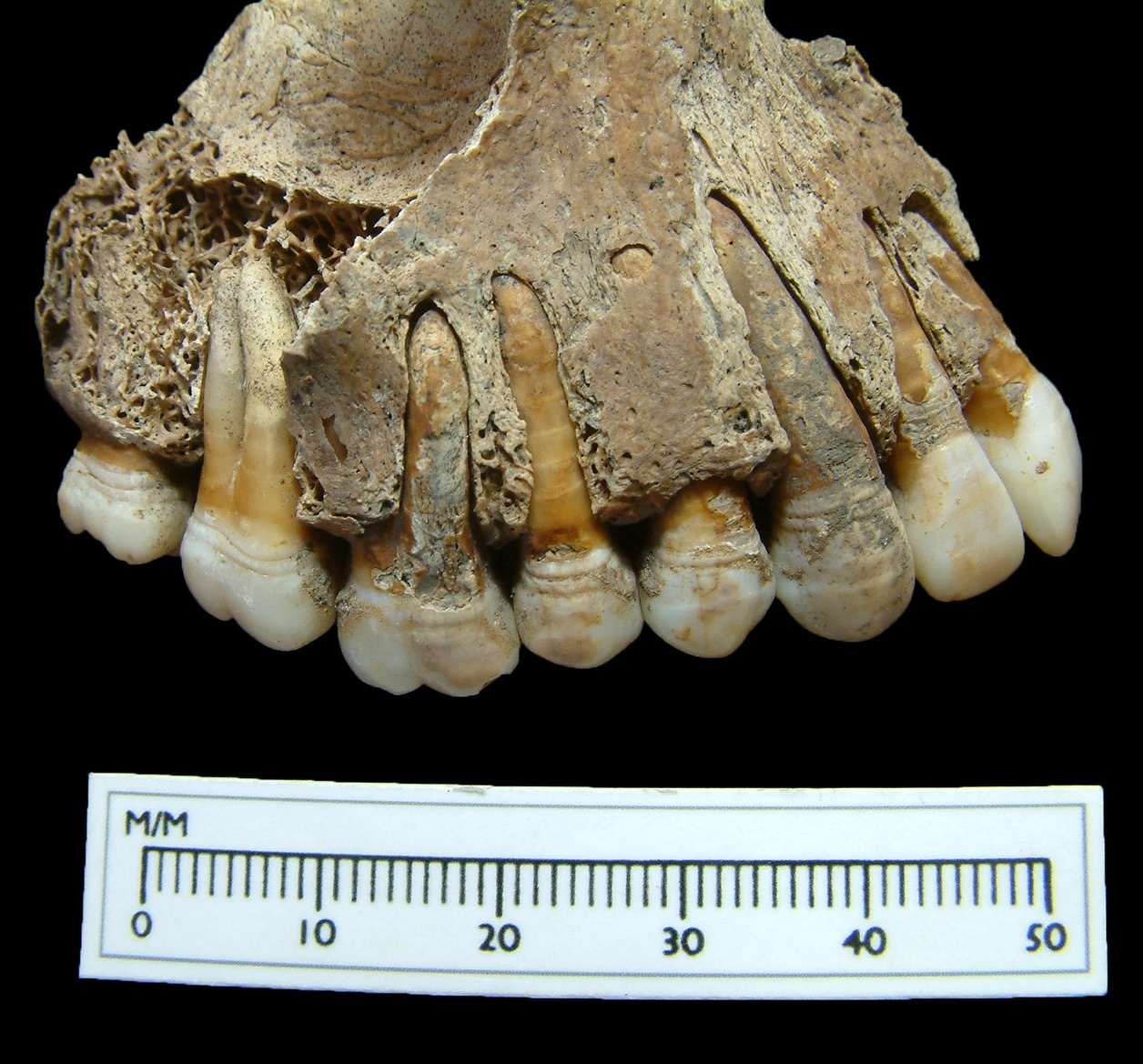


Figure F. Buccal view of Sk 19 maxillary teeth exhibiting enamel hypoplasias


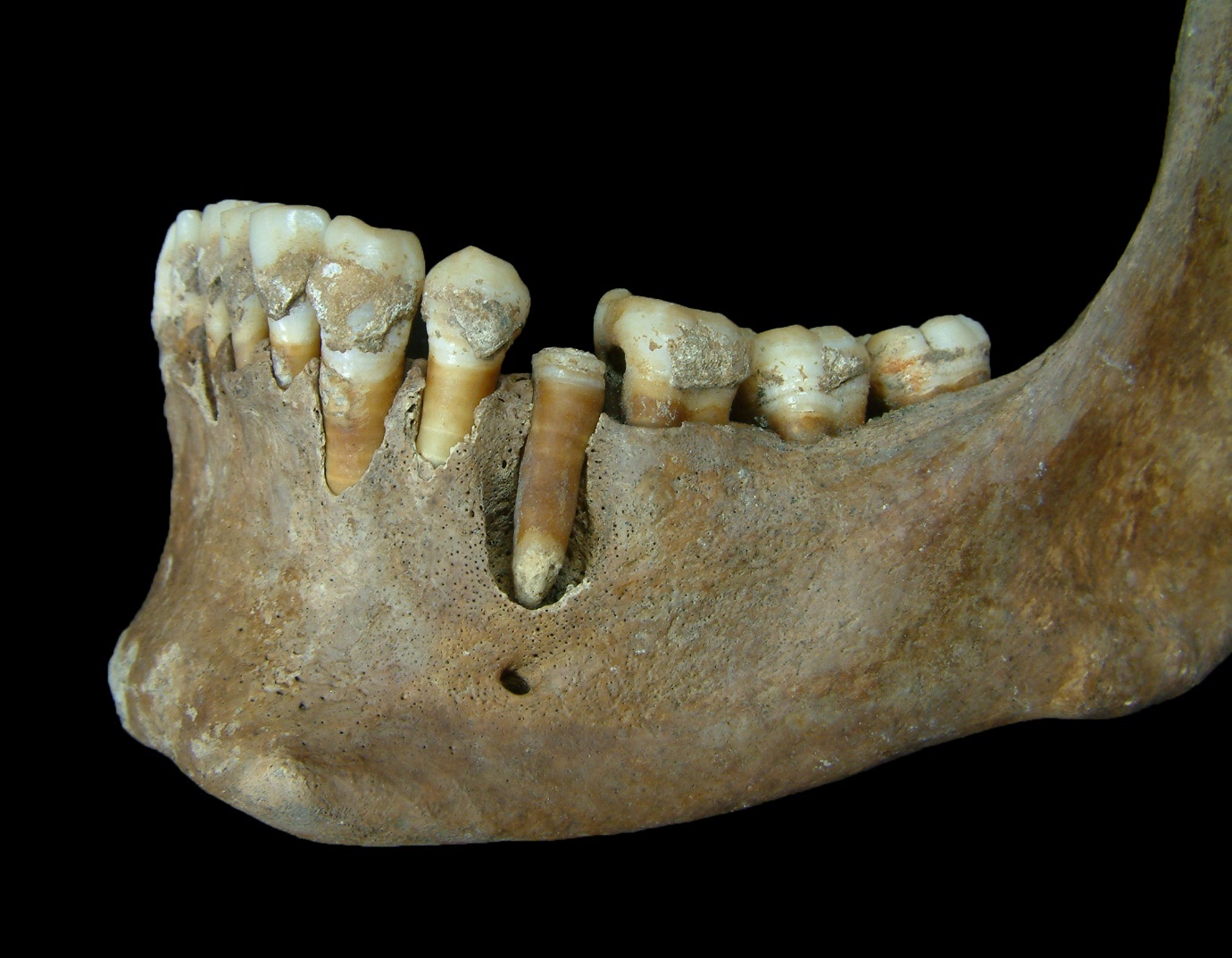


Figure G. Sk 19 lateral view of left mandible showing large abscess around root of the second premolar and caries of second premolar and first molar.


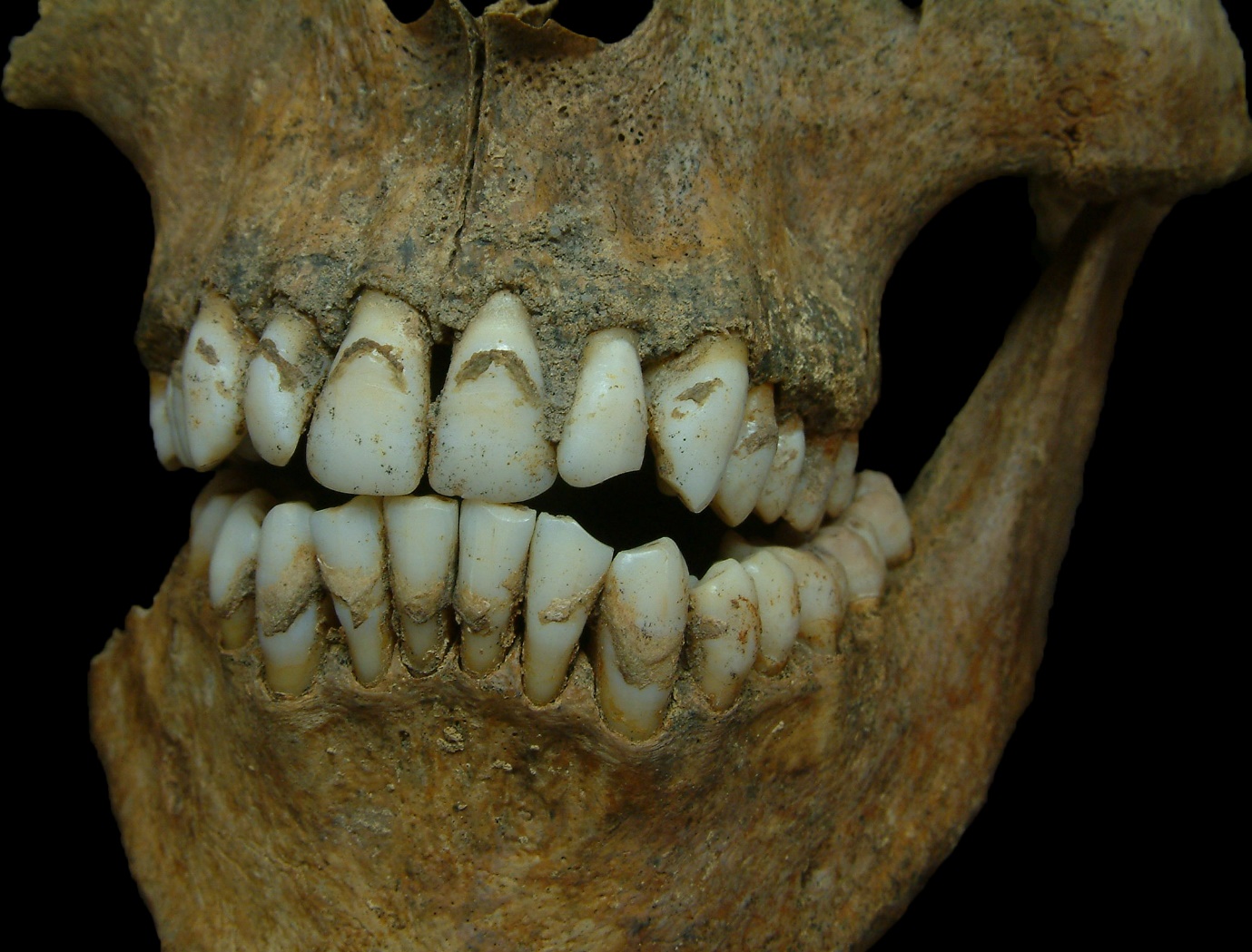


Figure H. anterior view of maxillae and mandible of Sk 21 showing pipe-smoking wear


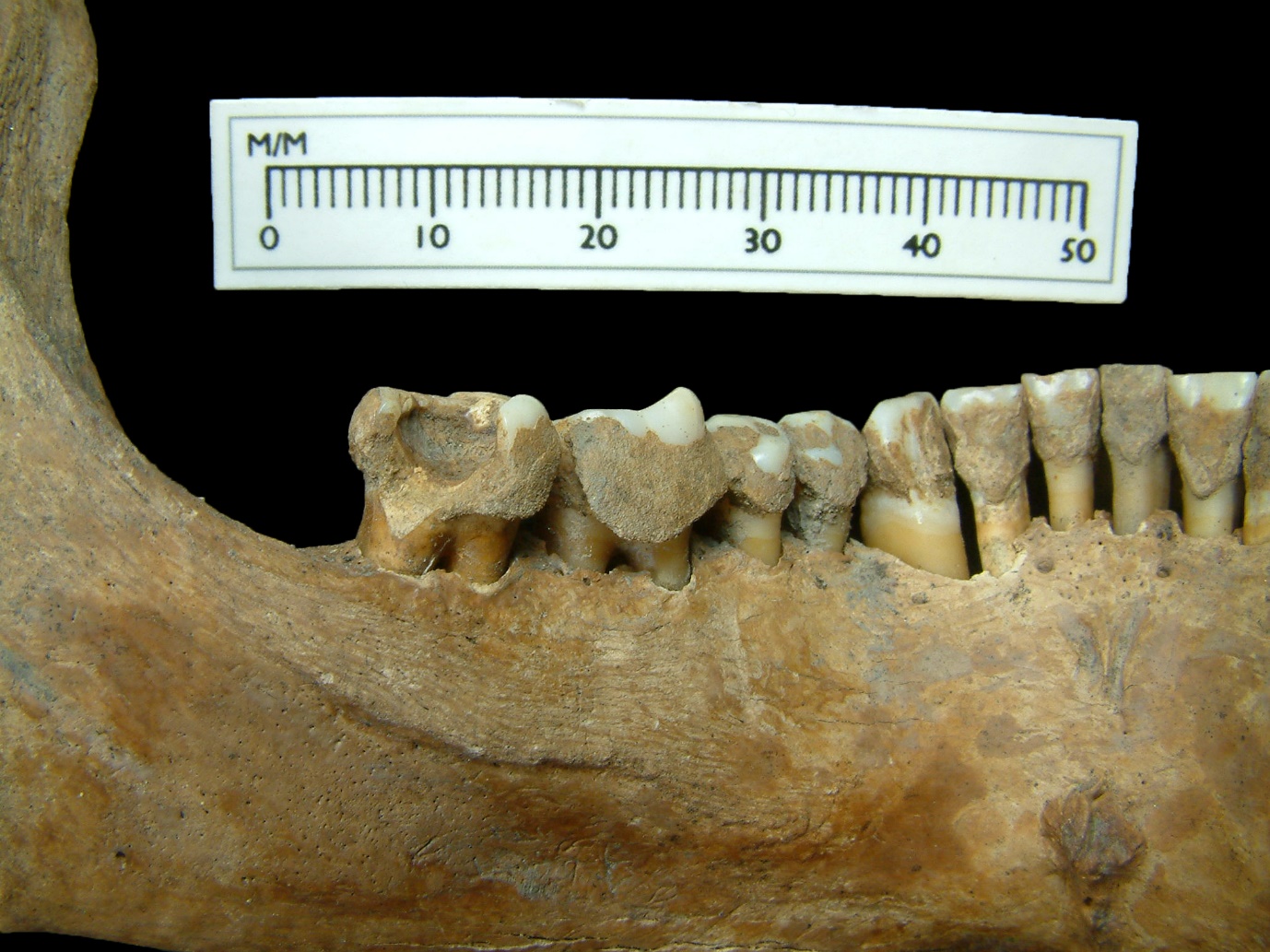


Figure I. Lingual view of left mandible of Sk 22 showing extensive calculus and caries of the second molar.


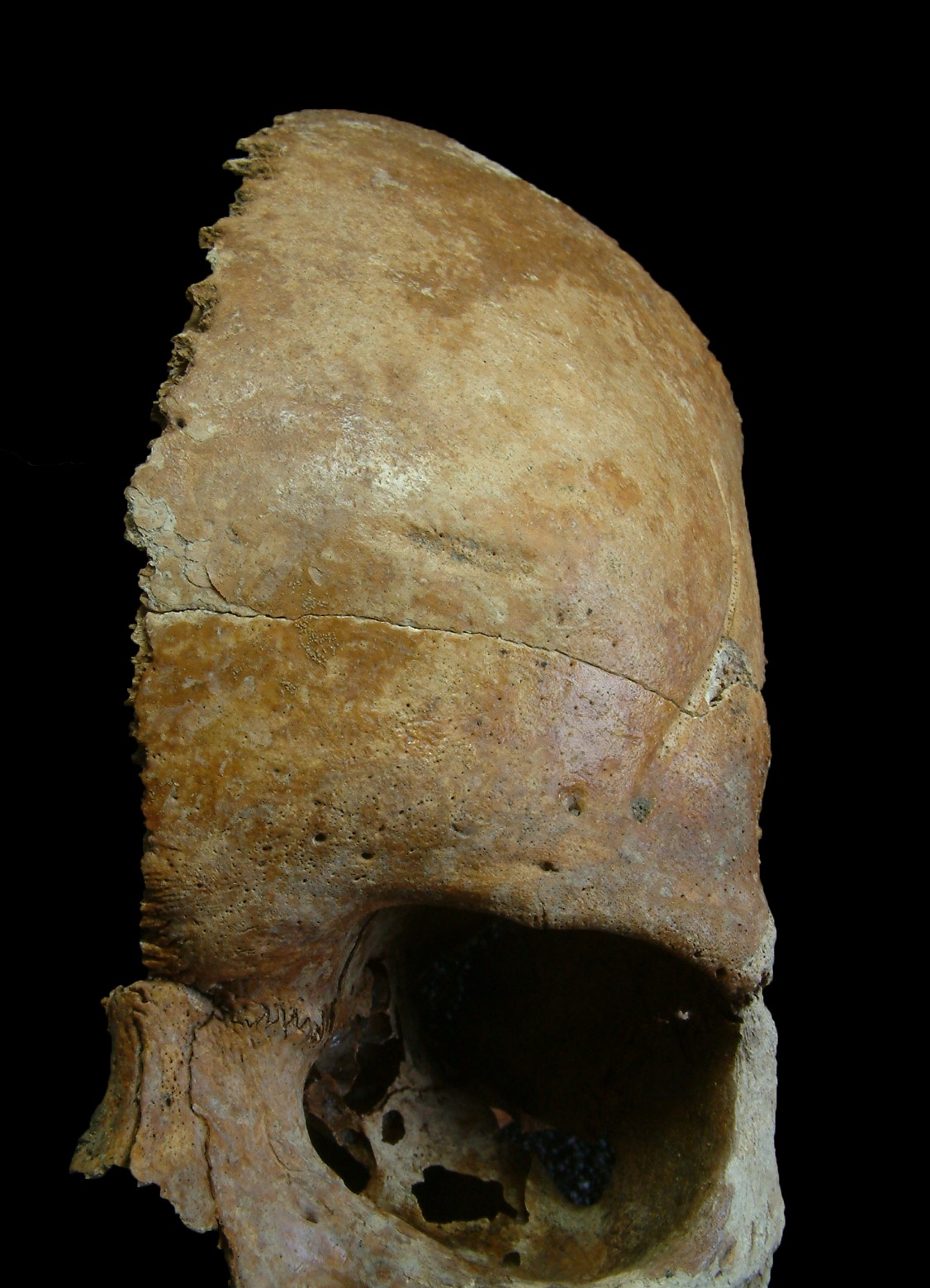


Figure J. Left side of the face of Sk 22 showing blade injury to frontal bone.


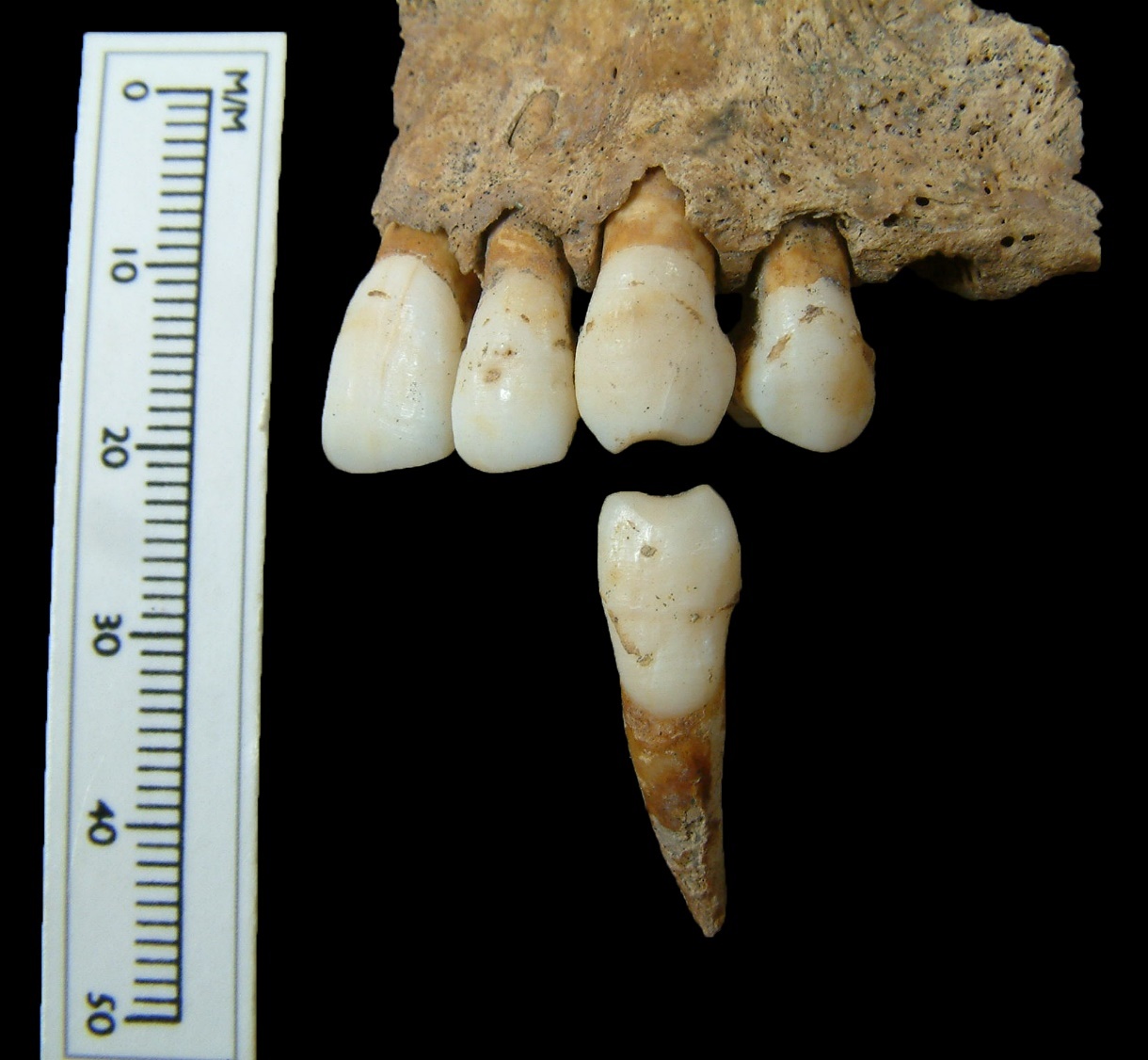


Figure K. Left maxilla and lower left canine of Sk 25 showing unusual wear creating a notch.


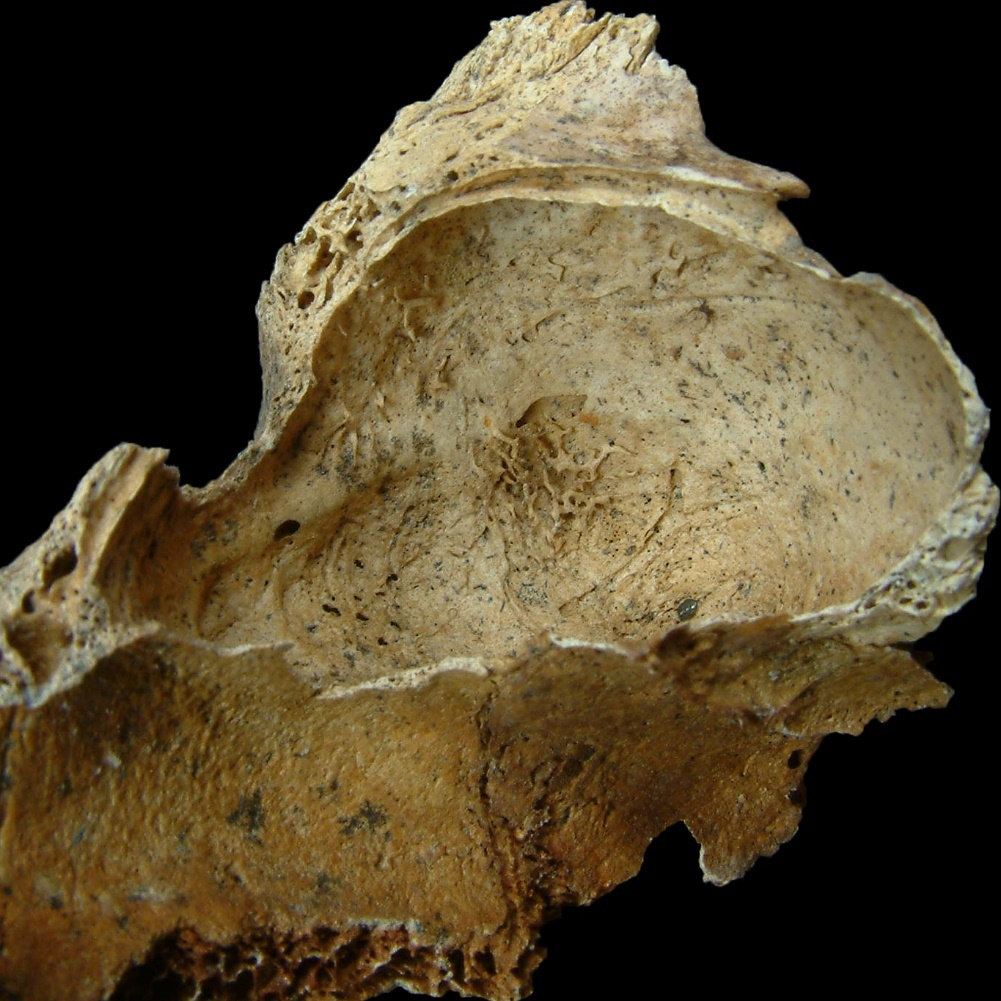


Figure L. Floor of the right maxillary sinus of Sk 27A showing cobweb of lamellar bone strands indicative of sinusitis.


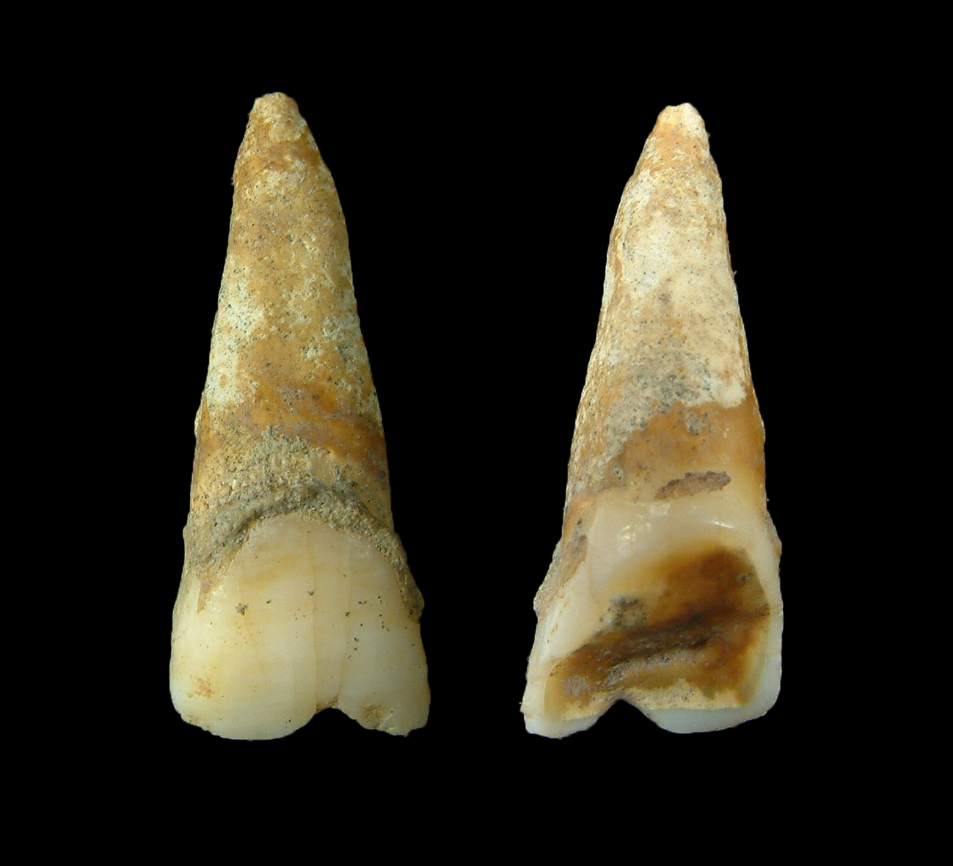


Figure M. Lingual (left) and labial (right) view of upper right first incisor of Sk 28 showing notching and wear to the lingual surface.

## References

1. McKinley JI. Compiling a skeletal inventory: disarticulated and co-mingled remains. In: Brickley M, McKinley JI, editors. Guidelines to the Standards for Recording Human Remains. IFA Paper 7. Reading and Southampton: Institute of Field Archaeologists and British Association for Biological Anthropology and Osteoarchaeology; 2004. p. 14-7.

2. Cox M. Ageing adults from the skeleton. In: Cox M, Mays SAOiAaFS, Greenwich Medical Media, London, 61–82. , editors. Human Osteology in Archaeology and Forensic Science. London: Greenwich Medical Media; 2000. p. 61–82.

3. Brooks ST, Suchey JM. Skeletal age determination based on the os pubis: a comparison of the Ascadi-Nemeskeri and Suchey-Brooks methods. Human Evolution. 1990;5:227-38.

4. Lovejoy CO, Meindl RS, Pryzbeck TR, Mensforth RP. Chronological metamorphosis of the auricular surface of the ilium: a new method for the determination of age at death. American Journal of Physical Anthropology. 1985;68:15-28.

5. İsçan MY, Loth SR, Wright RK. Metamorphosis at the sternal rib end: A new method to estimate age at death in white males. American Journal of Physical Anthropology. 1984;65:147–56.

6. İsçan M, Loth S. Determination of age from the sternal rib in white females: a test of the phase method. Journal of Forensic Science. 1986;31:990-9.

7. İşcan MY, Loth SR, Wright RK. Age estimation from the rib by phase analysis: white females. Journal of Forensic Sciences. 1985;30:853–63.

8. Brothwell DR. Digging Up Bones. Third Edition. Ithaca: Cornell University Press; 1981.

9. Moorrees CFA, Fanning EA, Hunt EE. Formation and resorption of three deciduous teeth in children. American Journal of Physical Anthropology. 1963;21:205-13.

10. Moorrees CFA, Fanning EA, Hunt EE. Age variation of formation stages for ten permanent teeth. Journal of Dental Research. 1963;42:1490-502.

11. Ubelaker DH. Human Skeletal Remains. second ed. Washington, DC: Taraxacum; 1989.

12. Scheuer L, Black S. Developmental juvenile osteology. London: Academic Press; 2000.

13. Mays SA, Cox M. Sex determination in skeletal remains. In: Cox M, Mays SAOiAaFS, Greenwich Medical Media, London, 61–82., editors. Human Osteology in Archaeology and Forensic Science. London: Greenwich Medical Media; 2000. p. 117–30.

14. Trotter M. Estimation of stature from intact long limb bones. In: Stewart TD, editor. Personal Identification in Mass Disasters. Washington DC: Smithsonian Institution; 1970. p. 71–83.

15. Roberts CA, Manchester K. The archaeology of disease. 3rd ed. Stroud: History Press; 2010.

16. Ortner DJ. Identification of pathological conditions in human skeletal remains. San Diego and London: Academic Press; 2003.

17. Liu B, Zhang M, Chen Y, Yao Y. Tooth wear in aging people: an investigation of the prevalence and the influential factors of incisal/occlusal tooth wear in northwest China. BMC Oral Health. 2014;14:65.

18. Hillson S. The Anthropology of Teeth. Cambridge: Cambridge University Press; 1996.

19. Hillson S. Dental anthropology, second edition. Cambridge: Cambridge University Press; 2005.

20. Lozano M, Bermúdez de Castro JM, Carbonell E, Arsuaga JL. Non-masticatory uses of anterior teeth of Sima de los Huesos individuals (Sierra de Atapuerca, Spain). Journal of Human Evolution. 2008;55(4):713-28.

21. Lozano M, Mosquera M, Bermúdez de Castro JM, Arsuaga JL, Carbonell E. Right handedness of Homo heidelbergensis from Sima de los Huesos (Atapuerca, Spain) 500,000 years ago. Evolution and Human Behavior. 2008;30(5):369-76.

22. Cristiani E, Radini A, Edinborough M, Borić D. Dental calculus reveals Mesolithic foragers in the Balkans consumed domesticated plant foods. Proceedings of the National Academy of Sciences. 2016. doi: 10.1073/pnas.1603477113.

23. Henry AG, Hudson HF, Piperno DR. Changes in starch grain morphologies from cooking. Journal of Archaeological Science. 2009;36(3):915-22. doi: DOI: 10.1016/j.jas.2008.11.008.

24. Petraco N, Kubic T. Color atlas and manual of microscopy for criminalists, chemists, and conservators. Abingdon: CRC Press; 2003.

25. Radini A, Nikita E, Shillito LM. Human dental calculus and a Medieval urban environment. In: Jervis B, Broderick L, Sologestoa IG, editors. Objects, environment, and everyday life in Medieval Europe. Turnhout, Belgium: BREPOLS; 2016. p. 297-313.

26. Charlier BLA, Ginibre C, Morgan D, Nowell GM, Pearson DG, Davidson JP, et al. Methods for the microsampling and high-precision analysis of strontium and rubidium isotopes at single crystal scale for petrological and geochronological applications. Chemical Geology. 2006;232(3-4):114-33. PubMed PMID: ISI:000240214100003.

27. Smits E, Millard AR, Nowell G, Pearson DG. Isotopic investigation of diet and residential mobility in the Neolithic of the Lower Rhine Basin. European Journal of Archaeology. 2010;13:5-31.

28. Evans JA, Montgomery J, Wildman G, Boulton N. Spatial variations in biosphere ^87^Sr/^86^Sr in Britain. Journal of the Geological Society. 2010;167(1):1-4. doi: 10.1144/0016-76492009-090.

29. Snoeck C, Ryan S, Pouncett J, Pellegrini M, Claeys P, Wainwright AN, et al. Towards a biologically available strontium isotope baseline for Ireland. Science of The Total Environment. 2019:136248. doi: <https://doi.org/10.1016/j.scitotenv.2019.136248>.

30. Dettman DL, Kohn MJ, Quade J, Ryerson FJ, Ojha TP, Hamidullah S. Seasonal stable isotope evidence for a strong Asian monsoon throughout the past 10.7 m.y. Geology. 2001;29(1):31-4. PubMed PMID: ISI:000166441700008.

31. Fourel F, Martineau F, Lécuyer C, Kupka H-J, Lange L, Ojeimi C, et al. ^18^O/^16^O ratio measurements of inorganic and organic materials by elemental analysis–pyrolysis–isotope ratio mass spectrometry continuous‐flow techniques. Rapid Communications in Mass Spectrometry. 2011;25:2691-6.

32. Lécuyer C, Grandjean P, Oneil JR, Cappetta H, Martineau F. Thermal excursions in the ocean at the Cretaceous-Tertiary boundary (northern Morocco) - delta-O-18 record of phosphatic fish debris. Palaeogeography Palaeoclimatology Palaeoecology. 1993;105(3-4):235-43. PubMed PMID: WOS:A1993MR35400004.

33. Chenery C, Müldner G, Evans J, Eckardt H, Lewis M. Strontium and stable isotope evidence for diet and mobility in Roman Gloucester, UK. Journal of Archaeological Science. 2010;37(1):150-63. doi: 10.1016/j.jas.2009.09.025.

34. Daux V, Lécuyer C, Héran M-A, Amiot R, Simon L, Fourel F, et al. Oxygen isotope fractionation between human phosphate and water revisited. Journal of Human Evolution. 2008;55(6):1138-47.

35. Darling WG, Bath AH, Talbot JC. The O and H Stable Isotopic Content of Fresh Waters in The British Isles: 2. ground-water and surface waters. Hydrology and Earth System Sciences. 2003;7:183-95.

36. Darling WG, Talbot JC. The O & H stable isotopic composition of fresh waters in the British Isles. 1. Rainfall. Hydrology and Earth System Sciences. 2003;7(2):163-81.

37. Bowen GJ, Revenaugh J. Interpolating the isotopic composition of modern meteoric precipitation. Water Resources Research. 2003;39(10):1299 doi:10.129/2003WR002086.

38. Longin R. New method of collagen extraction for radiocarbon dating. Nature. 1971;230:241-2.

39. Beaumont J, Gledhill A, Lee-Thorp J, Montgomery J. Childhood diet: a closer examination of the evidence from dental tissues using stable isotope analysis of incremental human dentine. Archaeometry. 2013;55(2):277-95. doi: 10.1111/j.1475-4754.2012.00682.x. PubMed PMID: WOS:000314983900006.

40. Beaumont J, Montgomery J. Oral histories: a simple method of assigning chronological age to isotopic values from human dentine collagen. Annals of Human Biology. 2015;42(4):407-14. doi: 10.3109/03014460.2015.1045027.

41. AlQahtani SJ, Hector MP, Liversidge HM. Brief communication: The London atlas of human tooth development and eruption. American Journal of Physical Anthropology. 2010;142(3):481-90. doi: 10.1002/ajpa.21258.

42. R F, M KB. Gel-aided samplepreparation (GASP) - a simplified method for gel-assisted proteomic sample generation from protein extracts and intact cells. Proteomics. 2015;15:1224-9. doi: 10.1002/pmic.201400436.

43. Hendy J, Warinner C, Bouwman A, Collins MJ, Fiddyment S, Fischer R, et al. Proteomic evidence of dietary sources in ancient dental calculus. Proceedings of the Royal Society B: Biological Sciences. 2018;285(1883). doi: 10.1098/rspb.2018.0977.

44. Warinner C, Hendy J, Speller C, Cappellini E, Fischer R, Trachsel C, et al. Direct evidence of milk consumption from ancient human dental calculus. Scientific Reports. 2014;4:7104. doi: 10.1038/srep07104.

45. Huson DH, Beier S, Flade I, Górska A, El-Hadidi M, Mitra S, et al. MEGAN Community Edition - Interactive Exploration and Analysis of Large-Scale Microbiome Sequencing Data. PLOS Computational Biology. 2016;12:e1004957.

46. Shoulders MD, Raines RT. Collagen structure and stability. Annual Review of Biochemistry. 2009;78:929-58.

47. Peterkofsky B. Ascorbate requirement for hydroxylation and secretion of procollagen: relationship to inhibition of collagen synthesis in scurvy. The American Journal of Clinical Nutrition. 1991;54(6):1135S-40S.

48. Sato P, Udenfriend S. Scurvy-prone animals, including man, monkey, and guinea pig, do not express the gene for gulonolactone oxidase. Archives of Biochemistry and Biophysics. 1978;187(1):158-62.

49. Tsuchiya H, Bates CJ. Comparison of vitamin C deficiency with food restriction on collagen cross-link ratios in bone, urine and skin of weanling guinea-pigs. British Journal of Nutrition. 2003;89(3):303-10.

50. Chojkier M, Spanheimer R, Peterkofsky B. Specifically decreased collagen biosynthesis in scurvy dissociated from an effect on proline hydroxylation and correlated with body weight loss. In vitro studies in guinea pig calvarial bones. Journal of Clinical Investigation. 1983;72(3):826-35.

51. Chopra RK, Ananthanarayanan VS. Conformational implications of enzymatic proline hydroxylation in collagen. Proceedings of the National Academy of Sciences. 1982;79(23):7180-4.

52. Rigby BJ. Amino-acid composition and thermal stability of the skin collagen of the Antarctic ice-fish. Nature. 1968;219(5150):166-7.

53. Holmgren SK, Taylor KM, Bretscher LE, Raines RT. Code for collagen's stability deciphered. Nature. 1998;392(6677):666-7.

54. Bella J, Brodsky B, Berman HM. Hydration structure of a collagen peptide. Structure. 1995;3(9):893-906.

55. Vitagliano L, Berisio R, Mazzarella L, Zagari A. Structural bases of collagen stabilization induced by proline hydroxylation. Biopolymers. 2001;58(5):459-64.

56. Burjanadze TV. New analysis of the phylogenetic change of collagen thermostability. Biopolymers. 2000;53(6):523-8.

57. Berg RA, Prockop DJ. The thermal transition of a non-hydroxylated form of collagen. Evidence for a role for hydroxyproline in stabilizing the triple-helix of collagen. Biochemical and Biophysical Research Ccommunications. 1973;52(1):115-20.

58. Ortner DJ, Butler W, Cafarella J, Milligan L. Evidence of probable scurvy in subadults from archeological sites in North America. American Journal of Physical Anthropology. 2001;114(4):343-51.

59. Pimentel L. Scurvy: historical review and current diagnostic approach. American Journal of Emergency Medicine. 2003;21(4):328-32.

60. Brickley M, Ives R. Skeletal manifestations of infantile scurvy. American Journal of Physical Anthropology. 2006;129(2):163-72.

61. Buckley M, Collins MJ. Collagen survival and its use for species identification in Holocene-lower Pleistocene bone fragments from British archaeological and paleontological sites. Antiquaries Journal. 2011;1(1):e1-e.

62. Buckley M, Collins M, Thomas-Oates J, Wilson JC. Species identification by analysis of bone collagen using matrix-assisted laser desorption/ionisation time-of-flight mass spectrometry. Rapid Communications in Mass Spectrometry. 2009;23(23):3843-54. doi: 10.1002/rcm.4316. PubMed PMID: WOS:000272522700030.

63. Welker F, Soressi M, Rendu W, Hublin J-J, Collins M. Using ZooMS to identify fragmentary bone from the Late Middle/Early Upper Palaeolithic sequence of Les Cottés, France. Journal of Archaeological Science. 2015;54:279-86. doi: <http://dx.doi.org/10.1016/j.jas.2014.12.010>.

64. Cappellini E, Jensen LJ, Szklarczyk D, Ginolhac A, da Fonseca RA, Stafford TW, et al. Proteomic analysis of a pleistocene mammoth femur reveals more than one hundred ancient bone proteins. Journal of Proteome Research. 2012;11(2):917-26.

65. Ewles M, Goodwin L. Bioanalytical approaches to analyzing peptides and proteins by LC–MS/MS. Bioanalysis. 2011;3(12):1379-97.

66. Wadsworth C, Buckley M. Proteome degradation in fossils: investigating the longevity of protein survival in ancient bone. Rapid Communications in Mass Spectrometry. 2014;28(6):605-15. doi: 10.1002/rcm.6821.

67. Welker F, Collins MJ, Thomas JA, Wadsley M, Brace S, Cappellini E, et al. Ancient proteins resolve the evolutionary history of Darwin’s South American ungulates. Nature. 2015;522:81. doi: 10.1038/nature14249

<https://www.nature.com/articles/nature14249#supplementary-information>.

68. Koon HE. A biochemical marker for scurvy in archaeological bones. American Journal of Physical Anthropology. 2012;147:184-5.

69. Montgomery H, Rustogi N, Hadjisavvas A, Tanaka K, Kyriacou K, Sutton CW. Proteomic profiling of breast tissue collagens and site-specific characterization of hydroxyproline residues of collagen alpha-1-(I). Journal of Proteome Research. 2012;11(12):5890-902.

70. Annis R. Palace Green LIbarary internal courtyard, Durhamj City, archaeological monitoring, interim report. Durham: Archaeological Services Durham University; 2015.

71. Millard AR. Palace Green Library excavations 2013 (PGL13): Chronology of the burials. Durham: Durham University, 2015.
