# Supplementary material for: Scottish soldiers from the Battle of Dunbar 1650: A prosopographical approach to a skeletal assemblage
Source: PLoS One. 2020 Dec 21;15(12):e0243369. doi: 10.1371/journal.pone.0243369 (PMC7751964; doi:10.1371/journal.pone.0243369)
Supplement: S1 Table — (DOCX) [file pone.0243369.s002.docx]

### The Scottish soldiers from the Battle of Dunbar 1650: a prosopographical approach to a skeletal assemblage

### S1 Table: Strontium, oxygen and lead isotope results

Strontium, oxygen and lead isotope data on M2 previously appeared in [1] and these and strontium isotope ratios for M1 and M3 were plotted with a preliminary analysis in [2].

### (a) Strontium and oxygen isotopes

|  | **M1** | | | | | **M2** | | | | | **M3** | | | | |
| --- | --- | --- | --- | --- | --- | --- | --- | --- | --- | --- | --- | --- | --- | --- | --- |
|  | **Tooth** | **^87^Sr/^86^Sr** | **± 2SE** | **δ^18^O_P_  VSMOW** | **δ^18^O_DW_  VSMOW** | **Tooth** | **^87^Sr/^86^Sr** | **± 2SE** | **δ^18^O_P_  VSMOW** | **δ^18^O_DW_  VSMOW** | **Tooth** | **^87^Sr/^86^Sr** | **± 2SE** | **δ^18^O_P_  VSMOW** | **δ^18^O_DW_  VSMOW** |
| Sk 1 | URM1 | 0.710534 | 0.000010 | 18.90 | -4.62 | ULM2 | 0.710702 | 0.000012 | 18.16 | -5.76 | URM3 | 0.710275 | 0.000009 | 16.11 | -8.91 |
| Sk 2 | LLM1 | 0.709177 | 0.000012 | 18.17 | -5.74 | LLM2 | 0.709211 | 0.000018 | 18.69 | -4.94 | URM3 | 0.709167 | 0.000013 | 17.46 | -6.84 |
| Sk 5 | URM1 | 0.708618 | 0.000013 | 19.04 | -4.39 | LLM2 | 0.708587 | 0.000014 | 15.56 | -9.75 | URM3 | 0.708691 | 0.000012 | 15.88 | -9.27 |
| Sk 6 | ULM1 | 0.70992 | 0.000013 | 18.80 | -4.76 | LLM2 | 0.710141 | 0.000010 | 19.19 | -4.17 | ULM3 | 0.710235 | 0.000011 | 18.94 | -4.55 |
| Sk 12 | LLM1 | 0.709635 | 0.000008 | 18.29 | -5.56 | LRM2 | 0.709758 | 0.000012 | failed | | ULM3 | 0.709709 | 0.000009 | 16.28 | -8.64 |
| Sk 19 | ULM1 | 0.710095 | 0.000010 | 17.54 | -6.70 | LLM2 | 0.710037 | 0.000014 | 17.54 | -6.70 | ULM3 | 0.710237 | 0.000009 | 16.26 | -8.68 |
| Sk 21 | URM1 | 0.710001 | 0.000012 | 19.37 | -3.89 | LRM2 | 0.709967 | 0.000012 | 16.09 | -8.94 | URM3 | 0.709911 | 0.000011 | 17.56 | -6.68 |
| Sk 22 | LRM1 | 0.713262 | 0.000010 | 17.53 | -6.73 | URM2 | 0.713542 | 0.000016 | 19.11 | -4.29 | LRM3 | 0.713891 | 0.000012 | 17.11 | -7.38 |
| Sk 23 | LRM1 | 0.710358 | 0.000011 | 17.61 | -6.61 | LLM2 | 0.710592 | 0.000010 | 17.05 | -7.46 | LRM3 | 0.711023 | 0.000010 | 16.34 | -8.56 |
| Sk 24 | LLM1 | 0.7088 | 0.000011 | 19.01 | -4.45 | LLM2 | 0.708802 | 0.000012 | 14.71 | -11.06 | LLM3 | 0.708735 | 0.000011 | 16.03 | -9.03 |
| Sk 25 | ULM1 | 0.709743 | 0.000010 | 19.36 | -3.91 | URM2 | 0.709760 | 0.000014 | 19.32 | -3.97 | ULM3 | 0.70911 | 0.000013 | 18.73 | -4.87 |
| SK 27A | LRM1 | 0.712212 | 0.000012 | 16.45 | -8.38 | LRM2 | 0.714684 | 0.000012 | 15.22 | -10.28 | no M3 available | | | | |
| Sk 28 | LRM1 | 0.709878 | 0.000008 | 18.11 | -5.83 | ULM2 | 0.709943 | 0.000012 | 19.17 | -4.20 | URM3 | 0.710488 | 0.000007 | 17.59 | -6.64 |

Drinking water values calculated from δ^18^O_P_ using equation 6 of Daux et al. [3]

### (b) Lead isotope ratios measured on M2

| **Skeleton** | **Pb/ppb** | **^206^Pb/^204^Pb** | **2SE** | **^207^Pb/^204^Pb** | **2SE** | **^208^Pb/^204^Pb** | **2SE** | **^207^Pb/^206^Pb** | **2SE** | **^208^Pb/^206^Pb** | **2SE** |
| --- | --- | --- | --- | --- | --- | --- | --- | --- | --- | --- | --- |
| Sk1 | 165 | 18.45277 | 0.00095 | 15.62526 | 0.00103 | 38.43148 | 0.00361 | 0.846770 | 0.000020 | 2.082745 | 0.000104 |
| Sk2 | 107 | concentration too low to measure | | |  |  |  |  |  |  |  |
| Sk5 | 257 | 18.44272 | 0.00092 | 15.62407 | 0.00102 | 38.4223 | 0.0033 | 0.847174 | 0.000017 | 2.083348 | 0.000091 |
| Sk6 | 518 | 18.42591 | 0.00062 | 15.62337 | 0.00068 | 38.4169 | 0.0028 | 0.847909 | 0.000014 | 2.084913 | 0.000091 |
| Sk12 | 588 | 18.28169 | 0.00068 | 15.58699 | 0.00075 | 38.2177 | 0.0029 | 0.852609 | 0.000012 | 2.090479 | 0.000088 |
| Sk19 | 129 | 18.39871 | 0.00088 | 15.61614 | 0.00092 | 38.3790 | 0.0031 | 0.848758 | 0.000016 | 2.085972 | 0.000092 |
| Sk21 | 216 | 18.38430 | 0.00075 | 15.61183 | 0.00085 | 38.3553 | 0.0033 | 0.849196 | 0.000015 | 2.086292 | 0.000090 |
| Sk22 | 187 | 18.45954 | 0.00120 | 15.60964 | 0.00107 | 38.3674 | 0.0031 | 0.845615 | 0.000019 | 2.078491 | 0.000098 |
| Sk23 | 445 | 18.38158 | 0.00127 | 15.61324 | 0.00125 | 38.3500 | 0.0037 | 0.849400 | 0.000015 | 2.086317 | 0.000084 |
| Sk24 | 295 | 18.40472 | 0.00072 | 15.61664 | 0.00077 | 38.3783 | 0.0025 | 0.848513 | 0.000012 | 2.085251 | 0.000077 |
| Sk25 | 714 | 18.20864 | 0.00069 | 15.55694 | 0.00073 | 38.1064 | 0.0031 | 0.854377 | 0.000012 | 2.092777 | 0.000104 |
| SK27A | 154 | 18.44800 | 0.00112 | 15.62093 | 0.00098 | 38.4178 | 0.0024 | 0.846762 | 0.000018 | 2.082550 | 0.000083 |
| Sk28 | 156 | 18.38204 | 0.00085 | 15.61578 | 0.00083 | 38.3488 | 0.0028 | 0.849518 | 0.000015 | 2.086260 | 0.000076 |
| NBS981 reference values | | 16.94160 |  | 15.49990 |  | 36.7258 |  | 0.914902 |  | 2.167788 |  |
| NBS981 measured | | 16.94175 | 0.00174 | 15.49856 | 0.00183 | 36.7171 | 0.0062 | 0.914807 | 0.000041 | 2.167250 | 0.000157 |

### References

1. Millard AR. Palace Green Library excavations 2013 (PGL13): Isotopic studies. Durham: Durham University, 2015.

2. Gerrard C, Graves P, Millard A, Annis R, Caffell A. Lost Lives, New Voices: Unlocking the stories of the Scottish Soldiers from the Battle of Dunbar 1650. Oxford: Oxbow; 2018.

3. Daux V, Lécuyer C, Héran M-A, Amiot R, Simon L, Fourel F, et al. Oxygen isotope fractionation between human phosphate and water revisited. Journal of Human Evolution. 2008;55(6):1138-47.
