# Supplementary material for: Scottish soldiers from the Battle of Dunbar 1650: A prosopographical approach to a skeletal assemblage
Source: PLoS One. 2020 Dec 21;15(12):e0243369. doi: 10.1371/journal.pone.0243369 (PMC7751964; doi:10.1371/journal.pone.0243369)
Supplement: S4 Table — (DOCX) [file pone.0243369.s005.docx]

### The Scottish soldiers from the Battle of Dunbar 1650: a prosopographical approach to a skeletal assemblage

### S4 Table: Putative dietary peptides identified within the dental calculus samples

| Individual | Protein | Taxon | Peptide | Mascot Score |
| --- | --- | --- | --- | --- |
| PGL1 | Patatin-like protein 7 | Brassicaceae | K.RIDEQSNFEK.L + Propionamide (K) | 28 |
|  |  |  | R.IDEQSNFEK.L + Propionamide (K) | 50 |
|  | Legumin type B | *Vicia faba* | R.ATPADVLANAFGLR.Q | 60 |
|  |  |  | R.ATPADVLANAFGLR.Q + Deamidated (NQ) | 28 |
|  | 12S seed storage globulin 1 | *Avena sativa* | R.LQAFEPLR.Q | 33 |
|  |  |  | R.SQAGITEYFDEQNEQFR.C | 63 |
|  |  |  | R.EGCQYISFK.T | 45 |
| PGL2 | Beta-lactoglobulin | Bovinae | R.TPEVDDEALEKFDK.A | 32 |
|  |  |  | K.ALPMHIR.L | 28 |
|  |  |  | R.LSFNPTQLEEQCHI.- | 65 |
| PGL12 | 12S seed storage globulin 1 | *Avena sativa* | R.ALPVDVLANAYR.I | 59 |
|  |  |  | R.EGCQYISFK.T | 49 |
| PGL21 | Beta-lactoglobulin | Bovinae | R.TPEVDDEALEK.F | 51 |
|  |  |  | R.TPEVDDEALEKFDK.A | 32 |
|  |  |  | R.LSFNPTQLEEQCHI.- | 45 |
|  |  |  | R.LSFNPTQLEEQCHI.- | 57 |
|  |  |  | R.LSFNPTQLEEQCHI.- + Deamidated (NQ) | 49 |
|  | 12S seed storage globulin 1 | *Avena sativa* | R.SQAGITEYFDEQNEQFR.C | 78 |
|  |  |  | R.RVIEPQGLLLPQYHNAPGLVYILQGR.G | 38 |
|  |  |  | R.ALPVDVLANAYR.I | 35 |
| PGL22 | 12S seed storage globulin 1 | *Avena sativa* | R.ALPVDVLANAYR.I | 53 |
|  |  |  | R.ALPVDVLANAYR.I + Deamidated (NQ) | 56 |
| PGL25 | 12S seed storage globulin 1 | *Avena sativa* | R.SQAGITEYFDEQNEQFR.C | 103 |
|  |  |  | R.EGCQYISFK.T | 33 |
|  |  |  | R.ALPVDVLANAYR.I | 59 |
| PGL28 | Alpha-amylase/trypsin inhibitor CM3 | Triticinae | R.SGNVGESGLIDLPGCPR.E | 55 |
|  |  |  | R.LLVAPGQCNLATIHNVR.Y | 50 |
