# Supplementary material for: Scottish soldiers from the Battle of Dunbar 1650: A prosopographical approach to a skeletal assemblage
Source: PLoS One. 2020 Dec 21;15(12):e0243369. doi: 10.1371/journal.pone.0243369 (PMC7751964; doi:10.1371/journal.pone.0243369)
Supplement: S1 Fig — (DOCX) [file pone.0243369.s006.docx]

### The Scottish soldiers from the Battle of Dunbar 1650: a prosopographical approach to a skeletal assemblage

### S1 Fig: Incremental dentine δ^15^N and δ^13^C collagen profiles for permanent canine and M3 for each individual by approximate age at of formation

1. Sk 1

1. Sk 2

1. Sk 5

1. Sk 6

1. Sk 12

1. Sk 19

1. Sk 21

1. Sk 22

1. Sk 23

1. Sk 24

1. Sk 25

1. Sk 28
